# Prevalence of colistin resistance in clinical isolates of *Acinetobacter baumannii*: a systematic review and meta-analysis

**DOI:** 10.1186/s13756-024-01376-7

**Published:** 2024-02-28

**Authors:** Narjess Bostanghadiri, Negar Narimisa, Maryam Mirshekar, Leila Dadgar-Zankbar, Elahe Taki, Tahereh Navidifar, Davood Darban-Sarokhalil

**Affiliations:** 1 Department of Microbiology, School of Medicine, Iran University of Medical Sciences, Tehran, Iran; 2 Department of Microbiology, School of Medicine, Kermanshah University of Medical Science, Kermanshah, Iran; 3 Department of Basic Sciences, Shoushtar Faculty of Medical Sciences, Shoushtar, Iran

**Keywords:** *Acinetobacter baumannii*, *Acinetobacter*, Colistin resistance, Global prevalence, Systematic review, Meta-analysis

## Abstract

**Introduction:**

The development of colistin resistance in *Acinetobacter baumannii* during treatment has been identified in certain patients, often leading to prolonged or recurrent infections. As colistin, is the last line of therapy for *A. baumannii* infections that are resistant to almost all other antibiotics, colistin-resistant *A. baumannii* strains currently represent a significant public health threat, particularly in healthcare settings where there is significant selective pressure.

**Aim:**

The aim of this study was to comprehensively determine the prevalence of colistin resistance in *A. baumannii* from clinical samples. Regional differences in these rates were also investigated using subgroup analyses.

**Method:**

The comprehensive search was conducted using “*Acinetobacter baumannii*”, “Colistin resistant” and all relevant keywords. A systematic literature search was performed after searching in PubMed, Embase, Web of Science, and Scopus databases up to April 25, 2023. Statistical analysis was performed using Stata software version 17 and sources of heterogeneity were evaluated using I^2^. The potential for publication bias was explored using Egger's tests. A total of 30,307 articles were retrieved. After a thorough evaluation, 734 studies were finally eligible for inclusion in the present systematic review and meta-analysis.

**Result:**

According to the results, the prevalence of resistance to colistin among *A. baumannii* isolates was 4% (95% CI 3–5%), which has increased significantly from 2% before 2011 to 5% after 2012. South America had the highest resistance rate to this antibiotic. The broth microdilution method had the highest level of resistance, while the agar dilution showed the lowest level.

**Conclusions:**

This meta-analysis found a low prevalence of colistin resistance among *A. baumannii* isolates responsible for infections worldwide from 2000 to 2023. However, there is a high prevalence of colistin-resistant isolates in certain countries. This implies an urgent public health threat, as colistin is one of the last antibiotics available for the treatment of infections caused by XDR strains of *A. baumannii*.

**Supplementary Information:**

The online version contains supplementary material available at 10.1186/s13756-024-01376-7.

## Introduction

Multidrug-resistant (MDR) *Acinetobacter baumannii* (*A. baumannii*) is a serious cause of healthcare-associated infections worldwide, often manifesting as hospital-acquired pneumonia and bloodstream infections. With the emergence of isolates resistant to the most available prescription antibiotics, the treatment of MDR- and extensively drug-resistant (XDR)- *A. baumannii* infections has become more challenging [1]. Several antimicrobial agents have been considered for the treatment of these infections, including colistin, sulbactam and tigecycline, which can be used alone or in combination [2]. Colistin is a narrow-spectrum bactericidal molecule that is active against most gram-negative bacteria but without activity against gram-positive bacteria, and anaerobic bacteria [3]. The primary target of colistin is the LPS of gram-negative bacteria membranes [4]. A common cause of resistance to colistin is often a chromosomal mutation in genes associated with the modification of LPS lipid A, which is the primary target of colistin and serves as an adaptive mechanism. These modifications are associated with a mutation in the *pmrA/pmrB* genes and the incorporation of the cationic group phosphoethanolamine (pETN) into lipid A [5]. Other changes include loss or reduction of LPS synthesis due to a mutation in the LpxACD operon, a decrease in biotin synthesis as a cofactor in LPS production, and structural alterations in LPS ring synthesis as the result of a mutation in the *lpsB* gene. Decreases in proteins involved in the export and/or stabilization of outer membrane precursors, such as LptD which is involved in LPS insertion into the outer membrane and the Vps/VacJ ATP-binding cassette (ABC) transporter system which is involved in outer membrane symmetry are other mechanisms resistant to colistin [6]. On the other hand, the emergence of colistin heteroresistance, originally documented by Li et al in 2006 [7], appears to be frequent but also highly variable in different studies. The development of colistin resistance by colistin-susceptible heteroresistant *A. baumannii* during treatment has been identified in certain patients, often leading to prolonged or recurrent *A. baumannii* infections [8]. As polymyxins, including colistin, are the "last line" of therapy for *A. baumannii* infections that are resistant to almost all other antibiotics, colistin-resistant *A. baumannii* strains are currently a significant public health threat. These strains are becoming increasingly prevalent, particularly in healthcare settings where there is significant selective pressure for bacteria [6]. The emergence of colistin-resistant *A. baumannii* is particularly significant in developing countries, where the lack of effective diagnostics and treatments exacerbates its impact. In these contexts, the lack of new antimicrobial drugs forces healthcare professionals to rely on increased use of colistin, which is an important factor in increased resistance to colistin in developing countries [9]. Despite the identification of colistin-resistant *A. baumannii* isolates in healthcare settings worldwide through numerous studies, the prevalence of these infections is still limited to sporadic studies, and an overall analysis of the distribution and incidence patterns of colistin-resistant *A. baumannii* in hospitals on different continents is not currently available. Hence, this study aimed to comprehensively determine the prevalence of colistin resistance in *A. baumannii* obtained from clinical samples. Regional differences in these rates were also investigated using subgroup analyses.

## Material and method

### Search strategy and study selection

To find the relevant articles, we searched MEDLINE (PubMed), Embase, and Web of Science for relevant studies published in the English language from 2000 up to April 25, 2023. The following search syntax was utilized for search in PubMed and other databases. The comprehensive search was conducted using “*Acinetobacter baumannii*’’ OR “*Acinetobacter*”) AND (“antimicrobial-drug resistance” OR “colistin”) and all relevant keywords without any restriction during searching the databases. We used Mesh, EMtree and free text method to determine synonyms. This review was conducted and reported in accordance with the Preferred Reporting Items for Systematic Reviews and Meta-Analyses guidelines (PRISMA) [10]. The records found through database searching were merged, and the duplicates were removed by EndNote 20 (Thomson Reuters, New York, NY, USA). To prevent bias, two individual reviewers screened the records by title/abstract and full text to exclude irrelevant or duplicate articles. One of the researchers randomly evaluated the search results and confirmed that no pertaining studies had been ignored. The third author investigated any disparities.

### Selection criteria and data extraction

Two reviewers designed a data extraction form and extracted data from all eligible studies. All articles that reported the number of total clinical *A. baumannii* isolates and the number of colistin-resistance *A. baumannii* isolates were included in this meta-analysis.

The following data were extracted and sorted by: first author, antibiotic susceptibility test method and sample size for all of AST method; And first author, publication year, period of sample collection, study continent and country, guidelines, sample type, hospital ward of sample and sample size for studies that reported resistance to colistin based on broth microdilution method. (Additional file 1: Table S1). Studies were excluded if they met the following conditions: (1) *A. baumannii* which was isolated from environment; (2) colistin resistance was not presented or only reported as MIC50/90; (3) combined effects of antibiotics were reported; (4) The guideline used is not specified; (5) no clear reporting of resistance rates; (6) experimental studies on animal models; (7) Data were from conference abstracts, editorials, narrative reviews, systematic review and/or meta-analysis; (8) failure to access full articles even after establishing contact with the corresponding author via electronic mail.

### Quality assessment

The quality of the included studies was assessed by two blinded reviewers using an adapted version of the tool proposed by the Joanna Briggs Institute (JBI) Checklist adapted for prevalence studies [11] (Additional file 1: Table S1). A score ranging from 0 to 9 points was attributed to each study (8–9 points: high quality, 6–7 points: moderate quality, ≤ 5 points: low quality.

### Data analysis

Data Analysis of the global prevalence of colistin resistance among clinical isolates of *A. baumannii* was calculated in Stata software version 17. Subgroup analyses were done according to the period of sample collection, publication year, study continent and country, antimicrobial susceptibility method (AST), guidelines, sample type, hospital ward of sample, source of acquired infection, and quality score of studies that reported resistance to colistin based on broth microdilution method.

A random-effects model was applied to estimate the pooled prevalence of colistin resistance among clinical *A. baumannii* isolates at a 95% confidence interval (CI). Heterogeneity was checked using I^2^ test statistics. I^2^ ≤ 25% indicated low homogeneity, 25% < I^2^ ≤ 75% indicated moderate heterogeneity, and I^2^ > 75% indicated high heterogeneity. Funnel plot and Egger test were used to assess the existence of publication bias. The results were considered to have a publication bias at *P* < 0.05.

## Results

### Search results

**Fig. 1:**
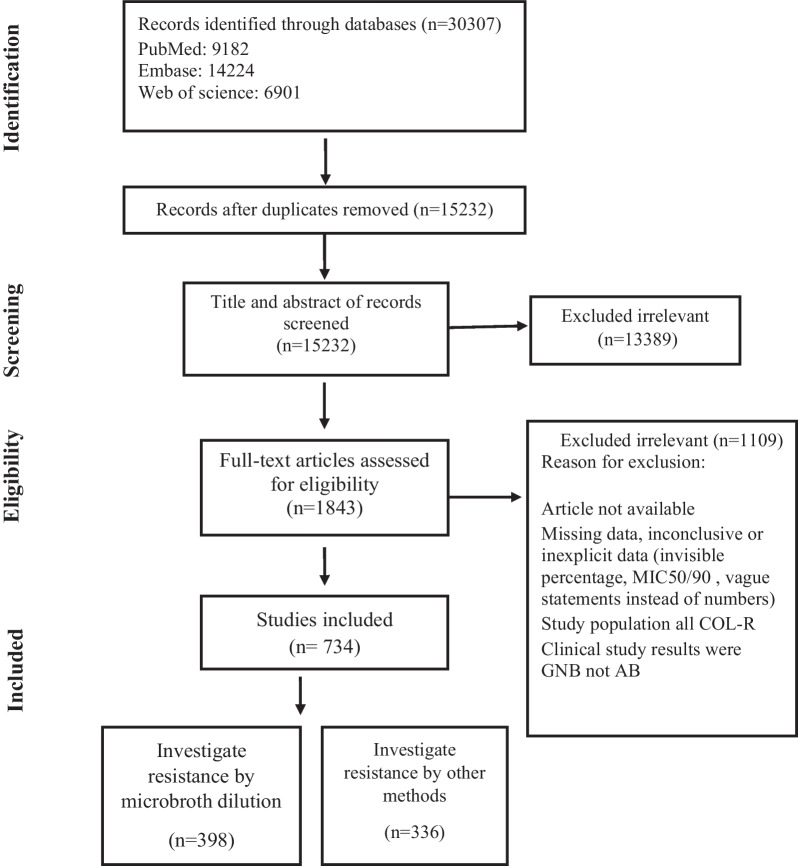
Flow chart of study selection for inclusion in the systematic review and meta-analysis

The process for the selection of articles is shown in Fig. 1. A total of 30307 articles were found in the initial search by database searching; after the removal of duplicates, the titles and abstracts of 15232 articles were screened. Finally, 734 studies were included in this systematic review and meta-analysis. Characteristics and references of included studies are presented in Additional file 1: Table S1.

**Fig. 2:**
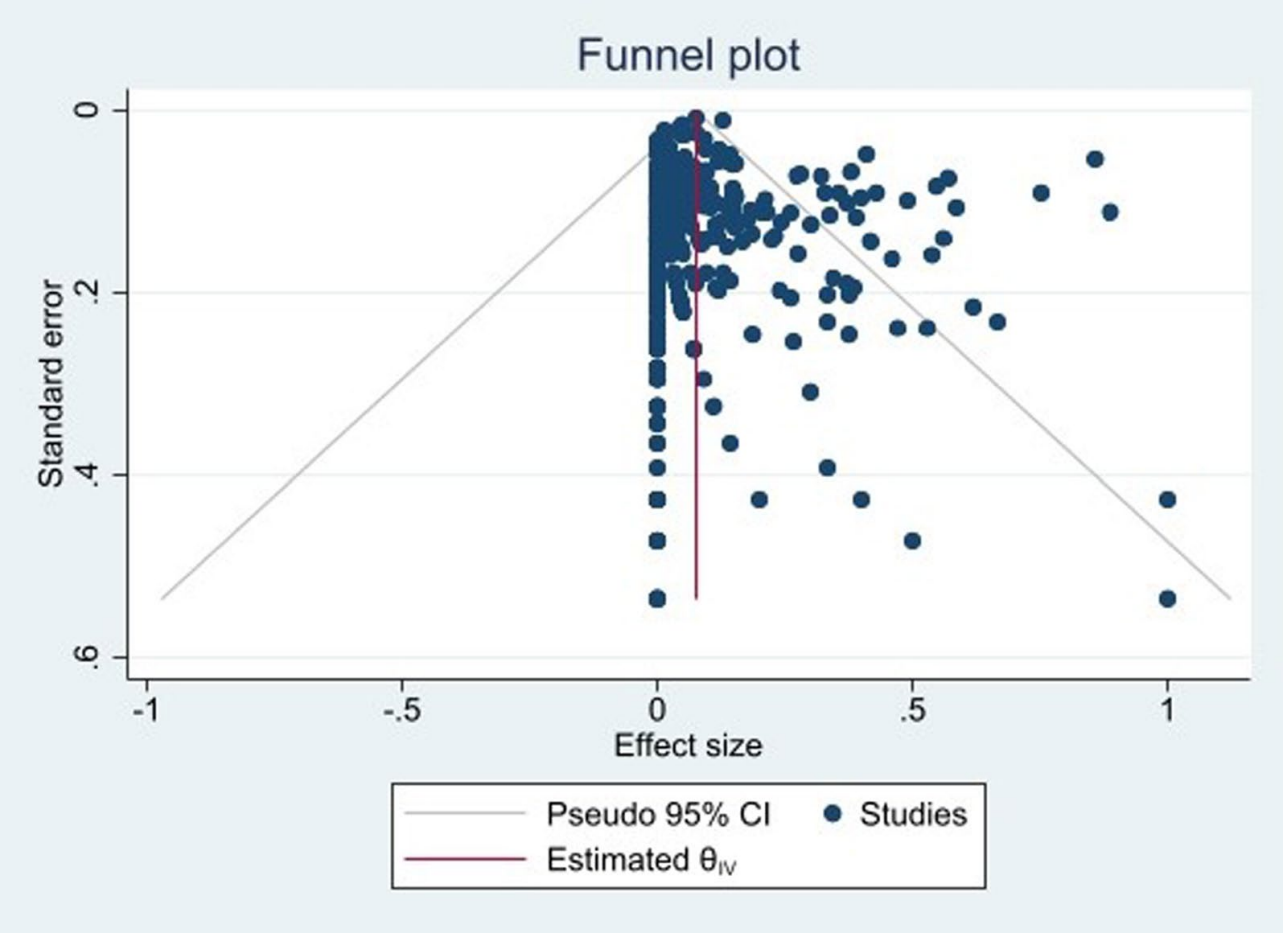
Funnel plot for meta-analysis

A total of 398 articles investigated colistin resistance in *A. baumannii* using the microbroth dilution method. The pooled prevalence of colistin resistance in clinical *A. baumannii* isolates based on the broth microdilution method was estimated at 4% (95% CI 3–5%; I^2^ = 95.31%; *P* < 0.001). The result of publication bias was shown in the funnel plot (Fig. 2); and Egger tests were also used to indicate the extent of publication bias (*P* = 0.483).

### Subgroup meta-analysis

The resistance rate to colistin and the subgroup analysis by continent, country, period of sample collection, year of publication, method of susceptibility testing, quality score, guideline, hospital ward, source of acquired infection, and sample are presented in Additional file 2: Table S2 and Figs. 3, 4, 5, 6, 7, 8, 9, 10, 11, 12.

**Fig. 3:**
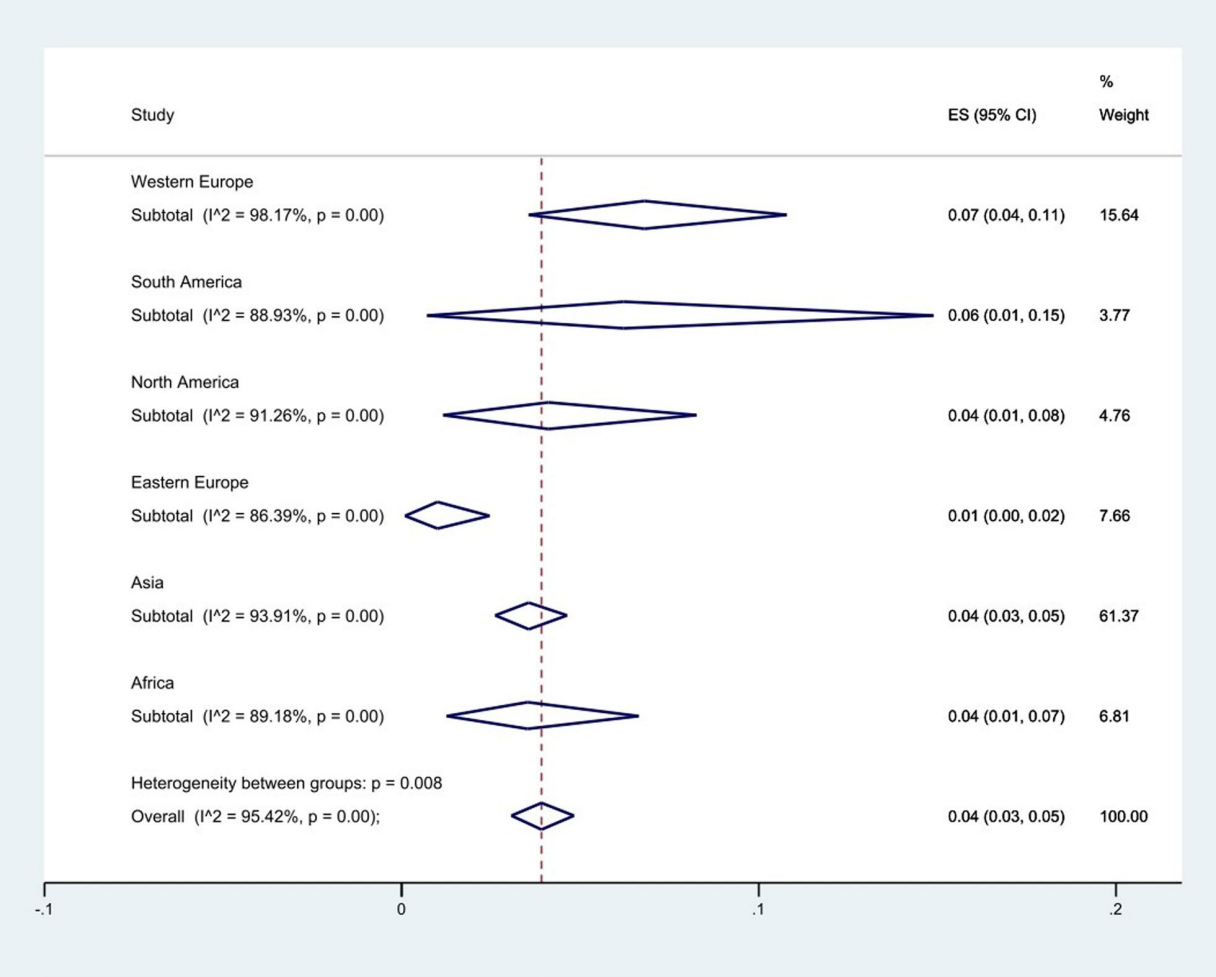
Subgroup meta-analysis for Continent

The subgroup meta-analysis of continents showed that western Europe had the highest resistance to colistin with 7% (95% CI 4–11) and eastern Europe had the lowest resistance to colistin with 1% (95% CI 0–2), (*P* = 0.008). Only one study was conducted in Oceania, which was not included in this subgroup analysis (Fig. 3).

**Fig. 4:**
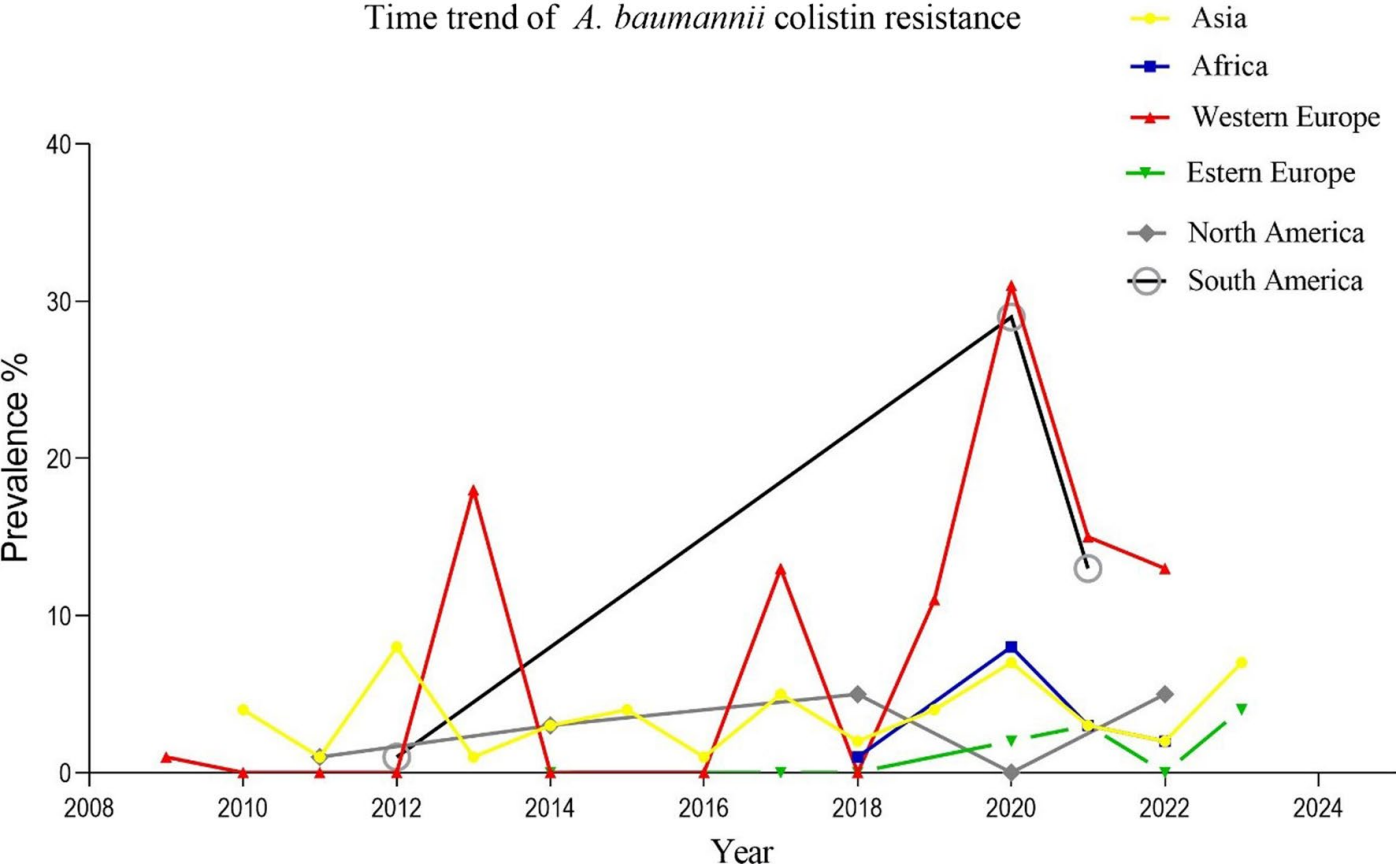
Time trend Meta-analysis, colistin resistance in *A. baumannii* clinical isolates among continent from 2000 to 2023

Figure 4 shows the time trend of *A. baumannii* colistin resistance prevalence by continent. In general, this analysis showed an increase in resistance in all continents over time.

**Fig. 5:**
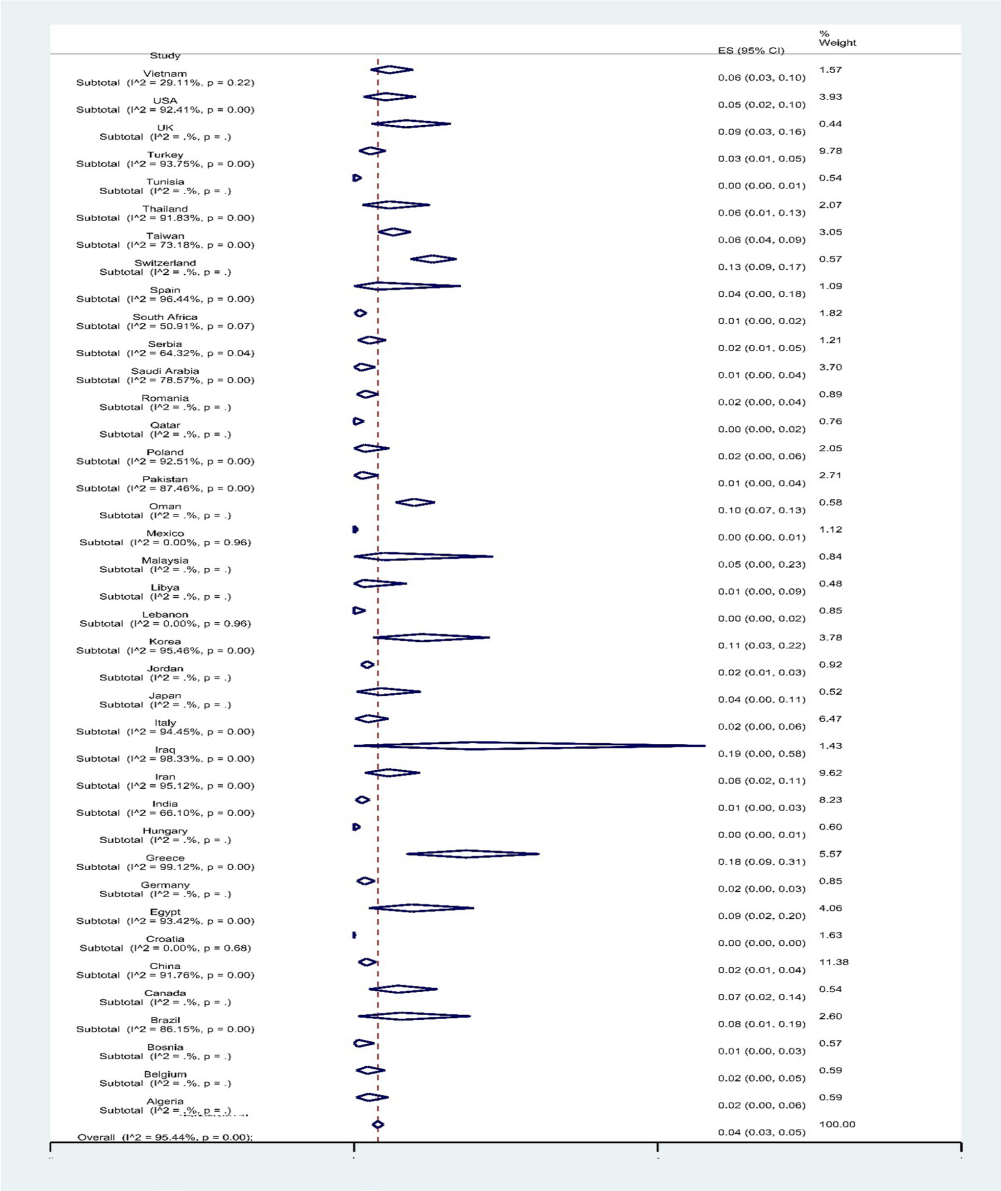
Subgroup meta-analysis for countries

Subgroup meta-analysis of countries showed that Iraq with 19% (95% CI 0–58%) and Greece with 18% (95% CI 9–31%) had the highest resistance to colistin (*P* < 0.001) (Fig. 5).

**Fig. 6:**
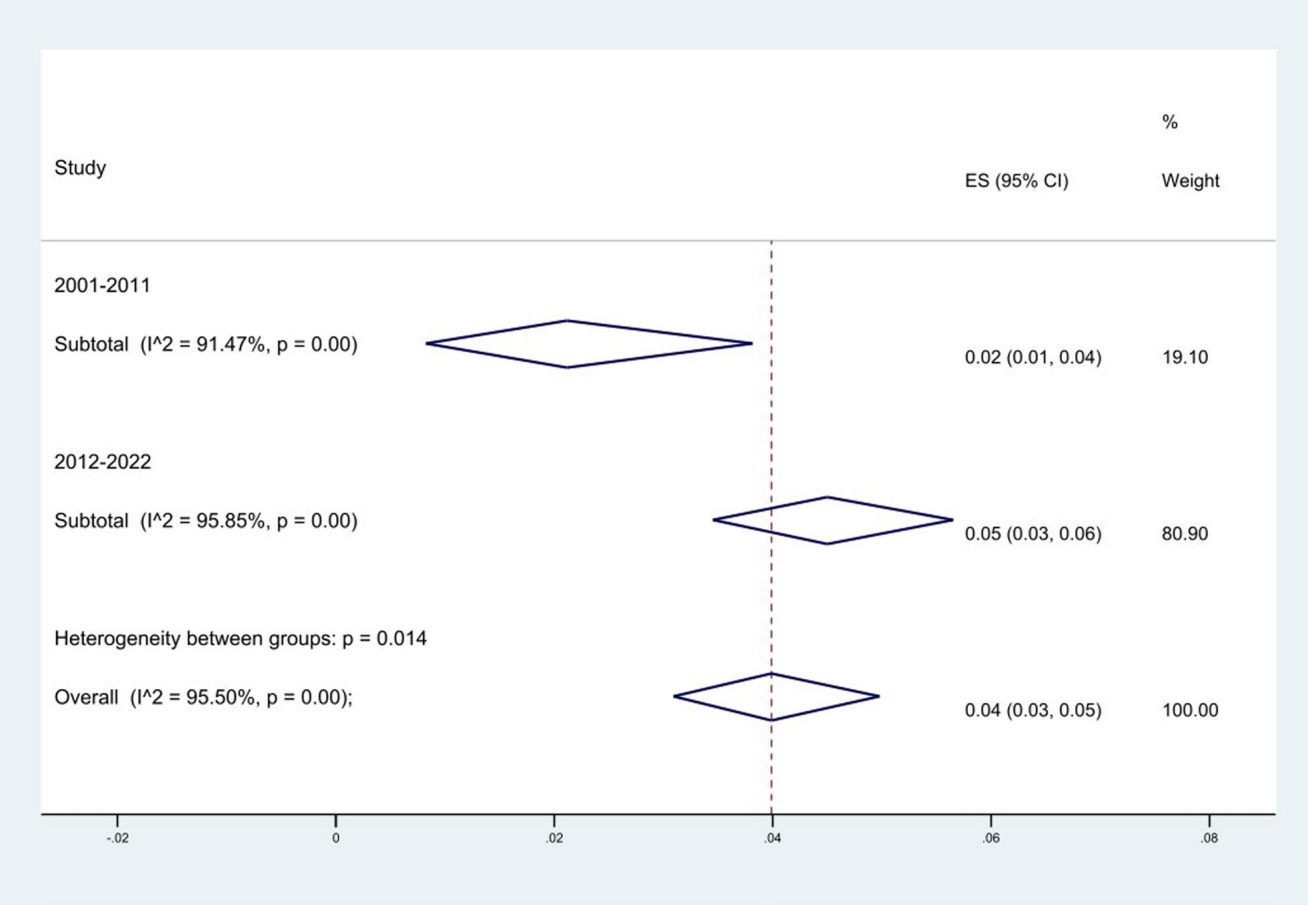
Subgroup meta-analysis for period of samples collection

We generally divided the sample collection time into 2 periods: 2001–2011, and 2012–2023. The results of the analysis showed an increase in resistance over time from 2% (95% CI 1–4%) to 5% (95% CI 3–6%) over time (*P* = 0.014) (Fig. 6).

**Fig. 7:**
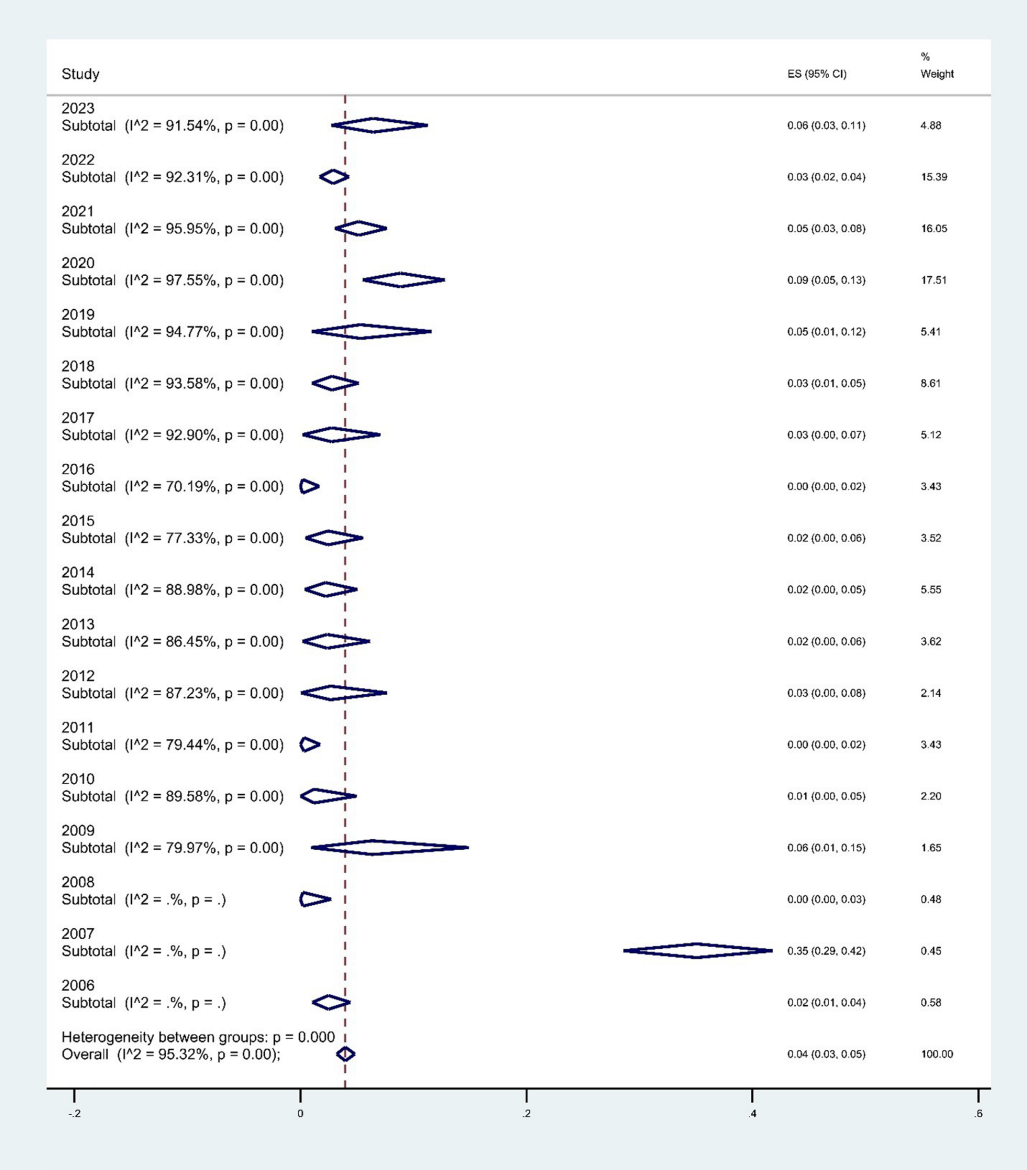
Subgroup meta-analysis for publication year of articles

The publication year subgroup analysis indicated an increase in colistin resistance in *A. baumannii* from 2% (95% CI 1–4%) in 2009 to 6% (95% CI 3–11%) in 2023 (*P* < 0.001), (Fig. 7).

**Fig. 8:**
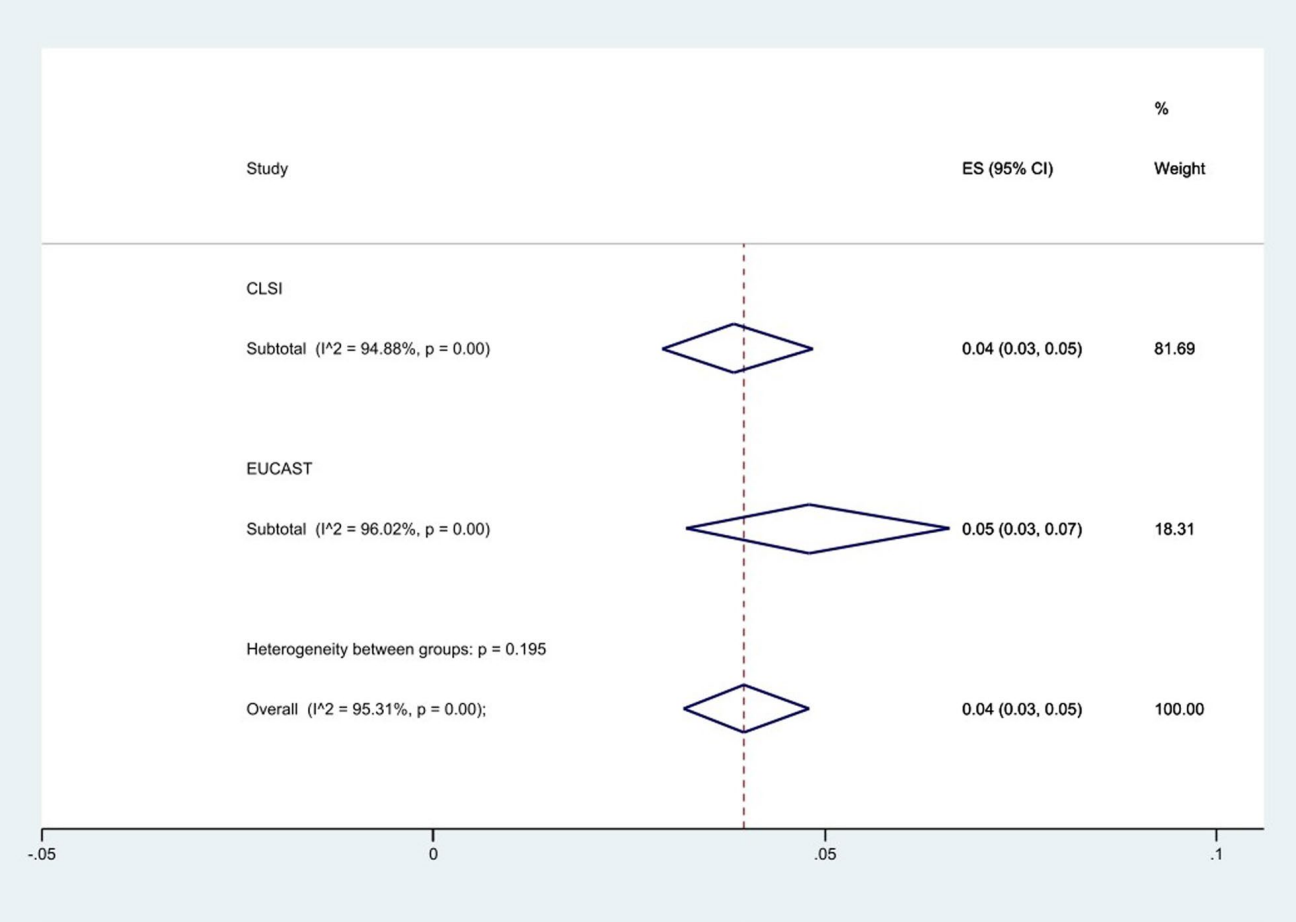
Subgroup meta-analysis for guidelines

Subgroup analysis based on guidelines showed that the group of EUCAST had a higher level of resistance 5% (95% CI 3–7%) rather than CLSI 4% (95% CI 3–5%), (*P* = 0.195), (Fig. 8).

**Fig. 9:**
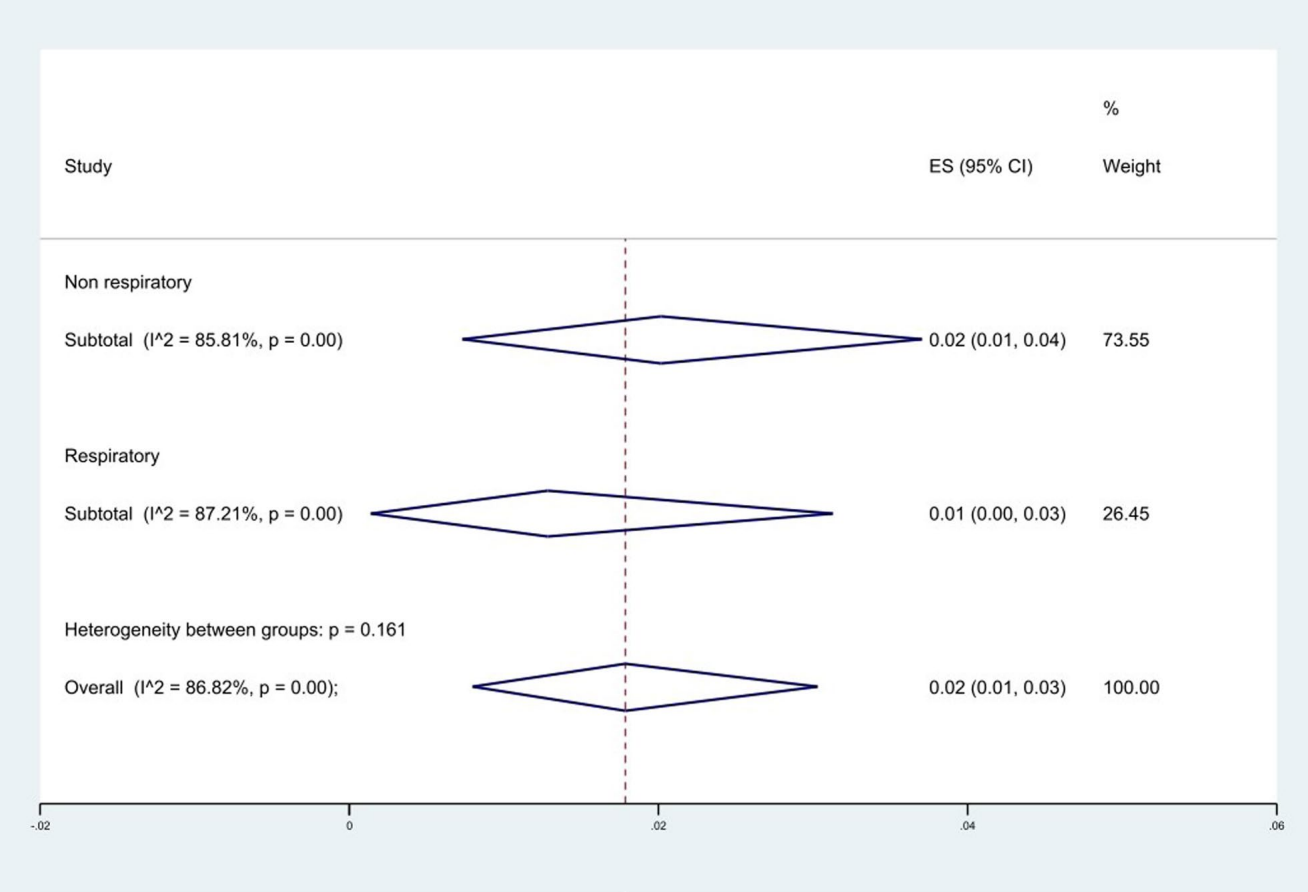
Subgroup meta-analysis for sample origin

**Fig. 10:**
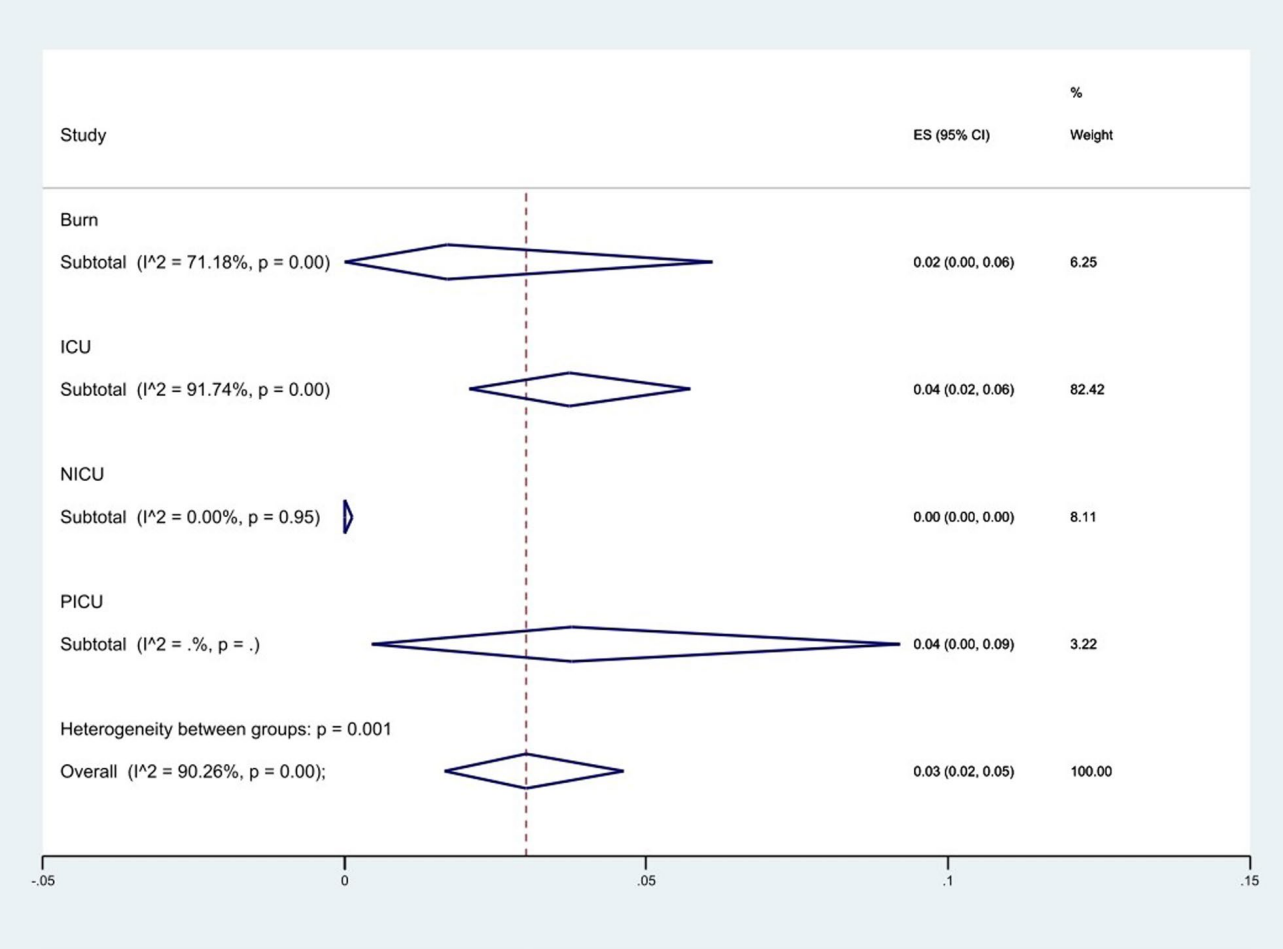
Subgroup meta-analysis for sample hospital wards

We classified the origin of the collected samples into respiratory and non-respiratory groups. The results of the analysis showed that the level of resistance to colistin in the non-respiratory group 2% (95% CI 1–4%) was higher than the respiratory group 1% (95% CI 0–5%), (*P* = 0.161), (Fig. 9), and among the hospital wards where the samples were collected, ICU 4% (95% CI 2–6%) and PICU 4% (95% CI 0–9%) had the highest resistance rate and NICU 0% (95% CI 0–0%) had the lowest rate (*P* = 0.001), (Fig. 10).

**Fig. 11:**
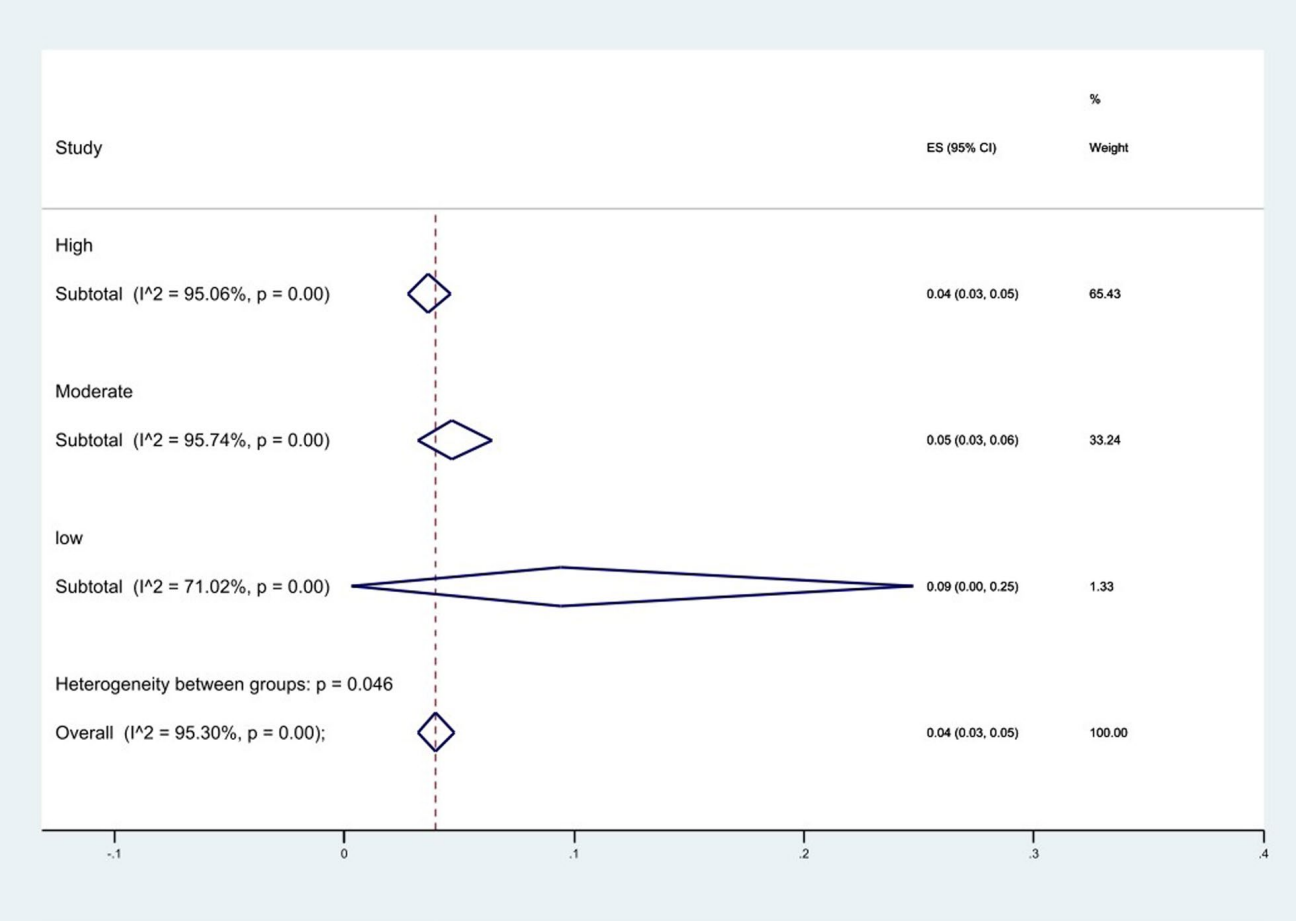
Subgroup meta-analysis based on quality of included studies

The results of the quality score analysis showed that the low quality 9% (95% CI 0–25%) had the highest level of resistance (*P* = 0.046), (Fig. 11).

**Fig. 12:**
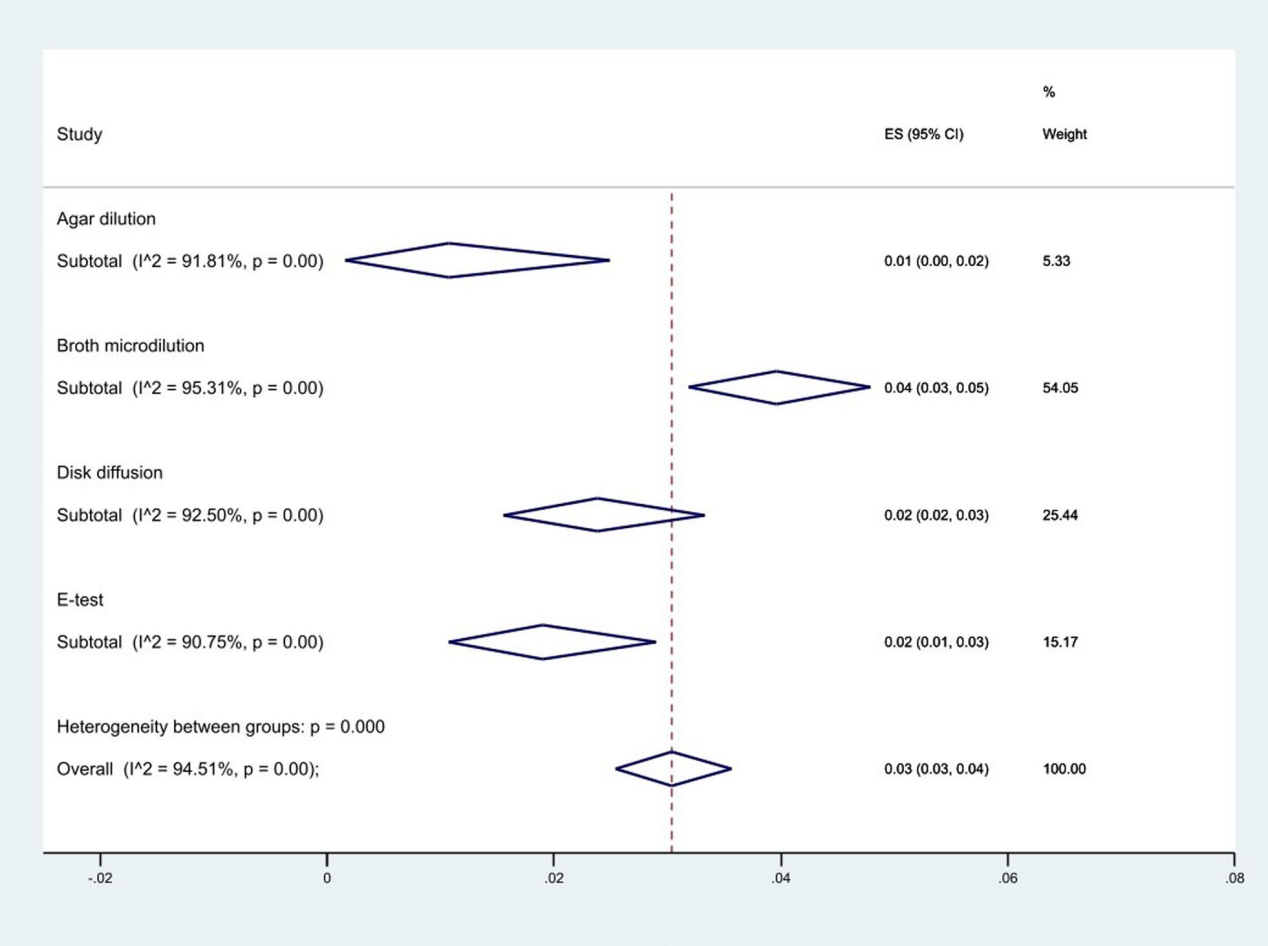
Subgroup meta-analysis for AST methods

We reviewed other studies that used various methods to measure resistance. 38 articles used the agar dilution method, 111 used the E-test method, 187 used the disk diffusion method, and 398 used the broth microdilution method. The results of the analysis showed that the broth microdilution method 4% (95% CI 3–5%) had the highest level of resistance, while the agar dilution 1% (95% CI 0–2%) showed the lowest level, (*P* < 0.001) (Fig. 12).

**Fig. 13:**
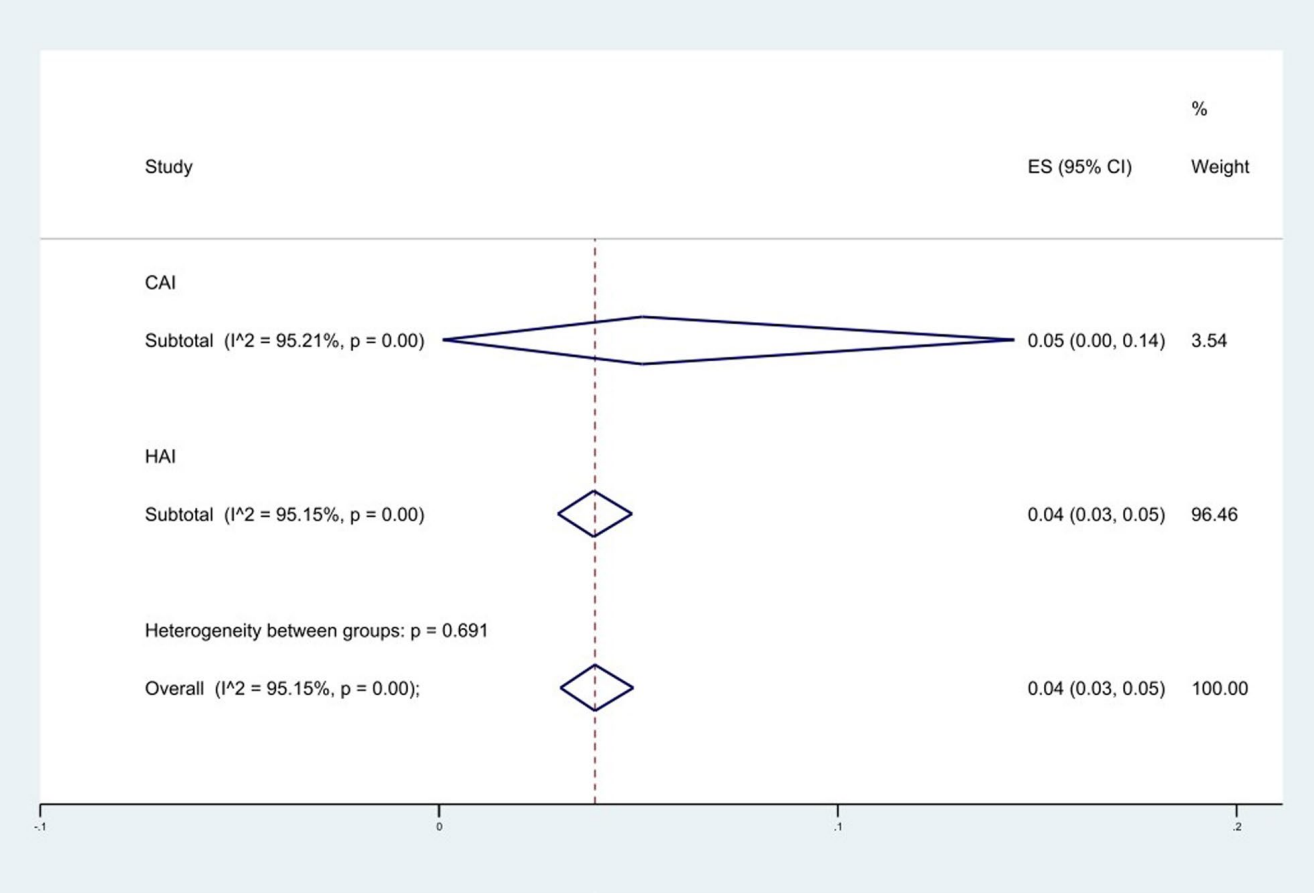
Subgroup meta-analysis for Hospital/community acquired infection

Subgroup meta-analysis based on the source of acquired infection revealed 5% (95% CI 0–14%) for the community acquired infections (CAIs) and 4%; (95% CI 3–5%) for the hospital acquired infections (HAIs) (*P* = 0. 691) (Fig. 13); however, only 3.63% of studies were performed on *A. baumannii* isolated from CAIs.

## Discussion

Colistin is one of the remaining therapeutic options for the management of MDR *A. baumannii* infections and has been utilized as a rescue therapy for severe infections. However, *A. baumannii* may be able to persist under the pressure of antibiotics due to the rapid emergence of colistin resistance from a heteroresistant population during treatment [12]. The emergence of colistin resistance is a significant threat to patient survival and requires increased attention from the medical community [13]. The LPS loss or its modification is more commonly associated with the resistance to colistin. Although these mechanisms allow the organism to acquire colistin resistance, but they might be less virulent. Carretero-Ledesma et al. [14] indicated phenotypic changes associated with colistin resistance due to loss of LPS. Moreover, they indicated that LPS-deficient *A. baumannii* had reduced survival and proliferation in a mouse model of disseminated sepsis. Also, TNF-a and IL-6 levels were undetectable in all mice infected with LPS-deficient *A. baumannii,* comparable to uninfected mice. LPS-deficient *A. baumannii* grew significantly slower than the parental strain and had decreased biofilm production due to LPS loss. LPS deficiency also caused increased sensitivity to chlorhexidine, deoxycholate, and sodium dodecyl sulfate. LPS-deficient *A. baumannii* showed reduced surface motility in vitro and reduced virulence and mortality in the mouse model, resulting in no deaths. Also, Farshadzadeh et al. showed a remarkable decrease of several pathobiological properties including surface attachment, surface motility, and in vitro and in vivo biofilm formation capacity of LPS-deficient *A. baumannii* isolates [15]. According to the findings of Kamoshida et al., loss of LPS in *A. baumannii* caused a weak stimulatory effect on neutrophils, resulting in decreased levels of reactive oxygen species (ROS) and inflammatory cytokine production. Nevertheless, neutrophils showed a preference for killing LPS-deficient *A. baumannii* strains over wild-type strains. In addition, LPS-deficient *A. baumannii* strains showed increased sensitivity to lysozyme and lactoferrin [16].

After an extensive literature search and a rigorous selection process, a total of 734 studies were considered eligible for inclusion. Of these, 398 studies evaluated the prevalence of colistin-resistant *A. baumannii* clinical isolates using broth microdilution. Also, 187, 111 and 38 studies indicated the prevalence rate of resistance to colistin using disk diffusion, E-test and agar dilution method, respectively, but their results were considered unreliable by CLSI due to insufficient diffusion of the large molecule of colistin on agar, resulting in smaller inhibition halo diameters, poor reproducibility, and numerous errors compared to the broth microdilution reference method [17]. From our meta-analysis findings, the overall resistance rate to colistin was (4%; 95% CI 3–5%). The prevalence of resistance to this drug has increased significantly (*P* > 0.01) regarding various time periods (2% from 2001 to 2011, and 5% from 2012 to 2023). Also, the number of published studies on colistin-resistant *A. baumannii* strains has increased from 43 articles published before 2012 to 354 articles published between 2012and 2023. A similar study showed a significant growth in colistin resistance publications over the last decade [18]. This growth suggests the spread of colistin resistance among *A. baumannii* strains in different settings, such as hospital wards, and highlights its global spread. Results from a surveillance network in the United States of America have also shown a significant increase in colistin resistance in *A. baumannii* in recent years, more than doubling from 2.8% in 2006–2008 to 6.9% in 2009–2012 [19]. Seifert et al., found the low rate of resistance to colistin (4.8%) between 2012 and 2016 in 313 carbapenem-resistant *A. baumannii* isolated from various body sites in patients from 114 hospitals in 47 countries and five world regions [20]. Also, according to data from a meta-analysis study, the low global prevalence of resistance to colistin was found in both Organization for Economic Co-operation and Development (OECD) countries and non-OECD countries (1.4% vs. 1.3%) during 2000 to 2016 [21]. On the other hand, results from a SENTRY antimicrobial surveillance program (1997–2016) showed a slow trend of increasing resistance to colistin among *A. baumannii* isolates from 2006 to 2016 [22].

In agreement with our findings, Pormohammad et al., [23] in a meta-analysis review showed the relatively similar prevalence of resistance to colistin, but the number of their studies was lower than ours (only 167) during a period from 2001 to 2017. In addition, 61 out of 167 studies measured resistance to colistin using the disc diffusion method, with a prevalence of 5%, which is an invalid method for assessing susceptibility to colistin. However, the resistance rates using the MIC/E-test (in 77 reports) and the Vitek-2 method (in 29 reports) were 3% and 2%, respectively.

On the other hand, the analysis of the resistance rate with respect to the year of publication shows an increase in resistance from 5 to 9% from 2019 to 2020, which could be due to the widespread use of antibiotics during the Corona pandemic, although after that, the level of resistance has decreased with the control of the disease. We can also observe this issue in the examination of the resistance of each continent during different years, for example, the amount of resistance in Western Europe was 11% in 2019, while it had increased to 31% in 2020.

This increase in resistance can be explained by the more widespread use of colistin in clinical practice, particularly in the veterinary field for the purposes of bacterial infection control and growth promotion. There has been some evidence of the emergence of resistance among bacteria and the spread of resistance from animals to humans [13]. Direct human-animal contact, bacterial spread via food ingestion, indirect spread via environmental emissions, and eventual human exposure via the environment have all been identified as important modes of transmission of pathogen resistance from animals to humans [6]. Thus, there is a major need for increased systematic surveillance of colistin-resistant bacteria (including *A. baumannii*) in food-producing animals to effectively control resistance to this antibiotic [24]. Previous colistin treatment is a primary risk factor for colistin-resistant *A. baumannii* colonization and infection.

Subgroup analyses showed a large difference in the rate of colistin resistance between different regions of the world. The highest rates of resistance were found in western Europe (7%, 61 reports) and South America (6%, 16 reports). However, most reports (234 reports, 60.94%) were from Asia with a prevalence of 4% (particularly from China, Turkey and Iran with 41, 36 and 35 reports and a resistance prevalence of 2%, 3% and 6%, respectively) rather than from other continents.

Among the European countries, Italy with 23 and Greece with 20 published the most articles on this topic, with a prevalence of colistin-resistant isolates of 2% and 18%, respectively. The USA (in 14 reports) and Brazil (in 11 reports) have the most studies in this area among American countries, with a prevalence of 5% and 8%, respectively. On the other hand, there is an alarm for the high prevalence of resistance rates in Israel (59%), France, the United Arab Emirates (50%), and Argentina (46%); however, due to the evaluation of *A. baumannii* antimicrobial susceptibility testing in one clinical center in these two countries, these data cannot be generalized to all parts of these regions in these countries. However, Pormohammad et al. [23] indicated the highest prevalence of colistin-resistant *A. baumannii* from Lebanon (17.5%) and China (12%).

Recently, in an effort to standardize colistin susceptibility testing, CLSI and EUCAST have established the colistin breakpoints. These efforts resulted in recommendations stating that the only valid diagnostic method for this purpose is the standard broth microdilution test without additives [25]. For this antibiotic, only the CLSI guideline breakpoint is available (MIC ≥ 4 mg/L as resistant), but some studies have interpreted their results using the EUCAST breakpoint of ≥ 2 mg/L suggested for *Enterobacteriaceae*, so a difference between the resistance rates, according to the breakpoint used, was also observed in our study. The prevalence of resistance was 6% (95% CI 3–7%) using EUCAST, but 4% (95% CI 3–5%) using CLSI. Unlike this data, Pormohammad et al. [23] did not perform the comparison of guidelines on colistin-resistant *A. baumannii* in their meta-analysis.

One of the main limitations of our study was the lack of evaluation of colistin heteroresistant isolates. It is important to note that the presence of colistin heteroresistance tends to evade detection by methods such as the Vitek 2 or E test, and may even evade detection by broth microdilution [26]. Therefore, there is a possibility that the true incidence of colistin resistance in vivo may be underestimated and its impact underestimated. On the other hand, the heterogeneity of the included articles in terms of countries, guidelines and time period was high. The high heterogeneity may be due to the inclusion of samples from different sources (e.g. blood culture, sputum, urine, wound) and also to the different levels of colistin use in treatment guidelines. In this meta-analysis, a few studies evaluated the frequency rates of ESBLs and mcr genes on all *A. baumannii* isolates and not on colistin-resistant isolates. In addition, they did not correlate the presence of ESBLs and *mcr* genes with colistin-resistant isolates. Therefore, we did not analyze these two factors.

## Conclusion

In conclusion, this meta-analysis found a low prevalence of colistin resistance among *A. baumannii* isolates responsible for infections worldwide from 2000 to 2023. However, there is a high prevalence of colistin-resistant isolates in certain countries. This implies an urgent public health threat, as colistin is one of the last antibiotics available for the treatment of infections caused by XDR strains of *A. baumannii*. The results of this study also highlighted the regional differences in the colistin antimicrobial susceptibility profile of *A. baumannii* associated with nosocomial infections worldwide, so that the resistance-increasing trends were observed in regions where polymyxins are heavily used, such as Israel, France, and the United Arab Emirates.

### Supplementary Information

**Additional file 1: Table S1.** Characteristics and references of included studies are presented

**Additional file 2: Table S2.** Subgroup meta-analysis

## Data Availability

Data supporting reported results will be available upon request for the peer-review process.

## Abbreviations

MDR: Multidrug-resistant
XDR: Extensively drug-resistant
pETN: Phosphoethanolamine
ABC: ATP-binding cassette
JBI: Joanna Briggs Institute
CI: Confidence interval
