# Supplementary material for: Prevalence of colistin resistance in clinical isolates of Acinetobacter baumannii: a systematic review and meta-analysis
Source: Antimicrob Resist Infect Control. 2024 Feb 28;13:24. doi: 10.1186/s13756-024-01376-7 (PMC10902961; doi:10.1186/s13756-024-01376-7)
Supplement: Supplementary file 1 — Additional file 1: Table S1. Characteristics and references of included studies are presented [file 13756_2024_1376_MOESM1_ESM.docx]

| Author name | Date | Date of study | Country | Continent | Ward | Sample type | N. of *A.baumannii* | N. of colistin resistant *A.baumannii* | Guideline | CAI/HAI | AST Method | Quality score |
| --- | --- | --- | --- | --- | --- | --- | --- | --- | --- | --- | --- | --- |
| A. Balkhair (1) | 2023 | 2017 to 2020 | Oman | Asia |  |  | 29 | 0 | CLSI | HAI | Broth microdilution | 8 |
| Abbas Maleki (2) | 2022 | 2010 to 2021 | Iran | Asia |  |  | 60 | 0 | CLSI | HAI | Broth microdilution | 9 |
| Abdikarim Hussein Mohamed (3) | 2022 | 2019 to 2021 | Somalia | Africa |  |  | 24 | 1 | CLSI | HAI | Broth microdilution | 8 |
| Abdulrahman S Bazaid (4) | 2022 | 2020 to 2021 | Saudi Arabia | Asia |  |  | 64 | 0 | CLSI | HAI | Broth microdilution | 9 |
| Abdulzahra (5) | 2018 | 2015 to 2015 | Egypt | Africa |  |  | 40 | 2 | CLSI | HAI | Broth microdilution | 6 |
| Abebe Mekuria Shenkutie (6) | 2020 |  | China | Asia | ICU |  | 104 | 22 | CLSI | HAI | Broth microdilution | 9 |
| Abolfazl Vahhabi (7) | 2021 | 2018 to 2019 | Iran | Asia |  |  | 112 | 0 | CLSI | HAI | Broth microdilution | 9 |
| Acar Kaya (8) | 2017 | 2009 to 2014 | Turkey | Asia |  |  | 50 | 0 | CLSI | CAI | Broth microdilution | 8 |
| Adam Valcek (9) | 2022 | 2014 to 2017 | Belgium | Europe |  |  | 49 | 11 | CLSI | CAI | Broth microdilution | 6 |
| Agarwal (10) | 2017 | 2015 to 2016 | India | Asia | ICU | Respiratory | 63 | 0 | CLSI | HAI | Broth microdilution | 8 |
| Agarwal (11) | 2018 | 2016 to 2017 | India | Asia | ICU | Respiratory | 83 | 2 | CLSI | HAI | Broth microdilution | 7 |
| Ahmad Farajzadeh Sheikh (12) | 2020 | 2018 to 2019 | Iran | Asia |  |  | 70 | 2 | CLSI | HAI | Broth microdilution | 9 |
| Ahmed Morad Asaad (13) | 2021 | 2018 to 2019 | Egypt | Africa |  |  | 94 | 35 | CLSI | HAI | Broth microdilution | 9 |
| Akin (14) | 2018 | 2014 to 2015 | Turkey | Asia | PICU |  | 60 | 2 | CLSI | HAI | Broth microdilution | 7 |
| Al Bshabshe (15) | 2016 | 2014 to 2015 | Saudi Arabia | Asia | ICU |  | 49 | 0 | CLSI | HAI | Broth microdilution | 8 |
| Al Samawi (16) | 2016 | 2012 to 2013 | Qatar | Asia |  |  | 137 | 2 | CLSI | HAI, CAI | Broth microdilution | 8 |
| Alaa Abouelfetouh (17) | 2020 | 2010 to 2015 | Egypt | Africa |  |  | 74 | 11 | CLSI | HAI | Broth microdilution | 7 |
| Alafate Ayibieke (18) | 2020 | 2016 to 2017 | Ghana | Africa |  |  | 24 | 8 | CLSI | HAI | Broth microdilution | 7 |
| Aljindan (19) | 2015 | 2014 to 2014 | Saudi Arabia | Asia | ICU | Non respiratory | 47 | 0 | CLSI | HAI | Broth microdilution | 7 |
| Aljindan (20) | 2018 | 2014 to 2014 | Saudi Arabia | Asia |  |  | 60 | 0 | CLSI | HAI | Broth microdilution | 9 |
| Altaf Bandy (21) | 2020 | 2019 to 2019 | Saudi Arabia | Asia |  | Non respiratory | 17 | 0 | CLSI | HAI | Broth microdilution | 9 |
| Altaf Bandy(22) | 2022 | 2019 to 2020 | Saudi Arabia | Asia |  | Non respiratory | 23 | 1 | CLSI | HAI | Broth microdilution | 8 |
| Altun (23) | 2013 | 2010 to 2012 | Turkey | Asia | ICU |  | 30 | 0 | CLSI | HAI | Broth microdilution | 6 |
| Alyona Lavrinenko (24) | 2021 | 2011 to 2019 | Kazakhstan | Asia |  |  | 224 | 0 | EUCAST | HAI | Broth microdilution | 9 |
| Amarela Lukić-Grlić (25) | 2019 | 2016 to 2018 | Croatia | Europe |  |  | 12 | 0 | CLSI | HAI | Broth microdilution | 7 |
| Anane Yaw Adjei (26) | 2021 | 2017 to 2018 | South Africa | Africa |  |  | 40 | 0 | CLSI | HAI | Broth microdilution | 8 |
| Aneta Guzek (27) | 2022 | 2007 to 2019 | Turkey | Asia | ICU | Non respiratory | 96 | 0 | CLSI | HAI | Broth microdilution | 9 |
| Angel Varghese (28) | 2020 | 2018 to 2019 | India | Asia |  | Non respiratory | 23 | 0 | CLSI | HAI | Broth microdilution | 8 |
| [Anitha Gunalan](https://pubmed.ncbi.nlm.nih.gov/?term=Gunalan%20A%5BAuthor%5D) (29) | 2021 |  | India | Asia |  |  | 25 | 6 | CLSI | HAI | Broth microdilution | 8 |
| Anna Szczypta (30) | 2021 | 2017 to 2018 | Poland | Europe |  | Non respiratory | 9 | 1 | EUCAST | HAI | Broth microdilution | 9 |
| Antoine G. Abou Fayad (31) | 2021 |  | Libya | Africa | ICU |  | 23 | 1 | CLSI | HAI | Broth microdilution | 8 |
| Armengol (32) | 2019 |  | Spain | Europe |  |  | 7 | 0 | EUCAST | HAI | Broth microdilution | 4 |
| Arzu KAYIŞ (33) | 2022 | 2014 to 2016 | Turkey | Asia | ICU |  | 70 | 3 | CLSI | HAI | Broth microdilution | 8 |
| Asaad (34) | 2013 | 2012 to 2013 | Saudi Arabia | Asia |  |  | 68 | 0 | CLSI | CAI | Broth microdilution | 9 |
| Athanassa (35) | 2012 |  | Greece | Europe | ICU |  | 11 | 0 | EUCAST | HAI | Broth microdilution | 8 |
| Aydin (36) | 2018 | 2014 to 2015 | Turkey | Asia |  |  | 437 | 9 | CLSI | HAI | Broth microdilution | 9 |
| Aylin Uskudar Guclu (37) | 2021 | 2016 to 2019 | Turkey | Asia |  |  | 7533 | 964 | EUCAST | HAI | Broth microdilution | 9 |
| AYMEN MABROUK (38) | 2020 | 2019 to 2019 | Tunisia | Africa | Burn |  | 21 | 0 | EUCAST | HAI, CAI | Broth microdilution | 9 |
| Bado (39) | 2018 | 2010 to 2011 | Uruguay | South America | ICU |  | 78 | 0 | EUCAST | HAI | Broth microdilution | 8 |
| Bagheri-Nesami (40) | 2017 | 2014 to 2015 | Iran | Asia | ICU | Respiratory | 27 | 10 | CLSI | HAI | Broth microdilution | 8 |
| Bahador (41) | 2018 | 2014 to 2014 | Iran | Asia | Burn | Non respiratory | 100 | 1 | CLSI | HAI | Broth microdilution | 8 |
| Bahador (42) | 2015 | 2012 to 2012 | Iran | Asia | Burn |  | 62 | 7 | CLSI | HAI | Broth microdilution | 8 |
| Bahador (43) | 2013 | 2012 to 2012 | Iran | Asia | ICU |  | 94 | 14 | CLSI | HAI | Broth microdilution | 8 |
| Bahare Salehi (44) | 2021 | 2016 to 2017 | Iran | Asia |  |  | 95 | 0 | CLSI | HAI | Broth microdilution | 9 |
| Balaram Khamari (45) | 2020 | 2015 to 2016 | India | Asia |  |  | 14 | 1 | CLSI | HAI | Broth microdilution | 7 |
| Ballouz (46) | 2017 | 2010 to 2015 | Lebanon | Asia |  | Non respiratory | 5 | 0 | CLSI | HAI | Broth microdilution | 8 |
| Bardbari (47) | 2018 |  | Iran | Asia |  |  | 5 | 0 | CLSI | HAI | Broth microdilution | 7 |
| Batarseh (48) | 2015 | 2013 to 2013 | Jordan | Asia |  |  | 116 | 2 | CLSI | HAI | Broth microdilution | 9 |
| BAYRAM (49) | 2012 | 2009 to 2011 | Turkey | Asia | Burn | Non respiratory | 43 | 0 | CLSI | CAI | Broth microdilution | 7 |
| Behnam Sobouti (50) | 2020 | 2016 to 2016 | Iran | Asia | Pediatric |  | 115 | 9 | CLSI | HAI | Broth microdilution | 8 |
| Bence Balázs (51) | 2022 | 2017 to 2019 | Hungary | Europe |  |  | 120 | 0 | EUCAST | HAI | Broth microdilution | 9 |
| Bernardetta Segatore (52) | 2022 | 2018 to 2021 | Italy | Europe |  |  | 141 | 77 | CLSI | HAI | Broth microdilution | 9 |
| Biagio Santella (53) | 2021 | 2015 to 2019 | Italy | Europe |  |  | 687 | 4 | EUCAST | HAI | Broth microdilution | 8 |
| Bin Liu (54) | 2014 | 2011 to 2013 | China | Asia |  |  | 40 | 0 | CLSI | CAI | Broth microdilution | 7 |
| Bogdan (55) | 2017 | 2007 to 2012 | Croatia | Europe |  |  | 68 | 0 | CLSI | HAI | Broth microdilution | 8 |
| Bojana Lukovic (56) | 2020 | 2018 to 2018 | Serbia | Europe |  |  | 237 | 10 | CLSI | HAI | Broth microdilution | 9 |
| Boral (57) | 2019 | 2012 to 2012 | Turkey | Asia |  |  | 160 | 2 | CLSI | HAI | Broth microdilution | 9 |
| Bozkurt-Guzel (58) | 2014 | 2010 to 2011 | Turkey | Asia |  | Non respiratory | 100 | 10 | CLSI | HAI | Broth microdilution | 7 |
| Branka Bedenić (59) | 2022 | 2017 to 2020 | Croatia | Europe | ICU | Non respiratory | 4 | 0 | EUCAST | HAI | Broth microdilution | 8 |
| Vesna Bratić (60) | 2021 | 2019 to 2019 | Croatia | Europe | ICU |  | 14 | 0 | CLSI | HAI | Broth microdilution | 8 |
| Capan Konca (61) | 2021 | 2015 to 2017 | Turkey | Asia | ICU |  | 46 | 1 | CLSI | HAI | Broth microdilution | 9 |
| Carlos Henrique Camargo (62) | 2020 |  | Brazil | South America |  |  | 79 | 17 | CLSI | HAI | Broth microdilution | 7 |
| Carolina Silva Nodari (63) | 2020 | 2012 to 2017 | Brazil | South America |  |  | 18 | 12 | Other | HAI | Broth microdilution | 7 |
| Carretto (64) | 2011 | 2007 to 2007 | Italy | Europe |  | Respiratory | 248 | 0 | CLSI | HAI | Broth microdilution | 7 |
| Ceren Ozkul (65) | 2021 | 2017 to 2019 | Turkey | Asia |  | Non respiratory | 44 | 6 | EUCAST | HAI | Broth microdilution | 8 |
| Chaari (66) | 2013 | 2010 to 2011 | Tunisia | Africa | ICU | Respiratory | 92 | 0 | CLSI | HAI | Broth microdilution | 9 |
| Christopher Longshaw (67) | 2020 | 2014 to 2016 |  | Europe |  |  | 236 | 15 | CLSI | HAI | Broth microdilution | 7 |
| Chun-Hsien Lin (68) | 2017 |  | China | Asia |  |  | 7 | 1 | CLSI | HAI | Broth microdilution | 6 |
| Coelho-Souza (69) | 2012 | 2001 to 2008 | Brazil | South America |  |  | 31 | 1 | CLSI | HAI | Broth microdilution | 6 |
| Congcong Liu (70) | 2022 | 2020 to 2020 | China | Asia | ICU |  | 256 | 1 | CLSI | HAI | Broth microdilution | 9 |
| Custovic (71) | 2014 | 2013 to 2013 | Bosnia | Europe |  |  | 54 | 0 | CLSI | HAI | Broth microdilution | 7 |
| D’Arezzo (72) | 2010 | 2005 to 2009 | Italy | Europe |  |  | 114 | 0 | CLSI | HAI | Broth microdilution | 7 |
| Dae Hun Kim (73) | 2017 | 2003 to 2014 | Korea | Asia |  |  | 130 | 8 | CLSI | HAI | Broth microdilution | 7 |
| Dafopoulou (74) | 2018 | 2010 to 2015 | Greece | Europe |  |  | 12646 | 959 | EUCAST | HAI | Broth microdilution | 7 |
| Dagmara Depka (75) | 2023 | 2017 to 2020 | Poland | Europe |  |  | 100 | 10 | EUCAST | HAI | Broth microdilution | 9 |
| Daniela Bandić-Pavlović (76) | 2020 | 2017 to 2018 | Croatia | Europe | ICU |  | 23 | 1 | EUCAST | HAI | Broth microdilution | 8 |
| De Vos (77) | 2016 | 2006 to 2011 | Belgium | Europe |  |  | 131 | 0 | CLSI | HAI | Broth microdilution | 9 |
| Débora Fiorentin Vandresen (78) | 2021 | 2017 to 2019 | Brazil | South America | ICU |  | 29 | 0 | CLSI | HAI | Broth microdilution | 8 |
| Deena Jalal (79) | 2021 | 2018 to 2018 | Egypt | Asia |  |  | 31 | 4 | CLSI | HAI | Broth microdilution | 8 |
| Despoina Gkentzi (80) | 2020 | 2019 to 2019 | Greece | Europe | NICU | Non respiratory | 8 | 0 | EUCAST | HAI | Broth microdilution | 6 |
| Di Domenico (81) | 2017 | 2013 to 2016 | Italy | Europe |  | Non respiratory | 12 | 0 | EUCAST | HAI | Broth microdilution | 8 |
| [Diaa Alrahmany](https://pubmed.ncbi.nlm.nih.gov/?term=Alrahmany%20D%5BAuthor%5D) (82) | 2021 | 2016 to 2017 | Oman | Asia |  |  | 320 | 38 | CLSI | HAI | Broth microdilution | 6 |
| Dias (83) | 2017 | 2013 to 2013 | Brazil | South America |  |  | 44 | 0 | CLSI | HAI | Broth microdilution | 8 |
| Dimitra Petropoulou (84) | 2021 | 2015 to 2015 | Greece | Europe |  |  | 190 | 61 | CLSI | HAI | Broth microdilution | 9 |
| [Ðorde Taušan](https://pubmed.ncbi.nlm.nih.gov/?term=Tau%C5%A1an+%C3%90&cauthor_id=36569168) (85) | 2022 | 2007 to 2019 | Serbia | Europe |  |  | 61 | 4 | EUCAST | HAI | Broth microdilution | 9 |
| Doymaz (86) | 2019 | 2012 to 2017 | Turkey | Asia |  |  | 31 | 3 | CLSI | CAI | Broth microdilution | 7 |
| Duarte (87) | 2016 | 2010 to 2012 | Portugal | Europe |  |  | 79 | 0 | CLSI | HAI | Broth microdilution | 7 |
| Dung Si Ho(88) | 2023 | 2019 to 2020 | Vietnam | Asia |  | Respiratory | 11 | 0 | EUCAST | HAI | Broth microdilution | 8 |
| Duong Thi Hong Diep (89) | 2023 | 2019 to 2020 | Vietnam | Asia |  |  | 84 | 5 | CLSI | HAI | Broth microdilution | 9 |
| Durdu (90) | 2018 | 2012 to 2015 | Turkey | Asia | ICU |  | 350 | 8 | CLSI | HAI | Broth microdilution | 9 |
| ECEM ÇAĞLAN (91) | 2019 | 2016 to 2017 | Turkey | Asia | ICU |  | 200 | 56 | EUCAST | HAI | Broth microdilution | 7 |
| Ehsan Sharifipour (92) | 2020 |  | Iran | Asia | ICU |  | 17 | 8 | CLSI | HAI | Broth microdilution | 7 |
| Elena Roxana Buzilă (93) | 2021 | 2008 to 2018 | Romania | Europe |  |  | 322 | 11 | Other | HAI | Broth microdilution | 9 |
| Elham Abbasi (94) | 2021 | 2016 to 2017 | Iran | Asia |  |  | 100 | 0 | CLSI | HAI | Broth microdilution | 9 |
| Elio Castagnola (95) | 2021 | 2015 to 2017 |  |  | ICU | Non respiratory | 28 | 1 | CLSI | HAI | Broth microdilution | 9 |
| El-Shazly (96) | 2015 | 2011 to 2011 | USA | North America |  |  | 21 | 0 | CLSI | HAI | Broth microdilution | 5 |
| Emilia Cercenado (97) | 2021 | 2014 to 1018 | Spain | Europe |  |  | 255 | 12 | EUCAST | HAI | Broth microdilution | 9 |
| Erica S. Shenoy (98) | 2020 |  | USA | North America | ICU |  | 4 | 0 | CLSI | HAI | Broth microdilution | 6 |
| Erlangga Yusuf (99) | 2022 | 2015 to 2017 | Romania | Europe |  |  | 104 | 1 | CLSI | HAI | Broth microdilution | 9 |
| Esposito (100) | 2014 | 2011 to 2012 | Italy | Europe |  |  | 314 | 3 | CLSI | HAI | Broth microdilution | 7 |
| Esposito (101) | 2015 | 2011 to 2013 | Italy | Europe |  |  | 53 | 2 | CLSI | HAI | Broth microdilution | 7 |
| Esra AKKAN KUZUCU (102) | 2022 |  | Turkey | Asia | ICU |  | 110 | 17 | EUCAST | HAI | Broth microdilution | 9 |
| Falagas (103) | 2018 | 2015 to 2017 | Greece | Europe |  |  | 121 | 52 | CLSI | HAI | Broth microdilution | 7 |
| Fatemeh Bakhshi (104) | 2022 | 2020 to 2021 | Iran | Asia | Burn |  | 85 | 0 | CLSI | HAI | Broth microdilution | 9 |
| Fatima Sana (105) | 2021 | 2017 to 2019 | Pakistan | Asia | ICU |  | 310 | 3 | EUCAST | HAI | Broth microdilution | 7 |
| Fauzia Jabeen (106) | 2021 | 2020 to 2020 | Pakistan | Asia |  | Non respiratory | 101 | 0 | CLSI | HAI | Broth microdilution | 9 |
| Federica Sacco (107) | 2021 | 2017 to 2017 | Italy | Europe | ICU |  | 51 | 6 | EUCAST | HAI | Broth microdilution | 9 |
| Fereshteh Ezadi (108) | 2019 | 2016 to 2017 | Iran | Asia |  |  | 71 | 4 | CLSI | HAI | Broth microdilution | 8 |
| Fernando Pasteran (109) | 2021 | 2012 to 2017 | Argentina | South America |  |  | 37 | 17 | EUCAST | HAI | Broth microdilution | 7 |
| Fertzakis (110) | 2019 | 2017 to 2018 | Greece | Europe |  |  | 424 | 174 | CLSI | HAI | Broth microdilution | 9 |
| Flamm (111) | 2015 | 2012 to 2012 | USA | North America |  |  | 202 | 6 | EUCAST | HAI | Broth microdilution | 8 |
| Flamm (112) | 2014 |  | USA | North America |  |  | 202 | 6 | CLSI | HAI | Broth microdilution | 8 |
| Flávia Allegretti Alvares (113) | 2021 | 2018 to 2019 | Brazil | South America |  |  | 16 | 0 | Other | HAI | Broth microdilution | 8 |
| Fu (114) | 2010 | 2005 to 2005 | China | Asia |  |  | 226 | 22 | CLSI | HAI | Broth microdilution | 8 |
| Gabriele Bianco (115) | 2022 | 2019 to 2021 | Italy | Europe |  | Non respiratory | 70 | 0 | CLSI | HAI | Broth microdilution | 9 |
| Gamal Wareth (116) | 2021 |  | Vietnam | Asia |  |  | 11 | 1 | CLSI | HAI | Broth microdilution | 9 |
| Gazel (117) | 2018 | 2010 to 2012 | Turkey | Asia | ICU | Non respiratory | 31 | 0 | CLSI | HAI | Broth microdilution | 6 |
| Georgios Papathanakos (118) | 2020 | 2017 to 2019 | Greece | Europe | ICU | Non respiratory | 39 | 21 | EUCAST | HAI | Broth microdilution | 9 |
| Ghaith (119) | 2017 | 2015 to 2015 | Egypt | Africa | ICU |  | 50 | 0 | CLSI | HAI | Broth microdilution | 7 |
| Gholami (120) | 2018 | 2016 to 2017 | Iran | Asia |  |  | 110 | 0 | CLSI | HAI | Broth microdilution | 8 |
| Giamarellos-Bourboulis (121) | 2001 | 1995 to 2000 | Greece | Europe |  |  | 39 | 0 | CLSI | HAI | Broth microdilution | 7 |
| Giannouli (122) | 2011 | 2006 to 2010 | Italy | Europe |  |  | 57 | 0 | CLSI | HAI | Broth microdilution | 7 |
| Giorgia Montrucchio (123) | 2022 | 2021 to 2021 | Italy | Europe | ICU |  | 176 | 14 | EUCAST | HAI | Broth microdilution | 9 |
| Goudarzi (124) | 2016 | 2015 to 2015 | Iran | Asia | ICU |  | 120 | 0 | CLSI | HAI | Broth microdilution | 9 |
| Guzek (125) | 2017 | 2005 to 2015 | Poland | Europe | ICU | Respiratory | 764 | 0 | EUCAST | HAI | Broth microdilution | 8 |
| Guzek (126) | 2014 | 2010 to 2013 | Poland | Europe |  | Respiratory | 418 | 0 | CLSI | HAI | Broth microdilution | 8 |
| Hackel (127) | 2018 | 2014 to 2016 | USA | North America |  |  | 368 | 20 | CLSI | HAI | Broth microdilution | 5 |
| Hadas Kon (128) | 2020 | 2013 to 2017 | Israel | Asia |  |  | 87 | 51 | CLSI | HAI | Broth microdilution | 8 |
| Hakyemez (129) | 2013 | 2009 to 2011 | Turkey | Asia |  |  | 32 | 0 | CLSI | HAI | Broth microdilution | 8 |
| Harald Seifert (130) | 2019 | 2005 to 2016 |  |  |  |  | 323 | 44 | CLSI | HAI | Broth microdilution | 6 |
| Harald Seifert (131) | 2022 | 2016 to 2018 |  |  |  |  | 2534 | 114 | EUCAST | HAI | Broth microdilution | 9 |
| Hasan Ejaz (132) | 2022 | 2020 to 2021 | Pakistan | Asia |  |  | 200 | 0 | CLSI | HAI | Broth microdilution | 8 |
| Hasan Ejaz (133) | 2021 | 2020 to 2020 | Saudi Arabia | Asia |  |  | 174 | 0 | CLSI | HAI | Broth microdilution | 9 |
| Hongbin Chen (134) | 2015 | 2012 to 2012 | China | Asia |  |  | 100 | 0 | CLSI | CAI | Broth microdilution | 8 |
| Hu¨seyin B. € Ozcelik (135) | 2020 | 2017 to 2018 | Turkey | Asia | ICU |  | 50 | 0 | EUCAST | HAI | Broth microdilution | 7 |
| Hui Zhang (136) | 2020 | 2015 to 2017 | China | Asia |  |  | 1,360 | 73 | CLSI | HAI | Broth microdilution | 7 |
| Hui Zhang (137) | 2021 | 2015 to 2018 | China | Asia |  |  | 1889 | 75 | CLSI | HAI | Broth microdilution | 9 |
| Hyo-Ju Son (138) | 2020 | 2012 to 2018 | Korea | Asia |  | Non respiratory | 164 | 12 | CLSI | HAI | Broth microdilution | 8 |
| Hyun Ah Kim (139) | 2015 | 2011 to 2011 | Korea | Asia |  | Respiratory | 28 | 4 | CLSI | HAI | Broth microdilution | 8 |
| Hyun Mi Kang (140) | 2023 | 2001 to 2020 | Korea | Asia |  |  | 108 | 43 | CLSI | HAI | Broth microdilution | 9 |
| Ian Morrissey (141) | 2020 | 2013 to 2017 |  |  |  |  | 1502 | 112 | EUCAST | CAI | Broth microdilution | 7 |
| INA GAJIC (142) | 2020 | 2016 to 2017 | Serbia | Europe | ICU |  | 332 | 6 | CLSI | HAI | Broth microdilution | 9 |
| Ipek (143) | 2017 | 2012 to 2015 | Turkey | Asia |  |  | 34 | 0 | CLSI | HAI | Broth microdilution | 8 |
| İpek Mumcuoğlu (144) | 2022 | 2020 to 2021 | Turkey | Asia | ICU |  | 603 | 4 | EUCAST | HAI | Broth microdilution | 9 |
| Irina Gheorghe (145) | 2021 | 2017 to 2018 | Romania | Europe | ICU |  | 33 | 0 | CLSI | HAI | Broth microdilution | 8 |
| Israa M.S. Al-Kadmy (146) | 2020 | 2016 to 2018 | Iraq | Asia |  |  | 121 | 91 | CLSI | HAI | Broth microdilution | 7 |
| Jacinda C. Abdul-Mutakabbir (147) | 2021 |  | USA | North America |  | Non respiratory | 50 | 28 | CLSI | HAI | Broth microdilution | 8 |
| Jain (148) | 2019 | 2016 to 2017 | India | Asia |  | Respiratory | 28 | 0 | CLSI | HAI | Broth microdilution | 7 |
| James A. Karlowsky (149) | 2022 | 2016 to 2021 |  |  |  |  | 4,038 | 198 | CLSI | HAI | Broth microdilution | 8 |
| Jan Hrbacek (150) | 2021 | 2011 to 2019 | Czech Republic | Europe |  |  | 74 | 25 | EUCAST | HAI | Broth microdilution | 8 |
| Jantana Houngsaitong (151) | 2020 | 2017 to 2017 | Thailand | Asia |  |  | 412 | 58 | CLSI | HAI | Broth microdilution | 8 |
| Jetsi Mancilla-Rojano (152) | 2020 | 2015 to 2018 | Mexico | North America |  |  | 79 | 0 | CLSI | HAI | Broth microdilution | 9 |
| Jia Jie Woon (153) | 2021 | 2015 to 2016 | Malaysia | Asia | ICU |  | 100 | 0 | CLSI | HAI | Broth microdilution | 9 |
| Jin’e Lei (154) | 2016 | 2013 to 2013 | China | Asia | ICU |  | 11 | 0 | CLSI | HAI | Broth microdilution | 7 |
| Jingyuan Xi(155) | 2022 | 2019 to 2021 | China | Asia |  |  | 82 | 4 | CLSI | HAI | Broth microdilution | 8 |
| Ji-Young Choi (156) | 2014 | 2007 to 2013 | Korea | Asia |  |  | 72 | 28 | CLSI | HAI | Broth microdilution | 7 |
| Jones (157) | 2013 | 2011 to 2011 | China | Asia |  |  | 174 | 0 | CLSI | HAI | Broth microdilution | 7 |
| Jun Li (158) | 2020 | 2017 to 2019 | China | Asia |  | Non respiratory | 109 | 0 | CLSI | HAI | Broth microdilution | 9 |
| Junyan Qu (159) | 2020 | 2018 to 2018 | China | Asia |  |  | 89 | 8 | CLSI | CAI | Broth microdilution | 8 |
| Kai-Chih Chang (160) | 2011 | 2007 to 2007 | Taiwan | Asia |  |  | 134 | 14 | CLSI | HAI | Broth microdilution | 7 |
| Kais Kassim Ghaima (161) | 2016 | 2015 to 2015 | Iraq | Asia |  |  | 96 | 7 | CLSI | HAI | Broth microdilution | 6 |
| Kaliterna (162) | 2015 | 2009 to 2010 | Croatia | Europe |  |  | 109 | 0 | EUCAST | HAI | Broth microdilution | 8 |
| KAMAL ISMAIL BAKR AL OTRAQCHI (163) | 2022 | 2020 to 2021 | Iraq | Asia | ICU |  | 39 | 1 | CLSI | HAI | Broth microdilution | 8 |
| Kandelaki (164) | 2011 | 2007 to 2010 | Georgia | Asia |  |  | 80 | 0 | CLSI | HAI | Broth microdilution | 7 |
| Kansakar (165) | 2011 | 2005 to 2006 | Thailand | Asia |  |  | 84 | 0 | CLSI | HAI | Broth microdilution | 8 |
| Kara (166) | 2018 | 2016 to 2016 | Turkey | Asia |  | Non respiratory | 40 | 11 | CLSI | HAI | Broth microdilution | 4 |
| KARAGÖZ (167) | 2014 | 2012 to 2012 | Turkey | Asia |  | Non respiratory | 47 | 0 | CLSI | HAI | Broth microdilution | 7 |
| Karampatakis (168) | 2017 | 2012 to 2013 | Greece | Europe |  |  | 9 | 0 | CLSI | HAI | Broth microdilution | 7 |
| Kashif Hussain (169) | 2020 | 2010 to 2018 | China | Asia | NICU |  | 32 | 0 | CLSI | HAI | Broth microdilution | 8 |
| Katarina Novović (170) | 2023 | 2020 to 2021 | Serbia | Europe | ICU |  | 64 | 0 | EUCAST | HAI | Broth microdilution | 9 |
| Katchanov (171) | 2018 | 2015 to 2016 | Germany | Europe |  |  | 16 | 0 | EUCAST | HAI | Broth microdilution | 7 |
| Khin Thet Thet (172) | 2020 | 2016 to 2016 |  |  |  |  | 75 | 0 | CLSI | HAI | Broth microdilution | 7 |
| Slimene, Khouloud (173) | 2023 | 2021 to 2021 | Libya | Africa |  |  | 21 | 0 | CLSI | HAI | Broth microdilution | 8 |
| KIRKGÖZ (174) | 2014 | 2010 to 2011 | Turkey | Asia | ICU |  | 92 | 1 | CLSI | HAI | Broth microdilution | 7 |
| Kishii (175) | 2014 | 2003 to 2011 | Japan | Asia |  | Non respiratory | 22 | 0 | CLSI | HAI | Broth microdilution | 6 |
| Koca (176) | 2018 | 2017 to 2018 | Turkey | Asia | ICU |  | 37 | 0 | EUCAST | HAI | Broth microdilution | 7 |
| Kolpa (177) | 2018 | 2007 to 2016 | Poland | Europe |  |  | 120 | 1 | EUCAST | HAI | Broth microdilution | 9 |
| Konstantina Nafplioti (178) | 2020 | 2015 to 2016 | Greece | Europe |  |  | 347 | 298 | CLSI | HAI | Broth microdilution | 7 |
| Konstantinos Mantzarlis (179) | 2020 | 2013 to 2016 | Greece | Europe | ICU |  | 77 | 20 | CLSI | HAI | Broth microdilution | 9 |
| Kumar (180) | 2013 | 2010 to 2012 | India | Asia | NICU | Non respiratory | 65 | 0 | CLSI | HAI | Broth microdilution | 9 |
| Kumar (181) | 2018 | 2013 to 2015 | India | Asia |  | Respiratory | 85 | 0 | CLSI | HAI | Broth microdilution | 9 |
| Kyong Ran Peck (182) | 2012 | 2011 to 2011 | Korea | Asia |  | Non respiratory | 6 | 2 | CLSI | HAI | Broth microdilution | 6 |
| Lai (183) | 2019 | 2016 to 2016 | Taiwan | Asia | ICU |  | 138 | 14 | CLSI | HAI | Broth microdilution | 8 |
| Langamba Angom Longjam (184) | 2022 | 2018 to 2019 | India | Asia |  |  | 115 | 0 | CLSI | HAI | Broth microdilution | 9 |
| Lauro Vieira Perdigão Neto (185) | 2020 | 2011 to 2016 | Brazil | South America |  |  | 14 | 1 | CLSI | HAI | Broth microdilution | 6 |
| Leena Al-Hassan (186) | 2021 | 2017 to 2018 | Sudan | Africa |  |  | 71 | 0 | EUCAST | HAI | Broth microdilution | 9 |
| Leonard R. Duncan (187) | 2022 | 2017 to 2020 | USA | North America |  |  | 264 | 6 | CLSI | HAI | Broth microdilution | 9 |
| Letícia Dias de Melo Carrasco (188) | 2021 |  | Brazil | South America |  |  | 16 | 6 | EUCAST | HAI | Broth microdilution | 9 |
| Lihua Hu (189) | 2020 | 2014 to 2018 | China | Asia |  |  | 60 | 2 | CLSI | HAI | Broth microdilution | 9 |
| Lin (190) | 2016 | 2015 to 2015 | China | Asia |  |  | 222 | 0 | CLSI | HAI | Broth microdilution | 8 |
| Liqiong Chen (191) | 2021 |  | China | Asia |  |  | 5 | 5 | CLSI | HAI | Broth microdilution | 8 |
| Lowe (192) | 2018 | 2013 to 2014 | South Africa | Africa |  |  | 150 | 2 | EUCAST | HAI | Broth microdilution | 9 |
| Lucia Graña-Miraglia (193) | 2020 | 2011 to 2016 | Mexico | North America |  |  | 38 | 0 | CLSI | HAI | Broth microdilution | 7 |
| Mahmood Khan (194) | 2018 | 2016 to 2017 | India | Asia |  |  | 70 | 0 | CLSI | HAI | Broth microdilution | 9 |
| Mahmoud A. F. Khalil (195) | 2021 | 2019 to 2021 | Egypt | Africa | ICU |  | 54 | 0 | CLSI | HAI | Broth microdilution | 9 |
| Mahnaz Nikibakhsh (196) | 2021 | 2019 to 2020 | Iran | Asia |  |  | 106 | 0 | CLSI | HAI | Broth microdilution | 9 |
| Mai M. Zafer (197) | 2021 | 2020 to 2020 | Egypt | Africa |  |  | 20 | 1 | CLSI | HAI | Broth microdilution | 9 |
| Maleeha Urooj (198) | 2022 | 2020 to 2020 | Pakistan | Asia |  |  | 82 | 15 | EUCAST | HAI | Broth microdilution | 9 |
| Sengupta, Mallika (199) | 2022 | 2015 to 2016 | India | Asia | ICU |  | 30 | 0 | CLSI | HAI | Broth microdilution | 8 |
| Mamta Jajoo (200) | 2018 | 2011 to 2015 | India | Asia |  | Non respiratory | 46 | 1 | CLSI | HAI | Broth microdilution | 7 |
| Manohar (201) | 2017 | 2014 to 2015 | India | Asia |  |  | 5 | 2 | CLSI | HAI | Broth microdilution | 6 |
| Marina Gysin (202) | 2022 |  |  | Asia | ICU | Non respiratory | 30 | 1 | CLSI | HAI | Broth microdilution | 4 |
| Marisa Castro Jara (203) | 2021 | 2018 to 2019 | Brazil | South America |  |  | 46 | 4 | CLSI | HAI | Broth microdilution | 8 |
| Markogiannakis (204) | 2009 | 2002 to 2004 | Greece | Europe |  |  | 22 | 0 | CLSI | CAI | Broth microdilution | 8 |
| Marwan Osman (205) | 2020 | 2016 to 2017 | Lebanon | Asia | ICU |  | 20 | 0 | EUCAST | HAI | Broth microdilution | 7 |
| Maryam Seyyedi (206) | 2020 |  | Iran | Asia | ICU |  | 48 | 20 | CLSI | HAI | Broth microdilution | 9 |
| Matthew E. Falagas (207) | 2018 | 2015 to 2017 | Greece | Europe |  |  | 121 | 43 | CLSI | HAI | Broth microdilution | 7 |
| Matthew Gavino Donadu (208) | 2021 |  | Hungary | Europe |  |  | 62 | 0 | EUCAST | HAI | Broth microdilution | 6 |
| Mattia Palmieri (209) | 2020 | 2015 to 2017 | Greece | Europe |  |  | 122 | 40 | EUCAST | HAI | Broth microdilution | 9 |
| McCracken (210) | 2011 | 2007 to 2009 | Canada | North America |  |  | 66 | 4 | CLSI | HAI | Broth microdilution | 8 |
| Medell (211) | 2013 | 2011 to 2011 | Cuba | South America |  | Respiratory | 53 | 1 | CLSI | HAI | Broth microdilution | 9 |
| Mehdi Meskini Heydarlou (212) | 2022 | 2019 to 2020 | Iran | Asia |  | Non respiratory | 92 | 10 | EUCAST | HAI | Broth microdilution | 9 |
| Mehmet Şah İpek (213) | 2016 | 2011 to 2014 | Turkey | Asia | NICU | Non respiratory | 39 | 1 | CLSI | HAI | Broth microdilution | 8 |
| MENGELOĞLU (214) | 2014 | 2010 to 2012 | Turkey | Asia |  |  | 77 | 3 | CLSI | HAI | Broth microdilution | 9 |
| Meqdad Saleh Ahmed(215) | 2023 | 2021 to 2021 | Iraq | Asia | ICU |  | 26 | 10 | CLSI | HAI | Broth microdilution | 8 |
| Mera A. Ababneh (216) | 2022 | 2017 to 2019 | Jordan | Asia |  | Non respiratory | 68 | 3 | CLSI | HAI | Broth microdilution | 9 |
| Mezzatesta (217) | 2014 | 2013 to 2013 | Italy | Europe | ICU |  | 52 | 0 | EUCAST | HAI | Broth microdilution | 7 |
| Michael D. Huband (218) | 2020 |  |  |  |  |  | 531 | 50 | CLSI | HAI | Broth microdilution | 8 |
| Michelle Lowe (219) | 2022 | 2019 to 2020 | South Africa | Africa |  |  | 127 | 6 | CLSI | HAI | Broth microdilution | 9 |
| Minakshi Gupta (220) | 2019 | 2015 to 2017 | India | Asia | Burn |  | 27 | 2 | CLSI | HAI | Broth microdilution | 9 |
| Al-Tamimi (221) | 2022 | 2010 to 2020 | Jordan | Asia |  |  | 622 | 14 | CLSI | HAI | Broth microdilution | 9 |
| Mohammad Hasan Namaei (222) | 2021 | 2018 to 2019 | Iran | Asia |  |  | 50 | 0 | CLSI | HAI | Broth microdilution | 9 |
| Mohammad Reza Kandehkar Ghahraman (223) | 2020 | 2017 to 2018 | Iran | Asia | ICU |  | 187 | 4 | CLSI | HAI | Broth microdilution | 9 |
| Mohd Saleem (224) | 2022 | 2019 to 2020 | Saudi Arabia | Asia | ICU |  | 82 | 4 | CLSI | HAI | Broth microdilution | 9 |
| Mohsin Khurshid (225) | 2020 | 2017 to 2018 | Pakistan | Asia |  | Non respiratory | 204 | 1 | CLSI | HAI | Broth microdilution | 7 |
| Mohsin Khurshid (226) | 2020 | 2017 to 2017 | Pakistan | Asia |  |  | 143 | 0 | CLSI | HAI | Broth microdilution | 7 |
| Mona Mohamed Al-Shamiri (227) | 2021 | 2018 to 2019 | China | Asia | ICU | Respiratory | 70 | 0 | CLSI | HAI | Broth microdilution | 9 |
| Moon (228) | 2013 | 2005 to 2011 | Korea | Asia |  | Non respiratory | 40 | 0 | CLSI | HAI | Broth microdilution | 8 |
| Morad Ali Ranaei (229) | 2020 | 2018 to 2018 | Iran | Asia |  |  | 141 | 0 | CLSI | HAI | Broth microdilution | 9 |
| Morubagal Raghavendra Rao (230) | 2022 |  | India | Asia | ICU |  | 149 | 1 | CLSI | HAI | Broth microdilution | 8 |
| Shah Mubashir HassaN (231) | 2022 | 2017 to 2018 | India | Asia |  |  | 14 | 0 | CLSI | HAI | Broth microdilution | 8 |
| MuthUSAmy (232) | 2016 | 2014 to 2014 | India | Asia |  |  | 100 | 10 | CLSI | HAI | Broth microdilution | 7 |
| Myung-Jin Choi (233) | 2014 |  | Korea | Asia |  |  | 5 | 0 | CLSI | HAI | Broth microdilution | 4 |
| Nabil Karah (234) | 2020 | 2013 to 2015 | Pakistan | Asia |  |  | 52 | 0 | CLSI | HAI | Broth microdilution | 7 |
| Nabila Benamrouche (235) | 2020 | 2012 to 2016 | Algeria | Africa |  |  | 92 | 5 | CLSI | HAI | Broth microdilution | 8 |
| Nancy Mohamed (236) | 2023 | 2017 to 2021 | Egypt | Asia |  |  | 67 | 0 | CLSI | CAI | Broth microdilution | 9 |
| Naoki Kohira (237) | 2023 | 2020 to 2020 | China | Asia |  |  | 279 | 8 | EUCAST | HAI | Broth microdilution | 9 |
| Nazir (238) | 2018 | 2017 to 2018 | India | Asia | NICU | Non respiratory | 48 | 0 | CLSI | HAI | Broth microdilution | 8 |
| Nazmul Hasan Muzahid (239) | 2023 | 2018 to 2020 | Malaysia | Asia |  |  | 27 | 1 | CLSI | HAI | Broth microdilution | 8 |
| Neda Yousefi Nojookambari (240) | 2021 | 2019 to 2020 | Iran | Asia | NICU |  | 60 | 0 | CLSI | HAI | Broth microdilution | 9 |
| Nemec (241) | 2009 |  | Netherlands | Europe |  |  | 20 | 0 | CLSI | HAI | Broth microdilution | 5 |
| Neveen M. Saleh (242) | 2020 |  | Egypt | Africa |  |  | 110 | 6 | CLSI | HAI | Broth microdilution | 7 |
| Nevine S Fam (243) | 2020 | 2015 to 2016 | Egypt | Africa | ICU |  | 17 | 9 | CLSI | HAI | Broth microdilution | 6 |
| Nevine S. Fam (244) | 2020 | 2015 to 2016 | Egypt | Africa |  |  | 22 | 0 | CLSI | HAI | Broth microdilution | 9 |
| Nguyen Van An (245) | 2023 | 2014 to 2021 | Vietnam | Asia |  | Non respiratory | 48 | 8 | CLSI | HAI | Broth microdilution | 6 |
| Noel-David Nogbou (246) | 2021 | 2017 to 2018 | South Africa | Africa |  |  | 100 | 0 | CLSI | HAI | Broth microdilution | 9 |
| Nordqvist (247) | 2016 | 2013 to 2013 | Sweden | Europe |  |  | 4 | 0 | EUCAST | HAI | Broth microdilution | 6 |
| OleksiUK (248) | 2014 |  | USA | North America |  |  | 18 | 6 | CLSI | HAI | Broth microdilution | 6 |
| Olga Perovic (249) | 2022 | 2017 to 2019 | South Africa | Africa |  | Non respiratory | 1944 | 24 | CLSI | HAI | Broth microdilution | 9 |
| Özbek (250) | 2010 | 2007 to 2007 | Turkey | Asia |  |  | 50 | 0 | CLSI | HAI | Broth microdilution | 7 |
| Ozger (251) | 2019 |  | Turkey | Asia |  | Respiratory | 10 | 3 | CLSI | HAI | Broth microdilution | 4 |
| Ozlem Aydemir (252) | 2022 | 2019 to 2021 | Turkey | Asia | ICU |  | 6 | 0 | EUCAST | HAI | Broth microdilution | 8 |
| PALUCHOWSKA (253) | 2017 | 2008 to 2013 | Poland | Europe |  |  | 155 | 1 | CLSI | CAI | Broth microdilution | 8 |
| Paola Di Carlo (254) | 2021 | 2018 to 2020 | Italy | Europe |  | Non respiratory | 20 | 1 | EUCAST | HAI | Broth microdilution | 9 |
| Park (255) | 2010 | 2006 to 2007 | Korea | Asia |  |  | 59 | 9 | CLSI | HAI | Broth microdilution | 8 |
| Park (256) | 2009 | 2007 to 2007 | Korea | Asia |  |  | 63 | 19 | CLSI | HAI | Broth microdilution | 7 |
| Parul Chaturvedi (257) | 2020 | 2018 to 2019 | India | Asia | ICU | Non respiratory | 30 | 0 | CLSI | HAI | Broth microdilution | 8 |
| Jia Peiyao (258) | 2022 | 2018 to 2018 | China | Asia |  |  | 114 | 0 | CLSI | HAI | Broth microdilution | 9 |
| Petros Ioannou (259) | 2023 | 2016 to 2021 | Greece | Europe |  |  | 16 | 3 | CLSI | HAI | Broth microdilution | 8 |
| Phee (260) | 2019 |  | UK | Europe |  |  | 5 | 1 | CLSI | HAI | Broth microdilution | 4 |
| Philipp Thelen (261) | 2022 | 2014 to 2018 | Germany | Europe |  | Respiratory | 211 | 5 | EUCAST | CAI | Broth microdilution | 9 |
| Piyatip Khuntayaporn (262) | 2021 | 2016 to 2017 | Thailand | Asia |  |  | 135 | 20 | CLSI | HAI | Broth microdilution | 9 |
| Potron (263) | 2019 | 2014 to 2015 | France | Europe | ICU |  | 4 | 2 | CLSI | HAI | Broth microdilution | 4 |
| Pournaras (264) | 2017 | 2015 to 2015 | Greece | Europe |  |  | 194 | 53 | EUCAST | HAI | Broth microdilution | 8 |
| Po-Yu Liu (265) | 2020 | 2019 to 2019 | Taiwan | Asia |  | Non respiratory | 199 | 17 | CLSI | HAI | Broth microdilution | 6 |
| Principe (266) | 2013 | 2004 to 2008 | Italy | Europe | ICU |  | 21 | 1 | CLSI | HAI | Broth microdilution | 6 |
| Purva Mathur (267) | 2022 | 2017 to 2018 | India | Asia | ICU |  | 346 | 17 | CLSI | HAI | Broth microdilution | 7 |
| Qi Wang (268) | 2020 | 2010 to 2018 | China | Asia |  |  | 926 | 1 | CLSI | HAI | Broth microdilution | 8 |
| Qingsong You (269) | 2023 | 2013 to 2017 | China | Asia |  |  | 89 | 12 | CLSI | HAI | Broth microdilution | 7 |
| Qiwen Yang (270) | 2020 | 2016 to 2018 | China | Asia |  |  | 982 | 91 | CLSI | HAI | Broth microdilution | 6 |
| Rama Chandra Reddy (271) | 2022 | 2018 to 2018 | India | Asia | ICU |  | 31 | 2 | EUCAST | HAI | Broth microdilution | 8 |
| Rasheed O. Aldossari (272) | 2021 | 2017 to 2020 | Saudi Arabia | Asia |  | Non respiratory | 51 | 2 | CLSI | CAI | Broth microdilution | 9 |
| Reale (273) | 2017 | 2011 to 2014 | Italy | Europe |  |  | 52 | 12 | EUCAST | HAI | Broth microdilution | 8 |
| Rehab El‑Sokkary (274) | 2021 | 2019 to 2019 |  |  | ICU | Non respiratory | 44 | 2 | CLSI | HAI | Broth microdilution | 8 |
| Han, Renru (275) | 2022 | 2018 to 2019 | China | Asia |  |  | 536 | 3 | CLSI | HAI | Broth microdilution | 9 |
| Rezai (276) | 2017 | 2014 to 2014 | Iran | Asia | ICU |  | 29 | 10 | CLSI | HAI | Broth microdilution | 8 |
| Rezzak Hamzeh (277) | 2012 | 2008 to 2011 | Syria | Asia |  |  | 260 | 18 | CLSI | HAI | Broth microdilution | 8 |
| Rolain (278) | 2016 | 2011 to 2012 | Qatar | Asia | ICU,CCU | Respiratory | 48 | 0 | CLSI | HAI | Broth microdilution | 7 |
| Romney M. Humphries (279) | 2023 | 2018 to 2020 |  | Europe |  |  | 537 | 65 | EUCAST | HAI | Broth microdilution | 9 |
| Rosales-Reyes (280) | 2017 | 2014 to 2014 | Mexico | North America |  |  | 112 | 0 | CLSI | HAI | Broth microdilution | 7 |
| Rozita Khodashahi (281) | 2022 | 2019 to 2020 | Iran | Asia | ICU | Non respiratory | 3 | 0 | CLSI | HAI | Broth microdilution | 8 |
| Russo (282) | 2019 | 2017 to 2018 | Italy | Europe |  | Non respiratory | 281 | 4 | EUCAST | HAI | Broth microdilution | 9 |
| S.M. Mortazavi (283) | 2020 | 2018 to 2019 | Iran | Asia |  |  | 80 | 71 | CLSI | HAI | Broth microdilution | 9 |
| Sachin S. Bhagwat (284) | 2021 | 2014 to 2018 | Greece | Europe |  |  | 181 | 103 | EUCAST | HAI | Broth microdilution | 9 |
| Sader (285) | 2018 | 2015 to 2017 | USA | North America | ICU | Respiratory | 170 | 15 | CLSI | HAI | Broth microdilution | 7 |
| Saeed Khoshnood (286) | 2020 | 2018 to 2019 | Iran | Asia |  |  | 70 | 2 | CLSI | HAI | Broth microdilution | 9 |
| Saeedeh Ebrahimi (287) | 2021 | 2017 to 2018 | Iran | Asia |  |  | 15 | 4 | CLSI | HAI | Broth microdilution | 8 |
| SAIDA MELIANI (288) | 2020 | 2015 to 2017 | Algeria | Africa |  |  | 60 | 0 | CLSI | HAI | Broth microdilution | 8 |
| [Samandeep Kaur](https://pubmed.ncbi.nlm.nih.gov/?term=Firoozeh+F&cauthor_id=36640263) (289) | 2023 | 2020 to 2020 | India | Asia |  | Non respiratory | 85 | 1 | CLSI | HAI | Broth microdilution | 7 |
| Samaneh Babaei (290) | 2021 | 2012 to 2018 | Iran | Asia | ICU |  | 200 | 0 | CLSI | HAI | Broth microdilution | 9 |
| Samaneh Pourajam (291) | 2022 | 2020 to 2020 | Iran | Asia | ICU |  | 35 | 0 | EUCAST | HAI | Broth microdilution | 8 |
| [Samira M Hamed](https://pubmed.ncbi.nlm.nih.gov/?term=Hamed+SM&cauthor_id=35935207) (292) | 2022 | 2020 to 2020 | Egypt | Asia |  |  | 20 | 1 | CLSI | HAI | Broth microdilution | 8 |
| Samonis (293) | 2012 | 2004 to 2011 | Greece | Europe |  | Respiratory | 1242 | 16 | CLSI | HAI | Broth microdilution | 7 |
| Sanja Jakovac (294) | 2021 | 2018 to 2018 | Bosnia | Europe |  |  | 61 | 1 | CLSI | HAI | Broth microdilution | 9 |
| Santosh Kumar Yadav (295) | 2020 | 2017 to 2017 | Nepal | Asia |  |  | 177 | 0 | CLSI | CAI | Broth microdilution | 9 |
| Sarah M. McLeod (296) | 2020 | 2016 to 2017 |  |  |  |  | 1,420 | 67 | CLSI | HAI | Broth microdilution | 6 |
| Seifert (297) | 2006 | 1990 to 2003 | Germany | Europe |  |  | 218 | 6 | CLSI | HAI | Broth microdilution | 7 |
| Selim Gorgun (298) | 2021 | 2018 to 2018 | Turkey | Asia |  |  | 68 | 12 | EUCAST | HAI | Broth microdilution | 9 |
| Sevillano (299) | 2012 | 2008 to 2009 | Bolivia | South America |  |  | 43 | 0 | CLSI | HAI | Broth microdilution | 7 |
| Seyed Naser Abdi, Reza Ghotaslou (300) | 2020 |  | Iran | Asia |  |  | 3 | 3 | CLSI | HAI | Broth microdilution | 6 |
| Shah (301) | 2015 | 2013 to 2013 | India | Asia |  | Non respiratory | 15 | 0 | CLSI | HAI | Broth microdilution | 7 |
| Shaimaa Mohamed Seleim (302) | 2022 | 2020 to 2021 | Egypt | Asia | ICU |  | 100 | 49 | EUCAST | HAI | Broth microdilution | 8 |
| Shamsi Khalid (303) | 2020 | 2017 to 2017 | India | Asia | NICU | Non respiratory | 3 | 0 | CLSI | HAI | Broth microdilution | 7 |
| Shazad Mushtaq (304) | 2020 | 2008 to 2018 | UK | Europe |  |  | 99 | 11 | EUCAST | HAI | Broth microdilution | 8 |
| Sheck (305) | 2017 | 2013 to 2014 | Russia | Europe |  |  | 527 | 10 | EUCAST | HAI | Broth microdilution | 7 |
| Shio-Shin Jean (306) | 2014 | 2007 to 2007 | Taiwan | Asia | ICU |  | 100 | 4 | CLSI | HAI | Broth microdilution | 8 |
| Shio-Shin Jean (307) | 2022 | 2016 to 2018 | Taiwan | Asia |  |  | 53 | 1 | CLSI | HAI | Broth microdilution | 9 |
| [Shubham Chauhan](https://www.apjtm.org/searchresult.asp?search=&author=Shubham+Chauhan&journal=Y&but_search=Search&entries=10&pg=1&s=0) (308) | 2022 | 2021 to 2022 | India | Asia |  |  | 87 | 13 | CLSI | HAI | Broth microdilution | 8 |
| Shun-Chung Hsueh (309) | 2018 | 2016 to 2017 | Taiwan | Asia |  | Non respiratory | 100 | 10 | CLSI | HAI | Broth microdilution | 8 |
| Shymaa A.Ali (310) | 2022 | 2020 to 2022 | Iraq | Asia | ICU |  | 100 | 1 | CLSI | HAI | Broth microdilution | 9 |
| Singkham (311) | 2018 | 2010-2011 | Thailand | Asia |  |  | 23 | 0 | CLSI | HAI | Broth microdilution | 6 |
| Sirigade Ruekit (312) | 2022 | 2017 to 2018 | Thailand | Asia |  |  | 97 | 0 | EUCAST | HAI | Broth microdilution | 9 |
| So Hyun Jun (313) | 2023 | 2009 to 2019 | Korea | Asia |  |  | 167 | 0 | CLSI | HAI | Broth microdilution | 9 |
| Soo Tein Ngoi (314) | 2021 | 2019 to 2020 | Malaysia | Asia |  |  | 54 | 10 | CLSI | HAI | Broth microdilution | 9 |
| Srinivas (315) | 2018 | 2013 to 2015 | USA | North America |  | Respiratory | 24 | 0 | CLSI | HAI | Broth microdilution | 8 |
| Strateva (316) | 2018 | 2014 to 2016 | Bulgaria | Europe |  |  | 226 | 0 | EUCAST | HAI | Broth microdilution | 9 |
| Surbhi Khurana (317) | 2021 | 2020 to 2020 | India | Asia | ICU |  | 26 | 0 | CLSI | HAI | Broth microdilution | 9 |
| Suriya Chandran (318) | 2023 | 2020 to 2022 | India | Asia |  |  | 150 | 6 | CLSI | HAI | Broth microdilution | 9 |
| SUSAN KHANJANI (319) | 2020 | 2018 to 2018 | Iran | Asia | ICU |  | 59 | 0 | CLSI | HAI | Broth microdilution | 9 |
| SUSAnnah L. McKay (320) | 2022 | 2013 to 2017 | USA | North America |  |  | 92 | 19 | CLSI | HAI | Broth microdilution | 8 |
| Sushma Yadav Boorgula (321) | 2022 | 2021 to 2021 | India | Asia |  |  | 54 | 1 | CLSI | HAI | Broth microdilution | 9 |
| [Swati Sharma](https://pubmed.ncbi.nlm.nih.gov/?term=Sharma+S&cauthor_id=36683677) (322) | 2022 | 2016 to 2018 | India | Asia |  |  | 365 | 9 | CLSI | HAI | Broth microdilution | 9 |
| Swati Sharma (323) | 2021 | 2017 to 2018 | India | Asia |  |  | 365 | 9 | CLSI | HAI | Broth microdilution | 9 |
| T. Kostyanev (324) | 2021 | 2016 to 2018 |  | Europe |  |  | 226 | 21 | EUCAST | HAI | Broth microdilution | 9 |
| Tada (325) | 2014 | 2012 to 2012 | Japan | Asia |  |  | 49 | 4 | CLSI | HAI | Broth microdilution | 6 |
| Tada (326) | 2015 | 2011 to 2013 | Vietnam | Asia | ICU | Respiratory | 93 | 4 | CLSI | HAI | Broth microdilution | 7 |
| Tada (327) | 2013 | 2008 to 2011 | Vietnam | Asia | ICU |  | 101 | 6 | CLSI | HAI | Broth microdilution | 7 |
| Taghreed A. Hafiz (328) | 2023 | 2020 to 2022 | Saudi Arabia | Asia | ICU |  | 115 | 17 | CLSI | HAI | Broth microdilution | 9 |
| Tamayo (329) | 2013 | 2001 to 2012 | Spain | Europe |  |  | 66 | 16 | CLSI | HAI | Broth microdilution | 8 |
| Tamayo-Legorreta (330) | 2015 | 2009 to 2010 | Mexico | South America |  |  | 25 | 0 | CLSI | HAI | Broth microdilution | 6 |
| Taniya Paiboonvong (331) | 2020 | 2016 to 2016 | Thailand | Asia |  |  | 278 | 41 | CLSI | HAI | Broth microdilution | 9 |
| Tatsuya Tada (332) | 2020 | 2015 to 2018 | Myanmar | Asia |  |  | 38 | 0 | CLSI | HAI | Broth microdilution | 7 |
| Tekin (333) | 2013 | 2008 to 2011 | Turkey | Asia | NICU |  | 24 | 0 | CLSI | HAI | Broth microdilution | 8 |
| Tiago Barcelos Valiatti (334) | 2022 | 2019 to 2019 | Brazil | South America |  |  | 27 | 0 | Other | HAI | Broth microdilution | 6 |
| Tripodi (335) | 2007 |  | Italy | Europe |  |  | 9 | 0 | CLSI | HAI | Broth microdilution | 6 |
| Tsitsopoulos (336) | 2016 | 2003 to 2012 | Greece | Europe |  | Non respiratory | 50 | 0 | CLSI | HAI: 51 CAI: 3 | Broth microdilution | 9 |
| Tuğba K. Atik (337) | 2018 | 2015 to 2015 | Turkey | Asia | ICU |  | 5 | 0 | CLSI | HAI | Broth microdilution | 6 |
| Umaira Ahsan (338) | 2022 | 2019 to 2020 | Pakistan | Asia | ICU |  | 150 | 11 | CLSI | HAI | Broth microdilution | 9 |
| van Belkum (339) | 2014 | 2006 to 2013 | Switzerland | Europe |  | Non respiratory | 27 | 0 | EUCAST | HAI | Broth microdilution | 4 |
| Ivana Goic-Barisic (340) | 2021 | 2009 to 2018 |  | Europe |  |  | 12 | 0 | EUCAST | HAI | Broth microdilution | 8 |
| Villalo (341) | 2011 | 1997 to 2007 | Spain | Europe |  |  | 814 | 0 | CLSI | HAI | Broth microdilution | 8 |
| Vincent Trebosc (342) | 2020 | 2017 to 2019 | Switzerland | Europe |  |  | 293 | 45 | CLSI | HAI | Broth microdilution | 7 |
| Waleed El-Kazzaz (343) | 2020 | 2017 to 2018 | Egypt | Africa |  |  | 23 | 6 | CLSI | HAI | Broth microdilution | 8 |
| Wang (344) | 2018 | 2009 to 2013 | China | Asia |  | Non respiratory | 269 | 0 | CLSI | HAI | Broth microdilution | 7 |
| Waseem Shah (345) | 2019 | 2015 to 2016 | Saudi Arabia | Asia |  |  | 135 | 0 | CLSI | HAI | Broth microdilution | 9 |
| Weerayuth Saelim (346) | 2021 | 2014 to 2015 | Thailand | Asia |  |  | 50 | 5 | CLSI | HAI | Broth microdilution | 9 |
| Wen Liang (347) | 2019 | 2015 to 2016 | China | Asia |  | Non respiratory | 15 | 0 | CLSI | HAI | Broth microdilution | 8 |
| Wing Yau (348) | 2009 | 1998 to 2006 | Australia | Oceania |  |  | 30 | 1 | CLSI | HAI | Broth microdilution | 6 |
| Wisplinghoff (349) | 2011 | 1995 to 2003 | USA | North America |  |  | 187 | 1 | CLSI | HAI | Broth microdilution | 9 |
| Xiaofen Liu (350) | 2016 |  | China | Asia |  |  | 12 | 0 | CLSI | HAI | Broth microdilution | 6 |
| Xiaomeng Dong (351) | 2015 | 2013 to 2014 | China | Asia |  |  | 24 | 9 | CLSI | HAI | Broth microdilution | 6 |
| Xiaomeng Dong (352) | 2014 | 2012 to 2013 | China | Asia |  |  | 25 | 3 | CLSI | HAI | Broth microdilution | 6 |
| Xing Wang (353) | 2021 | 2017 to 2018 | China | Asia | ICU |  | 88 | 0 | CLSI | HAI | Broth microdilution | 9 |
| Xingchen Bian (354) | 2021 | 2016 to 2017 | China | Asia |  |  | 64 | 0 | CLSI | HAI | Broth microdilution | 9 |
| Xingchen Bien (355) | 2019 | 2014 to 2014 | China | Asia |  |  | 9 | 0 | CLSI | HAI | Broth microdilution | 6 |
| Y.J. Kim (356) | 2011 | 2001 to 2010 | Korea | Asia |  |  | 34 | 0 | CLSI | HAI | Broth microdilution | 8 |
| Yan Bai (357) | 2014 | 2010 to 2010 | China | Asia |  |  | 73 | 0 | CLSI | HAI | Broth microdilution | 7 |
| Bahçe, Yasemin Genç (358) | 2022 | 2019 to 2021 | Turkey | Asia | ICU |  | 81 | 0 | CLSI | HAI | Broth microdilution | 9 |
| Yaw Adjei Anane (359) | 2020 | 2016 to 2017 | South Africa | Africa |  |  | 100 | 0 | CLSI | HAI | Broth microdilution | 5 |
| Yawei Zhang (360) | 2020 | 2016 to 2016 | China | Asia |  |  | 33 | 0 | CLSI | HAI | Broth microdilution | 7 |
| Yazdansetad (361) | 2019 | 2013 to 2013 | Iran | Asia |  | Non respiratory | 65 | 8 | CLSI | HAI | Broth microdilution | 8 |
| Yiahi Gu (362) | 2022 | 2009 to 2018 | China | Asia |  | Non respiratory | 47 | 1 | CLSI | HAI | Broth microdilution | 8 |
| Yi-Fan Hu (363) | 2017 | 2012 to 2013 | Taiwan | Asia |  | Non respiratory | 108 | 0 | CLSI | HAI | Broth microdilution | 8 |
| Yi-Tzu Lee (364) | 2020 | 2010 to 2018 | Taiwan | Asia | ICU |  | 88 | 9 | CLSI | HAI | Broth microdilution | 8 |
| Yong-Hak Kim (365) | 2015 | 1999 to 2009 | Korea | Asia |  |  | 50 | 0 | CLSI | CAI | Broth microdilution | 8 |
| Yu Lin Lee (366) | 2019 | 2018 to 2018 | Taiwan | Asia |  | Non respiratory | 188 | 16 | CLSI | HAI | Broth microdilution | 9 |
| Yucel Duman (367) | 2022 | 2021 to 2021 | Turkey | Asia | ICU | Non respiratory | 21 | 0 | EUCAST | HAI | Broth microdilution | 8 |
| Zahra Meshkat (368) | 2021 | 2014 to 2015 | Iran | Asia |  |  | 270 | 0 | CLSI | HAI: 91 CAI: 44 | Broth microdilution | 9 |
| Zapor (369) | 2010 | 2007 to 2008 | USA | North America |  |  | 200 | 0 | CLSI | HAI | Broth microdilution | 8 |
| Zarrilli (370) | 2012 | 2010 to 2011 | Italy | Europe | NICU |  | 34 | 0 | CLSI | HAI | Broth microdilution | 8 |
| Zhang (371) | 2019 | 2015 to 2016 | China | Asia |  | Non respiratory | 88 | 1 | CLSI | HAI | Broth microdilution | 8 |
| Zhiyong Wei (372) | 2022 | 2017 to 2022 | China | Asia |  | Non respiratory | 110 | 1 | CLSI | HAI | Broth microdilution | 9 |
| Zhu (373) | 2018 | 2014 to 2014 | China | Asia |  |  | 21 | 13 | CLSI | HAI | Broth microdilution | 6 |
| Wang Liang (374) | 2011 | 2009 to 2010 | China | Asia |  |  | 14 | 0 | CLSI | HAI | Broth microdilution | 7 |
| Hu (375) | 2011 | 2008 to 2009 | China | Asia |  |  | 71 | 0 | CLSI | HAI | Broth microdilution | 7 |
| Franolić-KUKina (376) | 2011 | 2008 to 2008 | Croatia | Europe |  |  | 34 | 0 | CLSI | HAI | Broth microdilution | 6 |
| Mammina (377) | 2011 | 2010 to 2010 | Italy | Europe |  |  | 45 | 0 | CLSI | HAI | Broth microdilution | 6 |
| Cai (378) | 2010 | 2006 to 2007 | China | Asia |  |  | 70 | 0 | CLSI | HAI | Broth microdilution | 7 |
| Giannouli (379) | 2010 | 2006 to 2007 | Italy | Europe |  |  | 71 | 0 | CLSI | HAI | Broth microdilution | 8 |
| Routsi (380) | 2010 | 2004 to 2006 | Greece | Europe |  |  | 96 | 0 | CLSI | HAI | Broth microdilution | 9 |
| Shio-Shin Jean (381) | 2009 | 2005 to 2005 | Taiwan | Asia |  |  | 167 | 10 | CLSI | HAI | Broth microdilution | 8 |
| McCracken (382) | 2009 | 2007 to 2009 | Canada | North America |  |  | 26 | 3 | CLSI | HAI | Broth microdilution | 7 |
| Principe (383) | 2009 | 2004 to 2005 | Italy | Europe |  |  | 22 | 1 | CLSI | HAI | Broth microdilution | 6 |
| Zarrilli (384) | 2008 | 2004 to 2005 | Lebanon | Asia |  |  | 17 | 0 | CLSI | HAI | Broth microdilution | 6 |
| Mezzatesta (385) | 2008 | 2002 to 2003 | Italy | Europe |  |  | 107 | 1 | CLSI | HAI | Broth microdilution | 8 |
| Kwan Soo Ko (386) | 2007 | 2002 to 2006 | Korea | Asia |  |  | 214 | 81 | CLSI | HAI | Broth microdilution | 7 |
| Hawley (387) | 2006 | 2003 to 2005 | USA | North America |  |  | 142 | 3 | CLSI | HAI | Broth microdilution | 8 |
| Dagher (388) | 2019 | 2016 to 2016 | Lebanon | Asia |  | Respiratory | 31 | 0 | EUCAST | HAI | Broth microdilution | 7 |
| Qamar (389) | 2019 | 2015 to 2016 | Pakistan | Asia | Pediatric |  | 29 | 0 | CLSI | HAI, CAI | Broth microdilution | 6 |
| Brasiliense (390) | 2019 | 2012 to 2016 | Brazil | South America |  |  | 3 | 0 | EUCAST | HAI, CAI | Broth microdilution | 6 |
| Ramadan (391) | 2018 | 2017 to 2017 | Egypt | Africa | SICU |  | 50 | 0 | CLSI | HAI | Broth microdilution | 7 |
| Kaskatepe (392) | 2018 |  | Turkey | Europe |  |  | 7 | 0 | EUCAST | HAI | Broth microdilution | 6 |
| Yousef Khan (393) | 2017 | 2009 to 2013 | Qatar | Asia |  | Non respiratory | 8 | 0 | CLSI | HAI | Broth microdilution | 8 |
| SHAH (394) | 2014 | 2013 to 2013 | India | Asia |  |  | 18 | 0 | CLSI | HAI | Broth microdilution | 8 |
| Principe (395) | 2014 | 2011 to 2011 | Italy | Europe |  |  | 571 | 0 | CLSI | HAI | Broth microdilution | 6 |
| Sieniawski (396) | 2013 | 2011 to 2011 | Poland | Europe |  |  | 140 | 14 | CLSI | HAI | Broth microdilution | 7 |
| Bahador (397) | 2012 | 2011 to 2012 | Iran | Asia | ICU |  | 79 | 16 | CLSI | HAI | Broth microdilution | 8 |
| Alsultan (398) | 2014 | 2012 to 2012 | Saudi Arabia | Asia |  |  | 64 | 2 | CLSI | HAI | Broth microdilution | 7 |

| article code | Author name | Number of *A.baumannii* | Number of colistin resistant strains | AST Method |
| --- | --- | --- | --- | --- |
| 141 | Ajay Kumar P (399) | 6 | 0 | Agar dilution |
| 355 | Al-Agamy (400) | 40 | 2 | Agar dilution |
| 443 | Alsultan (401) | 271 | 2 | Agar dilution |
| 329 | Al-Sultan (402) | 83 | 0 | Agar dilution |
| 312 | Chunmei Ying (403) | 27 | 0 | Agar dilution |
| 90 | David O. Ogbolu (404) | 21 | 7 | Agar dilution |
| 506 | Dizbay (405) | 25 | 0 | E-test |
| 945 | Fariba Naeimi Mazraeh (406) | 70 | 9 | Agar dilution |
| 906 | Ghayda Al-Hashem (407) | 270 | 8 | Agar dilution |
| 414 | Gurung (408) | 69 | 1 | Agar dilution |
| 808 | Rhim, Hajer(409) | 618 | 0 | Agar dilution |
| 549 | Hui Wang (410) | 221 | 0 | Agar dilution |
| 239 | HyUKmin Lee (411) | 34 | 0 | Agar dilution |
| 96 | Jeon (412) | 147 | 2 | Agar dilution |
| 109 | Jiaying Li (413) | 35 | 5 | Agar dilution |
| 547 | Jung Hoon Lee (414) | 142 | 0 | Agar dilution |
| 761 | Khalid I. AlQumaizi (415) | 30 | 0 | Agar dilution |
| 393 | Lee (416) | 121 | 0 | Agar dilution |
| 162 | Lei Gao (417) | 2031 | 146 | Agar dilution |
| 413 | Mei-Hui Lee (418) | 291 | 0 | E-test |
| 336 | Nageeb (419) | 10 | 2 | Agar dilution |
| 342 | najar peerayeh (420) | 157 | 0 | Disk diffusion |
| 7 | Namiganda (421) | 19 | 0 | Agar dilution |
| 800 | Naveed Ahmed(422) | 55 | 0 | Agar dilution |
| 313 | Rodriguez (423) | 5 | 2 | Agar dilution |
| 919 | Tao Chen (424) | 54 | 0 | Agar dilution |
| 171 | Vasconcellos (425) | 71 | 1 | Agar dilution |
| 119 | Wen-juan Wei (426) | 50 | 3 | Agar dilution |
| 335 | Wen-Shyang Hsieh (427) | 577 | 0 | Agar dilution |
| 398 | Wentao Ni (428) | 70 | 0 | Agar dilution |
| 263 | Wen-Wei Ku (429) | 667 | 0 | Agar dilution |
| 394 | Ya-Sung Yang (430) | 71 | 1 | Agar dilution |
| 716 | Yayun Zhao (431) | 443 | 30 | Agar dilution |
| 260 | Ying Li (432) | 106 | 1 | Agar dilution |
| 116 | Yi-Tzu Lee (433) | 114 | 1 | Agar dilution |
| 332 | Yu-Chung Chuang (434) | 134 | 0 | Agar dilution |
| 259 | Yu-Shan Huang (435) | 146 | 0 | Agar dilution |
| 775 | Zahra Dargahi(436) | 121 | 21 | Agar dilution |
| 127 | Moehario, L. H (437) | 17 | 1 | Disk diffusion |
| 150 | ABHISHEKMEHTA (438) | 23 | 1 | Disk diffusion |
| 60 | Adel El mekes (439) | 94 | 0 | Disk diffusion |
| 132 | Ahdi (440) | 100 | 23 | Disk diffusion |
| 144 | Ahmed B. Mahmoud (441) | 50 | 16 | Disk diffusion |
| 172 | Akrami (442) | 35 | 0 | Disk diffusion |
| 257 | Al Atrouni (443) | 119 | 0 | Disk diffusion |
| 153 | Ala’a Matalka (444) | 4 | 0 | Disk diffusion |
| 110 | Alfadli (445) | 17 | 1 | Disk diffusion |
| 324 | Alharbi (446) | 18 | 3 | Disk diffusion |
| 361 | Aliakbarzade (447) | 103 | 20 | Disk diffusion |
| 315 | AlmOmani (448) | 121 | 0 | Disk diffusion |
| 178 | Alp (449) | 52 | 0 | Disk diffusion |
| 735 | Ambreen Fatima(450) | 365 | 0 | Disk diffusion |
| 67 | Amir Emami (451) | 38 | 2 | Disk diffusion |
| 902 | André Adaimé (452) | 11 | 1 | Disk diffusion |
| 499 | Andriamanantena (453) | 53 | 0 | Disk diffusion |
| 146 | Ankita Chaurasia (454) | 3 | 0 | Disk diffusion |
| 167 | Ansari (455) | 30 | 3 | Disk diffusion |
| 63 | Armin (456) | 171 | 0 | Disk diffusion |
| 43 | Arzu NAZLI ZEKA (457) | 60 | 0 | Disk diffusion |
| 10 | Asim Ali Shah (458) | 78 | 0 | Disk diffusion |
| 103 | Asma Ejaz (459) | 20 | 0 | Disk diffusion |
| 46 | Aya A. Khodier (460) | 48 | 0 | Disk diffusion |
| 288 | Azizi (461) | 100 | 13 | Disk diffusion |
| 49 | Babaei (462) | 50 | 3 | Disk diffusion |
| 277 | Bagheri Josheghani (463) | 124 | 0 | Disk diffusion |
| 123 | Bakhta Bouharkat (464) | 17 | 17 | Disk diffusion |
| 50 | Balkhair (465) | 169 | 0 | Disk diffusion |
| 124 | Ballouz (466) | 90 | 0 | Disk diffusion |
| 115 | Behnam Hashemi (467) | 150 | 15 | Disk diffusion |
| 912 | Bence Balázs (468) | 681 | 7 | Disk diffusion |
| 541 | Brahmi (469) | 102 | 0 | Disk diffusion |
| 112 | Brajesh Raj Chaudhary (470) | 27 | 0 | Disk diffusion |
| 528 | Cetin (471) | 66 | 8 | Disk diffusion |
| 165 | CHANDRA BEHERA (472) | 82 | 9 | Disk diffusion |
| 949 | Chandrakant Prasad (473) | 188 | 97 | Disk diffusion |
| 416 | Chaudhary (474) | 371 | 116 | Disk diffusion |
| 360 | chittawatanarat (475) | 58 | 0 | Disk diffusion |
| 440 | Cicek (476) | 6 | 0 | Disk diffusion |
| 331 | Coskun (477) | 87 | 0 | Disk diffusion |
| 8 | Coskun (478) | 96 | 0 | Disk diffusion |
| 68 | Das (479) | 8 | 0 | Disk diffusion |
| 805 | David Darvishni(480) | 22 | 5 | Disk diffusion |
| 417 | De Francesco (481) | 167 | 0 | Disk diffusion |
| 325 | Dedeić-Ljubović (482) | 257 | 0 | Disk diffusion |
| 212 | DİREKEL (483) | 354 | 3 | Disk diffusion |
| 219 | Ergönül (484) | 256 | 15 | Disk diffusion |
| 27 | Eslami (485) | 300 | 0 | Disk diffusion |
| 798 | Essamedin M. Negm (486) | 4723 | 179 | Disk diffusion |
| 194 | Fallah (487) | 100 | 4 | Disk diffusion |
| 403 | Farajnia (488) | 100 | 19 | Disk diffusion |
| 302 | Farsiani (489) | 36 | 0 | Disk diffusion |
| 406 | Farzana (490) | 15 | 0 | Disk diffusion |
| 470 | Fernández Cuenca (491) | 221 | 0 | Disk diffusion |
| 178 | Fizza Khan (492) | 73 | 0 | Disk diffusion |
| 83 | Frikh (493) | 90 | 0 | Disk diffusion |
| 161 | Gerald Mboowa (494) | 75 | 14 | Disk diffusion |
| 293 | Gholami (495) | 60 | 0 | Disk diffusion |
| 215 | Gupta (496) | 10 | 0 | Disk diffusion |
| 153 | HASAN (497) | 22 | 0 | Disk diffusion |
| 1 | HASBI ARA MOSTOFA (498) | 24 | 3 | Disk diffusion |
| 154 | HEIDARY (499) | 100 | 0 | Disk diffusion |
| 140 | Hend Ben Lakhal (500) | 32 | 0 | Disk diffusion |
| 911 | Hosne Ara (501) | 39 | 5 | Disk diffusion |
| 176 | Huda Rasheed Tawfeeq (502) | 54 | 7 | Disk diffusion |
| 177 | Iman Abbas Ali (503) | 19 | 1 | Disk diffusion |
| 187 | Izadi (504) | 100 | 12 | Disk diffusion |
| 165 | Jalali Y (505) | 18 | 0 | Disk diffusion |
| 437 | Japoni-Nejad (506) | 56 | 4 | Disk diffusion |
| 211 | Jasemi (507) | 401 | 0 | Disk diffusion |
| 913 | Kamelia Banihashemi (508) | 50 | 2 | Disk diffusion |
| 420 | Kapoor (509) | 35 | 0 | Disk diffusion |
| 359 | Kapoor (510) | 92 | 4 | Disk diffusion |
| 298 | Kara (511) | 134 | 2 | Disk diffusion |
| 436 | Karmostaji (512) | 131 | 0 | Disk diffusion |
| 531 | Katsaragakis (513) | 52 | 0 | Disk diffusion |
| 111 | Kaur (514) | 116 | 3 | Disk diffusion |
| 166 | Khaled Salama (515) | 17 | 0 | Disk diffusion |
| 290 | Khatun (516) | 25 | 0 | Disk diffusion |
| 295 | Khorsi (517) | 125 | 0 | Disk diffusion |
| 130 | Khoshnood (518) | 100 | 0 | Disk diffusion |
| 272 | Kooti (519) | 200 | 0 | Disk diffusion |
| 18 | Kovacevic (520) | 146 | 1 | Disk diffusion |
| 117 | Krit Thirapanmethee (521) | 183 | 31 | Disk diffusion |
| 115 | Lachhab (522) | 13 | 0 | Disk diffusion |
| 77 | Lakshmi Kakhandki (523) | 47 | 0 | Disk diffusion |
| 55 | Leungtongkam (524) | 339 | 0 | Disk diffusion |
| 60 | Levy-Blitchein (525) | 80 | 4 | Disk diffusion |
| 944 | Moni Mahto (526) | 326 | 27 | Disk diffusion |
| 101 | Maamar (527) | 12 | 0 | Disk diffusion |
| 29 | Mahdi, A (528) | 40 | 6 | Disk diffusion |
| 294 | Marie (529) | 54 | 0 | Disk diffusion |
| 32 | Marion (530) | 39 | 0 | Disk diffusion |
| 289 | Mathlouthi (531) | 25 | 0 | Disk diffusion |
| 428 | Metan (532) | 11 | 0 | Disk diffusion |
| 369 | Metan (533) | 29 | 1 | Disk diffusion |
| 905 | Mina Aghamali (534) | 27 | 1 | Disk diffusion |
| 26 | Mir Tahir Hussain Talpur (535) | 16 | 6 | Disk diffusion |
| 724 | Mitra Kar (536) | 45 | 0 | Disk diffusion |
| 307 | MODARRESI (537) | 65 | 8 | Disk diffusion |
| 138 | Mohajeri (538) | 33 | 5 | Disk diffusion |
| 284 | Mohajeri (539) | 42 | 5 | Disk diffusion |
| 409 | Mohajeri (540) | 104 | 11 | Disk diffusion |
| 139 | Mohajeri (541) | 75 | 8 | Disk diffusion |
| 921 | Mohammad Masoud Emami Meybodi (542) | 282 | 11 | Disk diffusion |
| 99 | Mohd Rani (543) | 128 | 0 | Disk diffusion |
| 38 | Mohsin Khurshid (544) | 156 | 0 | Disk diffusion |
| 385 | Moisoiu (545) | 213 | 2 | Disk diffusion |
| 910 | Mojtaba Anvarinejad (546) | 95 | 0 | Disk diffusion |
| 28 | Mojtaba Moosavian (547) | 124 | 1 | Disk diffusion |
| 47 | Monfared (548) | 118 | 9 | Disk diffusion |
| 151 | Moosavian (549) | 151 | 2 | Disk diffusion |
| 35 | Mostafa A. Mohammed (550) | 100 | 2 | Disk diffusion |
| 810 | Mostafa Alavi-Moghaddam(551) | 121 | 0 | Disk diffusion |
| 363 | Mózes (552) | 160 | 0 | Disk diffusion |
| 149 | N Shashikala (553) | 24 | 6 | Disk diffusion |
| 104 | N. El Hamzaoui (554) | 20 | 4 | Disk diffusion |
| 21 | Nahla Shazli Abdel Azim (555) | 6 | 2 | Disk diffusion |
| 269 | Najar Peerayeh (556) | 123 | 0 | Agar dilution |
| 119 | NANCY M. ATTIA (557) | 21 | 1 | Disk diffusion |
| 799 | Narges Moradi(558) | 72 | 0 | Disk diffusion |
| 142 | Nguyen Tuan Anh (559) | 116 | 13 | Disk diffusion |
| 408 | Nikasa (560) | 65 | 1 | Disk diffusion |
| 432 | Niranjan (561) | 30 | 0 | Disk diffusion |
| 930 | Noha Alaa Eldin Fahim (562) | 128 | 0 | Disk diffusion |
| 25 | Noori (563) | 100 | 3 | Disk diffusion |
| 952 | Nureen Zahra (564) | 90 | 0 | Disk diffusion |
| 242 | Odewale (565) | 11 | 0 | Disk diffusion |
| 154 | Olusolabomi J Idowu (566) | 7 | 0 | Disk diffusion |
| 350 | Oncul (567) | 186 | 0 | Disk diffusion |
| 351 | Perween (568) | 205 | 3 | Disk diffusion |
| 933 | R. Ghimire (569) | 29 | 3 | Disk diffusion |
| 12 | Rabab R Makharita (570) | 39 | 8 | Disk diffusion |
| 346 | Rafei (571) | 4 | 0 | Disk diffusion |
| 68 | Rashin Amiri (572) | 72 | 44 | Disk diffusion |
| 564 | Ruiz (573) | 1532 | 0 | Disk diffusion |
| 310 | Rynga (574) | 100 | 3 | Disk diffusion |
| 175 | S Ozyurt (575) | 67 | 0 | Disk diffusion |
| 940 | S Jayashree (576) | 50 | 0 | Disk diffusion |
| 94 | Said (577) | 50 | 0 | Disk diffusion |
| 41 | Salehi (578) | 180 | 0 | E-test |
| 238 | Salimizand (579) | 30 | 0 | Disk diffusion |
| 300 | Salimizand (580) | 30 | 0 | Disk diffusion |
| 49 | Sandeep Kumar Tipparthi (581) | 20 | 0 | Disk diffusion |
| 776 | Sanjana Rajkumari (582) | 108 | 0 | Disk diffusion |
| 63 | Santosh Gunasekaran (583) | 31 | 0 | Disk diffusion |
| 11 | Santosh Kumar Yadav (584) | 177 | 0 | Disk diffusion |
| 114 | Sarikhani (585) | 108 | 45 | Disk diffusion |
| 961 | Shahriar Sepahvand (586) | 100 | 7 | Disk diffusion |
| 792 | Shahriar Sepahvand(587) | 100 | 34 | Disk diffusion |
| 401 | Shoja (588) | 206 | 0 | Disk diffusion |
| 268 | Shrestha (589) | 122 | 0 | Disk diffusion |
| 195 | Sileem (590) | 40 | 6 | Disk diffusion |
| 407 | Singla(591) | 82 | 0 | Disk diffusion |
| 458 | Sohrabi (592) | 100 | 19 | Disk diffusion |
| 554 | Souli (593) | 100 | 3 | Disk diffusion |
| 721 | Swati Mahich (594) | 33 | 0 | Disk diffusion |
| 138 | Surbhi Khurana (317) | 47 | 4 | Disk diffusion |
| 76 | Tahmasebi (595) | 30 | 5 | Disk diffusion |
| 205 | Tarashi (596) | 189 | 1 | Disk diffusion |
| 1 | Tayebi (597) | 150 | 0 | Disk diffusion |
| 730 | Tetyana Valeriyivna Denysko (598) | 42 | 0 | Disk diffusion |
| 66 | Tewari (599) | 45 | 0 | Disk diffusion |
| 41 | Thao T.B. Nguyen (600) | 50 | 0 | Disk diffusion |
| 65 | Thomas (601) | 399 | 0 | Disk diffusion |
| 58 | Tohamy (602) | 12 | 2 | Disk diffusion |
| 10 | TRAN-THI NGOC-VAN (603) | 4 | 0 | Disk diffusion |
| 383 | Trehan (604) | 7 | 0 | Disk diffusion |
| 159 | Tuğba ARSLAN GÜLEN (605) | 326 | 0 | Disk diffusion |
| 98 | Udomluk Leungtongkam (606) | 230 | 0 | Disk diffusion |
| 920 | V. Kondratiuk (607) | 53 | 4 | Disk diffusion |
| 960 | V.L. Nag (608) | 439 | 4 | Disk diffusion |
| 297 | Vaidya (609) | 84 | 8 | Disk diffusion |
| 501 | Viswanathan (610) | 32 | 2 | Disk diffusion |
| 118 | Wang (611) | 184 | 0 | Disk diffusion |
| 37 | Zaha (612) | 192 | 2 | Disk diffusion |
| 14 | Zahra Moulana (613) | 50 | 0 | Disk diffusion |
| 168 | Zain Alabadeen Karamya (614) | 216 | 2 | Disk diffusion |
| 173 | Zarifi (615) | 140 | 3 | Disk diffusion |
| 229 | Zilberberg (616) | 2086 | 112 | Disk diffusion |
| 155 | Al-Agamy (617) | 27 | 8 | E-test |
| 938 | Alireza Japoni-Nejad (618) | 80 | 4 | E-test |
| 279 | Al-Obeid (619) | 1044 | 0 | E-test |
| 459 | Al-Sweih (620) | 94 | 7 | E-test |
| 213 | ALTUN (621) | 44 | 0 | E-test |
| 137 | Andrea J. Grisold (622) | 114 | 6 | E-test |
| 278 | Armin (623) | 198 | 25 | E-test |
| 456 | Asadollahi (624) | 23 | 0 | E-test |
| 126 | Ashmita Paudel (625) | 71 | 2 | E-test |
| 434 | Baadani (626) | 1307 | 23 | E-test |
| 390 | Bahador (627) | 100 | 6 | E-test |
| 404 | Bakour (628) | 71 | 0 | Disk diffusion |
| 127 | Castilho (629) | 84 | 7 | Disk diffusion |
| 494 | Chen Liang-Yu (630) | 439 | 0 | Agar dilution |
| 327 | Cherkaoui (631) | 27 | 0 | E-test |
| 112 | Chmielarczyk (632) | 125 | 0 | E-test |
| 254 | Chmielarczyk (633) | 125 | 1 | E-test |
| 389 | CICEK (634) | 101 | 0 | E-test |
| 326 | Cikman (635) | 40 | 1 | E-test |
| 421 | Decousser (636) | 6 | 0 | E-test |
| 14 | Defaee (637) | 74 | 23 | E-test |
| 159 | Deylam (638) | 35 | 2 | E-test |
| 276 | Direkel (639) | 354 | 3 | E-test |
| 370 | Ece (640) | 98 | 0 | E-test |
| 308 | Elabd (641) | 108 | 5 | E-test |
| 438 | ERGIN (642) | 100 | 2 | E-test |
| 258 | Farshadzadeh (643) | 69 | 0 | E-test |
| 22 | Farzaneh Ahmadi Khatiri (644) | 91 | 0 | E-test |
| 430 | Fonseca (645) | 28 | 0 | E-test |
| 365 | Galani (646) | 14 | 4 | E-test |
| 535 | Galani (647) | 226 | 9 | E-test |
| 156 | Goudarzi (648) | 105 | 0 | E-test |
| 156 | Gunasekaran Santhi (649) | 16 | 2 | E-test |
| 341 | GÜVEN (650) | 139 | 4 | E-test |
| 559 | Haddad (651) | 10 | 0 | E-test |
| 282 | Handal (652) | 69 | 0 | E-test |
| 252 | Hasanin (653) | 30 | 0 | E-test |
| 560 | Héritier (654) | 8 | 0 | Disk diffusion |
| 931 | I. Gajic (655) | 13 | 0 | E-test |
| 454 | Izdebski (656) | 30 | 0 | E-test |
| 474 | Jankowski (657) | 67 | 1 | E-test |
| 98 | Jimenez-Guerra (658) | 90 | 8 | E-test |
| 424 | Jinsook Lim (659) | 45 | 0 | E-test |
| 725 | Jovana Kabic(660) | 752 | 30 | E-test |
| 221 | Karah (661) | 28 | 0 | E-test |
| 426 | Karaoglan (662) | 50 | 2 | E-test |
| 16 | Kheshti (663) | 20 | 0 | E-test |
| 489 | Kusradze (664) | 12 | 0 | Disk diffusion |
| 92 | Lee (665) | 37 | 0 | E-test |
| 61 | Leelasupasri (666) | 15 | 0 | E-test |
| 270 | Mahdian (667) | 37 | 0 | E-test |
| 3 | Malekzadegan (668) | 18 | 0 | E-test |
| 448 | Manageiro (669) | 116 | 1 | E-test |
| 451 | Maraki (670) | 137 | 8 | E-test |
| 815 | Marwa Shabban (671) | 14 | 2 | E-test |
| 73 | Mathlouthi (672) | 25 | 0 | E-test |
| 322 | Mavroidi (673) | 42 | 14 | E-test |
| 145 | Meijie Jiang (674) | 195 | 0 | E-test |
| 446 | Miyasaki (675) | 121 | 11 | E-test |
| 152 | Mohammadi (676) | 103 | 11 | E-test |
| 155 | Mohan B. Sannathimmappa (677) | 1890 | 19 | E-test |
| 402 | Mohanty (678) | 50 | 3 | E-test |
| 82 | Mojtaba Varshochi (679) | 50 | 1 | E-test |
| 4 | Monireh Kamali (680) | 113 | 16 | E-test |
| 108 | Mosavat (681) | 39 | 0 | E-test |
| 487 | Nakwan (682) | 8 | 0 | Disk diffusion |
| 158 | Nancy G Banoub (683) | 51 | 2 | E-test |
| 352 | Obeidat (684) | 64 | 0 | E-test |
| 287 | Oikonomou (685) | 1116 | 86 | E-test |
| 45 | Oliva (686) | 14 | 2 | E-test |
| 546 | Oteo (687) | 100 | 0 | E-test |
| 509 | Pankuch (688) | 25 | 0 | E-test |
| 150 | Papadimitriou-Olivgeris (689) | 129 | 1 | E-test |
| 339 | Pasanen (690) | 55 | 0 | E-test |
| 552 | Pierre Bogaerts (691) | 18 | 0 | E-test |
| 502 | Pongpech (692) | 30 | 0 | Agar dilution |
| 476 | Queenan (693) | 514 | 27 | E-test |
| 163 | Rashmi Karki (694) | 27 | 0 | E-test |
| 500 | Reddy (695) | 348 | 8 | E-test |
| 25 | Ritvan Kara Ali (696) | 28 | 2 | E-test |
| 717 | Saeed Khoshnood(697) | 50 | 0 | E-test |
| 103 | Salehi (698) | 125 | 0 | E-test |
| 161 | ŞANAL (699) | 30 | 2 | E-test |
| 146 | Savari (700) | 120 | 0 | E-test |
| 924 | Sayed Nassereddin Mostafavi (701) | 254 | 0 | E-test |
| 93 | Seifert (702) | 286 | 38 | E-test |
| 319 | Senok (703) | 12 | 1 | E-test |
| 126 | Sepahvand (704) | 100 | 11 | E-test |
| 207 | Sepahvand (705) | 100 | 6 | Disk diffusion |
| 822 | Shahriar Sepahvand(706) | 100 | 13 | E-test |
| 450 | Shao-Xing Dong (707) | 100 | 0 | E-test |
| 788 | Shimaa H. Mostafa (708) | 55 | 0 | E-test |
| 904 | Shirin Afhami (709) | 128 | 11 | E-test |
| 125 | Shoja (710) | 40 | 0 | E-test |
| 206 | Shoja (711) | 124 | 1 | E-test |
| 180 | Shokri (712) | 110 | 0 | E-test |
| 364 | SOUBIROU (713) | 5 | 0 | E-test |
| 562 | SPENCE (714) | 287 | 3 | E-test |
| 318 | Spiliopoulou (715) | 441 | 0 | E-test |
| 345 | Srivastava (716) | 12 | 10 | E-test |
| 88 | Sweta Singh (717) | 635 | 0 | E-test |
| 538 | Tan (718) | 13 | 0 | E-test |
| 706 | Tanya V. Strateva (719) | 73 | 0 | E-test |
| 185 | Tsioutis (720) | 5 | 1 | E-test |
| 334 | Tunyapanit (721) | 100 | 3 | E-test |
| 174 | UZUNOGLU (722) | 135 | 0 | E-test |
| 381 | Vakili (723) | 60 | 7 | E-test |
| 264 | Vien Le Minh (724) | 74 | 0 | E-test |
| 480 | Wang-Huei Sheng (725) | 32 | 1 | E-test |
| 378 | Wattal (726) | 450 | 2 | Disk diffusion |
| 204 | YAVAŞ (727) | 18 | 0 | E-test |
| 384 | Zeka (728) | 60 | 0 | E-test |
| 548 | Zhou (729) | 1018 | 173 | E-test |
| 97 | Ziolkowaski (730) | 187 | 0 | E-test |
| 742 | Zohreh Ghalavand (731) | 74 | 0 | E-test |
| 190 | Mavroidi (732) | 224 | 23 | E-test |
| 280 | Porwal (733) | 5 | 0 | E-test |
| 129 | Mahmoudi (734) | 86 | 0 | Disk diffusion |

1. Balkhair A, Al Saadi K, Al Adawi B. Epidemiology and mortality outcome of carbapenem-and colistin-resistant Klebsiella pneumoniae, Escherichia coli, Acinetobacter baumannii, and Pseudomonas aeruginosa bloodstream infections. IJID regions. 2023;7:1-5.

2. Maleki A, Kaviar VH, Koupaei M, Haddadi MH, Kalani BS, Valadbeigi H, et al. Molecular typing and antibiotic resistance patterns among clinical isolates of Acinetobacter baumannii recovered from burn patients in Tehran, Iran. Frontiers in microbiology. 2022;13:994303.

3. Mohamed AH, Sheikh Omar NM, Osman MM, Mohamud HA, Eraslan A, Gur M. Antimicrobial resistance and predisposing factors associated with catheter-associated UTI caused by uropathogens exhibiting multidrug-resistant patterns: a 3-year retrospective study at a tertiary Hospital in Mogadishu, Somalia. Tropical Medicine and Infectious Disease. 2022;7(3):42.

4. Bazaid AS, Barnawi H, Qanash H, Alsaif G, Aldarhami A, Gattan H, et al. Bacterial coinfection and antibiotic resistance profiles among hospitalised COVID-19 patients. Microorganisms. 2022;10(3):495.

5. Abdulzahra AT, Khalil MAF, Elkhatib WF. First report of colistin resistance among carbapenem-resistant Acinetobacter baumannii isolates recovered from hospitalized patients in Egypt. New Microbes New Infect. 2018;26:53-8.

6. Shenkutie AM, Yao MZ, Siu GK-h, Wong BKC, Leung PH-mJA. Biofilm-induced antibiotic resistance in clinical Acinetobacter baumannii isolates. 2020;9(11):817.

7. Vahhabi A, Hasani A, Rezaee MA, Baradaran B, Hasani A, Kafil HS, et al. Carbapenem resistance in Acinetobacter baumannii clinical isolates from northwest Iran: high prevalence of OXA genes in sync. Iranian Journal of Microbiology. 2021;13(3):282.

8. Kaya IA, Guner MD, Akca G, Tuncbilek S, Alhan A, Tekeli E. Evaluation of the synergistic effect of a combination of colistin and tigecycline against multidrug-resistant Acinetobacterbaumannii. Pak J Med Sci. 2017;33(2):393-7.

9. Valcek A, Nesporova K, Whiteway C, De Pooter T, De Coster W, Strazisar M, et al. Genomic analysis of a strain collection containing multidrug-, extensively drug-, pandrug-, and carbapenem-resistant modern clinical isolates of Acinetobacter baumannii. Antimicrobial Agents and Chemotherapy. 2022;66(9):e00892-22.

10. Agarwal S, Kakati B, Khanduri S, Gupta S. Emergence of Carbapenem Resistant Non-Fermenting Gram-Negative Bacilli Isolated in an ICU of a Tertiary Care Hospital. J Clin Diagn Res. 2017;11(1):DC04-DC7.

11. Agarwal S, Kakati B, Kishore N, Khanduri S, Singh M. Colistin resistance in organisms causing ventilator-associated pneumonia-Are we going into pre-antibiotic era? Critical Care & Shock. 2018;21(2).

12. Farajzadeh Sheikh A, Savari M, Abbasi Montazeri E, Khoshnood SJP, health g. Genotyping and molecular characterization of clinical Acinetobacter baumannii isolates from a single hospital in Southwestern Iran. 2020;114(5):251-61.

13. Asaad AM, Ansari S, Ajlan SE, Awad SMJI, resistance d. Epidemiology of biofilm producing Acinetobacter baumannii nosocomial isolates from a tertiary care hospital in Egypt: a cross-sectional study. 2021:709-17.

14. Akın F, Yazar A, Doğan M. Determining the infectious pathogens and their resistance to antibiotics in a pediatric intensive care unit. Journal of Pediatric Infectious Diseases. 2018;13(01):042-5.

15. Al Bshabshe A, Joseph MR, Al Hussein A, Haimour W, Hamid ME. Multidrug resistance Acinetobacter species at the intensive care unit, Aseer Central Hospital, Saudi Arabia: A one year analysis. Asian Pacific journal of tropical medicine. 2016;9(9):903-8.

16. Al Samawi MS, Khan FY, Eldeeb Y, Almaslamani M, Alkhal A, Alsoub H, et al. Acinetobacter Infections among Adult Patients in Qatar: A 2-Year Hospital-Based Study. Can J Infect Dis Med Microbiol. 2016;2016:6873689.

17. Abouelfetouh A, Torky AS, Aboulmagd EJB. Role of plasmid carrying bla NDM in mediating antibiotic resistance among Acinetobacter baumannii clinical isolates from Egypt. 2020;10(4):170.

18. Ayibieke A, Kobayashi A, Suzuki M, Sato W, Mahazu S, Prah I, et al. Prevalence and Characterization of Carbapenem-Hydrolyzing Class D β-Lactamase-Producing Acinetobacter Isolates From Ghana. 2020;11.

19. Aljindan R, Bukharie H, Alomar A, Abdalhamid B. Prevalence of digestive tract colonization of carbapenem-resistant Acinetobacter baumannii in hospitals in Saudi Arabia. J Med Microbiol. 2015;64(Pt 4):400-6.

20. Aljindan R, Elhadi N. Genetic relationship of multi-resistant acinetobacter baumannii isolates in kingdom of Saudi Arabia. J Pure Appl Microbiol. 2018;12(4):1951-8.

21. Bandy A, Almaeen AHJPo. Pathogenic spectrum of blood stream infections and resistance pattern in Gram-negative bacteria from Aljouf region of Saudi Arabia. 2020;15(6):e0233704.

22. Bandy A, Wani FA, Mohammed AH, Dar UF, Dar MR, Tantry BA. Bacteriological profile of wound infections and antimicrobial resistance in selected gram-negative bacteria. African Health Sciences. 2022;4(4):576-86.

23. Altun HU, Yagci S, Bulut C, Sahin H, Kinikli S, Adiloglu AK, et al. Antimicrobial Susceptibilities of Clinical Acinetobacter baumannii Isolates With Different Genotypes. Jundishapur J Microbiol. 2014;7(12):e13347.

24. Lavrinenko A, Sheck E, Kolesnichenko S, Azizov I, Turmukhambetova A. Antibiotic resistance and genotypes of nosocomial strains of Acinetobacter baumannii in Kazakhstan. Antibiotics. 2021;10(4):382.

25. Lukić-Grlić A, Kos M, Žižek M, Luxner J, Grisold A, Zarfel G, et al. Emergence of carbapenem-hydrolyzing oxacillinases in Acinetobacter baumannii in children from croatia. 2020;64(4):167-72.

26. Adjei AY, Vasaikar SD, Apalata T, Okuthe EG, Songca SP. Phylogenetic analysis of carbapenem-resistant Acinetobacter baumannii isolated from different sources using Multilocus Sequence Typing Scheme. Infection, Genetics and Evolution. 2021;96:105132.

27. Guzek A, Rybicki Z, Woźniak-Kosek A, Tomaszewski D. Bloodstream infections in the intensive care unit: A single-center retrospective bacteriological analysis between 2007 and 2019. Polish Journal of Microbiology. 2022;71(2):263-77.

28. Varghese A, Udayalaxmi J, Rao P, Suman EJJoP, Microbiology A. Blood Stream Infections caused by Non-Fermenting Gram Negative Bacilli, Clinical Correlation, MIC for Colistin, Gene Detection. 2020;14(3).

29. Gunalan A, Sarumathi D, Sastry AS, Ramanathan V, Rajaa S, Sistla S. Effect of combined colistin and meropenem against meropenem resistant Acinetobacter baumannii and Pseudomonas aeruginosa by checkerboard method: A cross sectional analytical study. Indian Journal of Pharmacology. 2021;53(3):207.

30. Szczypta A, Talaga-Ćwiertnia K, Kielar M, Krzyściak P, Gajewska A, Szura M, et al. Investigation of acinetobacter baumannii activity in vascular surgery units through epidemiological management based on the analysis of antimicrobial resistance, biofilm formation and genotyping. International Journal of Environmental Research and Public Health. 2021;18(4):1563.

31. Abou Fayad AG, Haraoui L-P, Sleiman A, Jaafar M, Zorgani A, Matar GM, et al. Diversity of Sequence Types and Impact of Fitness Cost among Carbapenem-Resistant Acinetobacter baumannii Isolates from Tripoli, Libya. Antimicrobial Agents and Chemotherapy. 2021;65(8):10.1128/aac. 00277-21.

32. Armengol E, Domenech O, Fusté E, Pérez-Guillén I, Borrell J, Sierra J, et al. Efficacy of combinations of colistin with other antimicrobials involves membrane fluidity and efflux machinery. Infection and Drug Resistance. 2019:2031-8.

33. Arzu KAYIŞ MA. Molecular Epidemiological Evaluation of

Acinetobacter baumannii Isolates Isolated As the

Agent of Hospital Infections in Türkiye. FLORA. 2022;27 ( 2 ):227 - 40

34. Asaad AM, Al-Ayed MSZ, Qureshi MA. Emergence of unusual nonfermenting gram-negative nosocomial pathogens in a Saudi hospital. Japanese Journal of Infectious Diseases. 2013;66(6):507-11.

35. Athanassa ZE, Markantonis SL, Fousteri M-ZF, Myrianthefs PM, Boutzouka EG, Tsakris A, et al. Pharmacokinetics of inhaled colistimethate sodium (CMS) in mechanically ventilated critically ill patients. Intensive care medicine. 2012;38:1779-86.

36. Aydın M, Ergönül Ö, Azap A, Bilgin H, Aydın G, Çavuş SA, et al. Rapid emergence of colistin resistance and its impact on fatality among healthcare-associated infections. J Hosp Infect. 2018;98(3):260-3.

37. Guclu AU, Kocak AA, Ok MA, Tutluoglu B, Basustaoglu AC, Group RS. Antibacterial resistance in lower respiratory tract bacterial pathogens: A multicenter analysis from turkey. The Journal of Infection in Developing Countries. 2021;15(02):254-62.

38. Mabrouk A, Chebbi Y, Raddaoui A, Krir A, Messadi AA, Achour W, et al. Clonal spread of PER-1 and OXA-23 producing extensively drug resistant Acinetobacter baumannii during an outbreak in a burn intensive care unit in Tunisia. 2020;67(4):222-7.

39. Bado I, Papa-Ezdra R, Delgado-Blas JF, Gaudio M, Gutiérrez C, Cordeiro NF, et al. Molecular characterization of carbapenem-resistant Acinetobacter baumannii in the intensive care unit of Uruguay's University Hospital identifies the first rmtC gene in the species. Microbial Drug Resistance. 2018;24(7):1012-9.

40. Bagheri-Nesami M, Rezai MS, Ahangarkani F, Rafiei A, Nikkhah A, Eslami G, et al. Multidrug and co-resistance patterns of non-fermenting Gram-negative bacilli involved in ventilator-associated pneumonia carrying class 1 integron in the North of Iran. Germs. 2017;7(3):123.

41. Bahador A, Farshadzadeh Z, Raoofian R, Mokhtaran M, Pourakbari B, Pourhajibagher M, et al. Association of virulence gene expression with colistin-resistance in Acinetobacter baumannii: analysis of genotype, antimicrobial susceptibility, and biofilm formation. Annals of Clinical Microbiology and Antimicrobials. 2018;17:1-12.

42. Bahador A, Raoo An R, Farshadzadeh Z, Beitollahi L, Khaledi A, Rahimi S, et al. The Prevalence of IS Aba 1 and IS Aba 4 in Acinetobacter baumannii Species of Different International Clone Lineages Among Patients With Burning in Tehran, Iran. Jundishapur J Microbiol. 2015;8(7):e17167.

43. Bahador A, Taheri M, Pourakbari B, Hashemizadeh Z, Rostami H, Mansoori N, et al. Emergence of rifampicin, tigecycline, and colistin-resistant Acinetobacter baumannii in Iran; spreading of MDR strains of novel International Clone variants. Microbial drug resistance. 2013;19(5):397-406.

44. Salehi B, Ghalavand Z, Yadegar A, Eslami G. Characteristics and diversity of mutations in regulatory genes of resistance-nodulation-cell division efflux pumps in association with drug-resistant clinical isolates of Acinetobacter baumannii. Antimicrobial Resistance & Infection Control. 2021;10:1-12.

45. Khamari B, Lama M, Pachi Pulusu C, Biswal AP, Lingamallu SM, Mukkirla BS, et al. Molecular analyses of biofilm-producing clinical Acinetobacter baumannii isolates from a South Indian Tertiary Care Hospital. 2020;29(6):580-7.

46. Ballouz T, Aridi J, Afif C, Irani J, Lakis C, Nasreddine R, et al. Risk factors, clinical presentation, and outcome of Acinetobacter baumannii bacteremia. Frontiers in cellular and infection microbiology. 2017;7:156.

47. Bardbari AM, Arabestani MR, Karami M, Keramat F, Aghazadeh H, Alikhani MY, et al. Highly synergistic activity of melittin with imipenem and colistin in biofilm inhibition against multidrug-resistant strong biofilm producer strains of Acinetobacter baumannii. European Journal of Clinical Microbiology & Infectious Diseases. 2018;37:443-54.

48. Batarseh A, Al-Sarhan A, Maayteh M, Al-Khatirei S, Alarmouti M. Antibiogram of multidrug resistant Acinetobacter baumannii isolated from clinical specimens at King Hussein Medical Centre, Jordan: a retrospective analysis. East Mediterr Health J. 2016;21(11):828-34.

49. Bayram Y, Parlak M, Aypak C, Bayram İ. Three-year review of bacteriological profile and antibiogram of burn wound isolates in Van, Turkey. International journal of medical sciences. 2013;10(1):19.

50. Sobouti B, Mirshekar M, Fallah S, Tabaei A, Mehrabadi JF, Darbandi AJMjotIRoI. Pan drug-resistant Acinetobacter baumannii causing nosocomial infections among burnt children. 2020;34:24.

51. Balázs B, Tóth Z, Nagy JB, Majoros L, Tóth Á, Kardos G. Faecal carriage of carbapenem-resistant Acinetobacter baumannii: comparison to clinical isolates from the same period (2017–2019). Pathogens. 2022;11(9):1003.

52. Segatore B, Piccirilli A, Cherubini S, Principe L, Alloggia G, Mezzatesta ML, et al. In Vitro Activity of Sulbactam–Durlobactam against Carbapenem-Resistant Acinetobacter baumannii Clinical Isolates: A Multicentre Report from Italy. Antibiotics. 2022;11(8):1136.

53. Santella B, Serretiello E, De Filippis A, Folliero V, Iervolino D, Dell’Annunziata F, et al. Lower respiratory tract pathogens and their antimicrobial susceptibility pattern: a 5-year study. 2021;10(7):851.

54. Liu B, Liu Y, Di X, Zhang X, Wang R, Bai Y, et al. Colistin and anti-Gram-positive bacterial agents against Acinetobacter baumannii. Revista da Sociedade Brasileira de Medicina Tropical. 2014;47:451-6.

55. Bogdan M, Drenjancevic D, Harsanji Drenjancevic I, Bedenic B, Zujic Atalic V, Talapko J, et al. In vitro effect of subminimal inhibitory concentrations of antibiotics on the biofilm formation ability of Acinetobacter baumannii clinical isolates. Journal of Chemotherapy. 2018;30(1):16-24.

56. Lukovic B, Gajic I, Dimkic I, Kekic D, Zornic S, Pozder T, et al. The first nationwide multicenter study of Acinetobacter baumannii recovered in Serbia: emergence of OXA-72, OXA-23 and NDM-1-producing isolates. 2020;9:1-12.

57. Boral B, Unaldi Ö, Ergin A, Durmaz R, Eser ÖK. A prospective multicenter study on the evaluation of antimicrobial resistance and molecular epidemiology of multidrug-resistant Acinetobacter baumannii infections in intensive care units with clinical and environmental features. Annals of clinical microbiology and antimicrobials. 2019;18(1):1-9.

58. Bozkurt-Guzel C, Savage PB, Akcali A, Ozbek-Celik B. Potential synergy activity of the novel ceragenin, CSA-13, against carbapenem-resistant Acinetobacter baumannii strains isolated from bacteremia patients. BioMed research international. 2014;2014.

59. Bedenić B, Likić S, Žižek M, Bratić V, D'Onofrio V, Cavrić G, et al. Causative agents of bloodstream infections in two Croatian hospitals and their resistance mechanisms. Journal of Chemotherapy. 2023;35(4):281-91.

60. Bratić V, Mihaljević S, Verzak Ž, Pleško E, Lukić A, Ćaćić M, et al. Prophylactic application of antibiotics selects extended‐spectrum β‐lactamase and carbapenemases producing Gram‐negative bacteria in the oral cavity. Letters in Applied Microbiology. 2021;73(2):206-19.

61. Konca C, Tekin M, Geyik MJTIJoP. Susceptibility patterns of multidrug-resistant Acinetobacter baumannii. 2021;88:120-6.

62. Camargo CH, Cunha MPV, de Barcellos TAF, Bueno MS, de Jesus Bertani AM, Dos Santos CA, et al. Genomic and phenotypic characterisation of antimicrobial resistance in carbapenem-resistant Acinetobacter baumannii hyperendemic clones CC1, CC15, CC79 and CC25. 2020;56(6):106195.

63. Nodari CS, Cayô R, Streling AP, Lei F, Wille J, Almeida MS, et al. Genomic analysis of carbapenem-resistant Acinetobacter baumannii isolates belonging to major endemic clones in South America. 2020;11:584603.

64. Carretto E, Barbarini D, Dijkshoorn L, van der Reijden T, Brisse S, Passet V, et al. Widespread carbapenem resistant Acinetobacter baumannii clones in Italian hospitals revealed by a multicenter study. Infection, Genetics and Evolution. 2011;11(6):1319-26.

65. Özkul C, Hazırolan G. Oxacillinase gene distribution, antibiotic resistance, and their correlation with biofilm formation in Acinetobacter baumannii bloodstream isolates. Microbial Drug Resistance. 2021;27(5):637-46.

66. Chaari A, Mnif B, Bahloul M, Mahjoubi F, Chtara K, Turki O, et al. Acinetobacter baumannii ventilator-associated pneumonia: epidemiology, clinical characteristics, and prognosis factors. International Journal of infectious diseases. 2013;17(12):e1225-e8.

67. Longshaw C, Manissero D, Tsuji M, Echols R, Yamano YJJ-AR. In vitro activity of the siderophore cephalosporin, cefiderocol, against molecularly characterized, carbapenem-non-susceptible Gram-negative bacteria from Europe. 2020;2(3):dlaa060.

68. Lin C-H, Lee M-C, Tzen JT, Lee H-M, Chang S-M, Tu W-C, et al. Efficacy of Mastoparan-AF alone and in combination with clinically used antibiotics on nosocomial multidrug-resistant Acinetobacter baumannii. Saudi journal of biological sciences. 2017;24(5):1023-9.

69. Coelho-Souza T, Reis JN, Martins N, Martins IS, Menezes AO, Reis MG, et al. Longitudinal surveillance for meningitis by Acinetobacter in a large urban setting in Brazil. Clin Microbiol Infect. 2013;19(5):E241-4.

70. Liu C, Chen K, Wu Y, Huang L, Fang Y, Lu J, et al. Epidemiological and genetic characteristics of clinical carbapenem-resistant Acinetobacter baumannii strains collected countrywide from hospital intensive care units (ICUs) in China. Emerging Microbes & Infections. 2022;11(1):1730-41.

71. Custovic A, Smajlovic J, Tihic N, Hadzic S, Ahmetagic S, Hadzagic H. Epidemiological monitoring of nosocomial infections caused by Acinetobacter baumannii. Medical Archives. 2014;68(6):402.

72. D'Arezzo S, Principe L, Capone A, Petrosillo N, Petrucca A, Visca P. Changing carbapenemase gene pattern in an epidemic multidrug-resistant Acinetobacter baumannii lineage causing multiple outbreaks in central Italy. Journal of Antimicrobial Chemotherapy. 2011;66(1):54-61.

73. Kim DH, Jung S-I, Kwon KT, Ko KS. Occurrence of diverse AbGRI1-type genomic islands in Acinetobacter baumannii global clone 2 isolates from South Korea. Antimicrobial agents and chemotherapy. 2017;61(2):10.1128/aac. 01972-16.

74. Dafopoulou K, Tsakris A, Pournaras S. Changes in antimicrobial resistance of clinical isolates of Acinetobacter baumannii group isolated in Greece, 2010-2015. J Med Microbiol. 2018;67(4):496-8.

75. Depka D, Bogiel T, Rzepka M, Gospodarek-Komkowska E. The Prevalence of Virulence Factor Genes among Carbapenem-Non-Susceptible Acinetobacter baumannii Clinical Strains and Their Usefulness as Potential Molecular Biomarkers of Infection. Diagnostics. 2023;13(6):1036.

76. Bandić-Pavlović D, Zah-Bogović T, Žižek M, Bielen L, Bratić V, Hrabač P, et al. Gram-negative bacteria as causative agents of ventilator-associated pneumonia and their respective resistance mechanisms. 2020;32(7):344-58.

77. De Vos D, Pirnay JP, Bilocq F, Jennes S, Verbeken G, Rose T, et al. Molecular Epidemiology and Clinical Impact of Acinetobacter calcoaceticus-baumannii Complex in a Belgian Burn Wound Center. PLoS One. 2016;11(5):e0156237.

78. Vandresen DF, Lucio LC, Yamada RS, Vieira AP, Follador FAC, Benedetti VP, et al. Associated factors of Acinetobacter baumannii complex in hospitalized patients: A case-control study. The Journal of Infection in Developing Countries. 2021;15(01):73-80.

79. Jalal D, Elzayat MG, Diab AA, El-Shqanqery HE, Samir O, Bakry U, et al. Deciphering multidrug-resistant Acinetobacter baumannii from a pediatric cancer hospital in Egypt. Msphere. 2021;6(6):e00725-21.

80. Gkentzi D, Tsintoni A, Christopoulou I, Mamalis I, Paliogianni F, Assimakopoulos SF, et al. Extensively-drug resistant Acinetobacter baumannii bacteremia in neonates: effective treatment with the combination of colistin and ampicillin/sulbactam. 2020;32(2):103-6.

81. Di Domenico EG, Farulla I, Prignano G, Gallo MT, Vespaziani M, Cavallo I, et al. Biofilm is a Major Virulence Determinant in Bacterial Colonization of Chronic Skin Ulcers Independently from the Multidrug Resistant Phenotype. Int J Mol Sci. 2017;18(5).

82. Alrahmany D, Omar AF, Harb G, El Nekidy WS, Ghazi IMJA. Acinetobacter baumannii infections in hospitalized patients, treatment outcomes. 2021;10(6):630.

83. Dias VC, Resende JA, Bastos AN, De Andrade Bastos LQ, De Andrade Bastos VQ, Bastos RV, et al. Epidemiological, Physiological, and Molecular Characteristics of a Brazilian Collection of Carbapenem-Resistant Acinetobacter baumannii and Pseudomonas aeruginosa. Microb Drug Resist. 2017;23(7):852-63.

84. Petropoulou D, Siopi M, Vourli S, Pournaras S. Activity of sulbactam-durlobactam and comparators against a national collection of carbapenem-resistant Acinetobacter baumannii isolates from Greece. Frontiers in Cellular and Infection Microbiology. 2022;11:1458.

85. Taušan Ð, Rančić N, Kostić Z, Ljubenović N, Rakonjac B, Šuljagić V. An assessment of burden of hospital-acquired pneumonia among abdominal surgical patients in tertiary university hospital in Serbia: A matched nested case-control study. Frontiers in Medicine. 2022;9:1040654.

86. Doymaz MZ, Karaaslan E. Comparison of antibacterial activities of polymyxin B and colistin against multidrug resistant Gram negative bacteria. Infect Dis (Lond). 2019;51(9):676-82.

87. Duarte A, Ferreira S, Almeida S, Domingues FC. Clinical isolates of Acinetobacter baumannii from a Portuguese hospital: PFGE characterization, antibiotic susceptibility and biofilm-forming ability. Comp Immunol Microbiol Infect Dis. 2016;45:29-33.

88. Ho DS, Dinh HC, Le TD, Ho DT, Ngo HT, Nguyen CD. Sputum culture and antibiotic resistance in elderly inpatients with exacerbation of chronic obstructive pulmonary disease at a tertiary geriatric hospital in southern Vietnam. Aging Clinical and Experimental Research. 2023;35(6):1347-56.

89. Diep DTH, Tuan HM, Ngoc KM, Vinh C, Dung TTN, Phat VV, et al. The clinical features and genomic epidemiology of carbapenem-resistant Acinetobacter baumannii infections at a tertiary hospital in Vietnam. Journal of Global Antimicrobial Resistance. 2023;33:267-75.

90. Durdu B, Kritsotakis EI, Lee ACK, Torun P, Hakyemez IN, Gultepe B, et al. Temporal trends and patterns in antimicrobial-resistant Gram-negative bacteria implicated in intensive care unit-acquired infections: A cohort-based surveillance study in Istanbul, Turkey. J Glob Antimicrob Resist. 2018;14:190-6.

91. Çağlan E, Nigiz Ş, Sancak B, Gür DJAmeiH. Resistance and heteroresistance to colistin among clinical isolates of Acinetobacter baumannii. 2020;67(2):107-11.

92. Sharifipour E, Shams S, Esmkhani M, Khodadadi J, Fotouhi-Ardakani R, Koohpaei A, et al. Evaluation of bacterial co-infections of the respiratory tract in COVID-19 patients admitted to ICU. 2020;20(1):1-7.

93. Buzilă ER, Năstase EV, Luncă C, Bădescu A, Miftode E, Iancu LS. Antibiotic resistance of non-fermenting Gram-negative bacilli isolated at a large Infectious Diseases Hospital in North-Eastern Romania, during an 11-year period. Germs. 2021;11(3):354.

94. Abbasi E, Goudarzi H, Hashemi A, Chirani AS, Ardebili A, Goudarzi M, et al. Decreased carO gene expression and OXA-type carbapenemases among extensively drug-resistant Acinetobacter baumannii strains isolated from burn patients in Tehran, Iran. Acta Microbiologica et Immunologica Hungarica. 2021;68(1):48-54.

95. Castagnola E, Bagnasco F, Mesini A, Agyeman P, Ammann R, Carlesse F, et al. Antibiotic Resistant Bloodstream Infections in Pediatric Patients Receiving Chemotherapy or Hematopoietic Stem Cell Transplant: Factors Associated with Development of Resistance, Intensive Care Admission and Mortality. Antibiotics (Basel) 2021; 10: 1–14.

96. El-Shazly S, Dashti A, Vali L, Bolaris M, Ibrahim AS. Molecular epidemiology and characterization of multiple drug-resistant (MDR) clinical isolates of Acinetobacter baumannii. Int J Infect Dis. 2015;41:42-9.

97. Cercenado E, Cardenoso L, Penin R, Longshaw C, Henriksen AS, Pascual A. In vitro activity of cefiderocol and comparators against isolates of Gram-negative bacterial pathogens from a range of infection sources: SIDERO‑WT‑2014− 2018 studies in Spain. Journal of Global Antimicrobial Resistance. 2021;26:292-300.

98. Shenoy ES, Pierce VM, Sater MR, Pangestu FK, Herriott IC, Bramante JT, et al. Community-acquired in name only: a cluster of carbapenem-resistant Acinetobacter baumannii in a burn intensive care unit and beyond. 2020;41(5):531-8.

99. Yusuf E, Tompa M, Strepis N, Klaassen CH, Goessens WH. High Prevalence of ST502 Carrying an OXA-24 Carbapenemase gene in Carbapenem-Nonsusceptible Acinetobacter baumannii-calcoaceticus Isolates in Romania. Microbial Drug Resistance. 2022;28(6):636-44.

100. Esposito S, Pascale R, Esposito I, Noviello S, Russo E, Simone GD, et al. Epidemiology and antibiotic resistance in a large Italian teaching hospital. J Chemother. 2015;27(3):163-6.

101. Esposito S, Gioia R, De Simone G, Noviello S, Lombardi D, Di Crescenzo VG, et al. Bacterial epidemiology and antimicrobial resistance in the surgery wards of a large teaching hospital in Southern Italy. Mediterranean Journal of Hematology and Infectious Diseases. 2015;7(1).

102. AKKAN KUZUCU E, ÇALIŞKAN, E., ÖCAL, D., DANSUK, Z., ÇAĞATAY, M., ERDEM, Ü. G., ... TEKELİ, F. A. . Investigation of Colistin Resistance and Heteroresistance in Acinetobacter spp. Isolates From Various Clinical Specimens. . Mediterranean Journal of Infection, Microbes and Antimicrobials 2022.

103. Falagas ME, Skalidis T, Vardakas KZ, Voulgaris GL, Papanikolaou G, Legakis N. Activity of TP-6076 against carbapenem-resistant Acinetobacter baumannii isolates collected from inpatients in Greek hospitals. Int J Antimicrob Agents. 2018;52(2):269-71.

104. Bakhshi F, Firoozeh F, Badmasti F, Dadashi M, Zibaei M, Khaledi A. Molecular Detection of OXA-type Carbapenemases among Isolated from Burn Patients and Hospital Environments. The Open Microbiology Journal. 2022;16(1).

105. Sana F, Hussain A, Hussain W, Zaman G, Abbas MW, Imtiaz A, et al. Frequency and clinical spectrum of multidrug resistant acinetobacter baumannii as a significant nosocomial pathogen in intensive care unit patients. J Ayub Med Coll Abbottabad. 2021;33(Suppl 1):752-6.

106. Jabeen F, Khan Z, Sohail M, Tahir A, Tipu I, Murtaza Saleem HG. Antibiotic Resistance Pattern Of Acinetobacter Baumannii Isolated From Bacteremia Patients In Pakistan. Journal of Ayub Medical College Abbottabad-Pakistan. 2022;34(1).

107. Sacco F, Visca P, Runci F, Antonelli G, Raponi GJA. Susceptibility Testing of Colistin for Acinetobacter baumannii: How Far Are We from the Truth? 2021;10(1):48.

108. Ezadi F, Jamali A, Heidari A, Javid N, Ardebili AJJoGAR. Heteroresistance to colistin in oxacillinase-producing carbapenem-resistant Acinetobacter baumannii clinical isolates from Gorgan, Northern Iran. 2020;21:380-5.

109. Pasteran F, Danze D, Menocal A, Cabrera C, Castillo I, Albornoz E, et al. Simple phenotypic tests to improve accuracy in screening chromosomal and plasmid-mediated colistin resistance in Gram-negative bacilli. 2020;59(1):10.1128/jcm. 01701-20.

110. Feretzakis G, Loupelis E, Sakagianni A, Skarmoutsou N, Michelidou S, Velentza A, et al. A 2-Year Single-Centre Audit on Antibiotic Resistance of Pseudomonas aeruginosa, Acinetobacter baumannii and Klebsiella pneumoniae Strains from an Intensive Care Unit and Other Wards in a General Public Hospital in Greece. Antibiotics (Basel). 2019;8(2).

111. Flamm RK, Rhomberg PR, Jones RN, Farrell DJ. In vitro activity of RX-P873 against Enterobacteriaceae, Pseudomonas aeruginosa, and Acinetobacter baumannii. Antimicrob Agents Chemother. 2015;59(4):2280-5.

112. Flamm RK, Farrell DJ, Sader HS, Jones RN. Ceftazidime/avibactam activity tested against Gram-negative bacteria isolated from bloodstream, pneumonia, intra-abdominal and urinary tract infections in US medical centres (2012). J Antimicrob Chemother. 2014;69(6):1589-98.

113. Alvares FA, de Oliveira CS, Alves DCI, Braun GJRdEeCdI. Ventilator-associated pneumonia: incidence, microbial etiology and antimicrobial resistance profile. 2021;11(4):01-8.

114. Fu Y, Zhou J, Zhou H, Yang Q, Wei Z, Yu Y, et al. Wide dissemination of OXA-23-producing carbapenem-resistant Acinetobacter baumannii clonal complex 22 in multiple cities of China. J Antimicrob Chemother. 2010;65(4):644-50.

115. Bianco G, Boattini M, Comini S, Iannaccone M, Casale R, Allizond V, et al. Activity of ceftolozane-tazobactam, ceftazidime-avibactam, meropenem-vaborbactam, cefiderocol and comparators against Gram-negative organisms causing bloodstream infections in Northern Italy (2019–2021): Emergence of complex resistance phenotypes. Journal of Chemotherapy. 2022;34(5):302-10.

116. Wareth G, Linde J, Nguyen NH, Nguyen TN, Sprague LD, Pletz MW, et al. WGS-based analysis of carbapenem-resistant Acinetobacter baumannii in Vietnam and molecular characterization of antimicrobial determinants and MLST in Southeast Asia. Antibiotics. 2021;10(5):563.

117. Gazel D, Otkun M. Investigation of Colistin Heteroresistance and Some Factors Affecting Heteroresistance in Carbapenem-Resistant A. baumannii Strains. Mediterranean Journal of Infection Microbes and Antimicrobials. 2018.

118. Papathanakos G, Andrianopoulos I, Papathanasiou A, Priavali E, Koulenti D, Koulouras VJM. Colistin-resistant Acinetobacter baumannii bacteremia: a serious threat for critically ill patients. 2020;8(2):287.

119. Ghaith DM, Zafer MM, Al-Agamy MH, Alyamani EJ, Booq RY, Almoazzamy O. The emergence of a novel sequence type of MDR Acinetobacter baumannii from the intensive care unit of an Egyptian tertiary care hospital. Ann Clin Microbiol Antimicrob. 2017;16(1):34.

120. Gholami M, Moshiri M, Ahanjan M, Salimi Chirani A, Hasannejad-Bibalan M, Asadi A, et al. The diversity of class B and class D carbapenemases in clinical Acinetobacter baumannii isolates. Le infezioni in medicina : rivista periodica di eziologia, epidemiologia, diagnostica, clinica e terapia delle patologie infettive. 2018;26:329-35.

121. Giamarellos-Bourboulis EJ, Xirouchaki E, Giamarellou H. Interactions of colistin and rifampin on multidrug-resistant Acinetobacter baumannii. Diagn Microbiol Infect Dis. 2001;40(3):117-20.

122. Giannouli M, Di Popolo A, Durante-Mangoni E, Bernardo M, Cuccurullo S, Amato G, et al. Molecular epidemiology and mechanisms of rifampicin resistance in Acinetobacter baumannii isolates from Italy. Int J Antimicrob Agents. 2012;39(1):58-63.

123. Montrucchio G, Corcione S, Lupia T, Shbaklo N, Olivieri C, Poggioli M, et al. The burden of carbapenem-resistant Acinetobacter baumannii in ICU COVID-19 patients: a regional experience. Journal of clinical medicine. 2022;11(17):5208.

124. Goudarzi H, Azad M, Seyedjavadi SS, Azimi H, Salimi Chirani A, Fallah Omrani V, et al. Characterization of integrons and associated gene cassettes in Acinetobacter baumannii strains isolated from intensive care unit in Tehran, Iran. Journal of Acute Disease. 2016;5(5):386-92.

125. Guzek A, Korzeniewski K, Tomaszewski D, Rybicki Z, Zwolinska E. Bacteriological Assessment of Pneumonia Caused by Gram-Negative Bacteria in Patients Hospitalized in Intensive Care Unit. Adv Exp Med Biol. 2017;955:39-46.

126. Guzek A, Rybicki Z, Korzeniewski K, Mackiewicz K, Saks E, Chciałowski A, et al. Etiological Factors Causing Lower Respiratory Tract Infections Isolated from Hospitalized Patients. In: Pokorski M, editor. Respiratory Infections. Cham: Springer International Publishing; 2015. p. 37-44.

127. Hackel MA, Tsuji M, Yamano Y, Echols R, Karlowsky JA, Sahm DF. In Vitro Activity of the Siderophore Cephalosporin, Cefiderocol, against Carbapenem-Nonsusceptible and Multidrug-Resistant Isolates of Gram-Negative Bacilli Collected Worldwide in 2014 to 2016. Antimicrob Agents Chemother. 2018;62(2).

128. Kon H, Abramov S, Amar Ben Dalak M, Elmaliach N, Schwartz D, Carmeli Y, et al. Performance of Rapid Polymyxin™ NP and Rapid Polymyxin™ Acinetobacter for the detection of polymyxin resistance in carbapenem-resistant Acinetobacter baumannii and Enterobacterales. 2020;75(6):1484-90.

129. Necati Hakyemez I, Kucukbayrak A, Tas T, Burcu Yikilgan A, Akkaya A, Yasayacak A, et al. Nosocomial Acinetobacter baumannii Infections and Changing Antibiotic Resistance. Pak J Med Sci. 2013;29(5):1245-8.

130. Seifert H, Stefanik D, Olesky M, Higgins PGJIjoaa. In vitro activity of the novel fluorocycline TP-6076 against carbapenem-resistant Acinetobacter baumannii. 2020;55(1):105829.

131. Seifert H, Blondeau J, Lucassen K, Utt EA. Global update on the in vitro activity of tigecycline and comparators against isolates of Acinetobacter baumannii and rates of resistant phenotypes (2016–2018). Journal of Global Antimicrobial Resistance. 2022;31:82-9.

132. Ejaz H, Qamar MU, Junaid K, Younas S, Taj Z, Bukhari SNA, et al. The Molecular Detection of Class B and Class D Carbapenemases in Clinical Strains of Acinetobacter calcoaceticus-baumannii Complex: The High Burden of Antibiotic Resistance and the Co-Existence of Carbapenemase Genes. 2022;11(9):1168.

133. Ejaz H, Ahmad M, Younas S, Junaid K, Abosalif KOA, Abdalla AE, et al. Molecular epidemiology of extensively-drug resistant Acinetobacter baumannii sequence type 2 co-harboring bla NDM and bla OXA from clinical origin. 2021:1931-9.

134. Chen H, Wang Z, Li H, Wang Q, Zhao C, He W, et al. In Vitro Analysis of Activities of 16 Antimicrobial Agents against Gram-Negative Bacteria from Six Teaching Hospitals in China. Jpn J Infect Dis. 2015;68(4):263-7.

135. Özçelik HB, Yildirim T, Marakli S, Idil Ö. Investigation of oxacillinases type beta-lactamases in carbapenems resistant Acinetobacter baumannii clinical isolates. Reviews and Research in Medical Microbiology. 2020;31(4):209-14.

136. Zhang H, Zhang G, Zhang J, Duan S, Kang Y, Yang Q, et al. Antimicrobial Activity of Colistin Against Contemporary (2015–2017) P. aeruginosa and A. baumannii Isolates From a Chinese Surveillance Program. Frontiers in Microbiology. 2020;11:1966.

137. Zhang H, Jia P, Zhu Y, Zhang G, Zhang J, Kang W, et al. Susceptibility to imipenem/relebactam of Pseudomonas aeruginosa and acinetobacter baumannii isolates from Chinese intra-abdominal, respiratory and urinary tract infections: SMART 2015 to 2018. Infection and Drug Resistance. 2021:3509-18.

138. Son H-J, Cho EB, Bae M, Lee SC, Sung H, Kim M-N, et al., editors. Clinical and microbiological analysis of risk factors for mortality in patients with carbapenem-resistant Acinetobacter baumannii bacteremia. Open Forum Infectious Diseases; 2020: Oxford University Press US.

139. Kim HA, Ryu SY, Seo I, Suh SI, Suh MH, Baek WK. Biofilm Formation and Colistin Susceptibility of Acinetobacter baumannii Isolated from Korean Nosocomial Samples. Microb Drug Resist. 2015;21(4):452-7.

140. Kang HM, Yun KW, Choi EH. Molecular epidemiology of Acinetobacter baumannii complex causing invasive infections in Korean children during 2001–2020. Annals of Clinical Microbiology and Antimicrobials. 2023;22(1):1-9.

141. Morrissey I, Olesky M, Hawser S, Lob SH, Karlowsky JA, Corey GR, et al. In vitro activity of eravacycline against Gram-negative bacilli isolated in clinical laboratories worldwide from 2013 to 2017. Antimicrobial agents and chemotherapy. 2020;64(3):10.1128/aac. 01699-19.

142. Gajic I, Ranin L, Kekic D, Opavski N, Smitran A, Mijac V, et al. Tigecycline susceptibility of multidrug-resistant Acinetobacter baumannii from intensive care units in the western Balkans. Acta Microbiologica et Immunologica Hungarica. 2020;67(3):176-81.

143. İpek MS, Aktar F, Okur N, Celik M, Ozbek E. Colistin use in critically ill neonates: A case-control study. Pediatr Neonatol. 2017;58(6):490-6.

144. Mumcuoğlu İ, Çağlar H, Erdem D, Aypak A, Gün P, Kurşun Ş, et al. Secondary bacterial infections of the respiratory tract in COVID-19 patients. The Journal of Infection in Developing Countries. 2022;16(07):1131-7.

145. Gheorghe I, Barbu IC, Surleac M, Sârbu I, Popa LI, Paraschiv S, et al. Subtypes, resistance and virulence platforms in extended-drug resistant Acinetobacter baumannii Romanian isolates. Scientific Reports. 2021;11(1):13288.

146. Al-Kadmy IM, Ibrahim SA, Al-Saryi N, Aziz SN, Besinis A, Hetta HF. Prevalence of genes involved in colistin resistance in Acinetobacter baumannii: first report from Iraq. Microbial Drug Resistance. 2020;26(6):616-22.

147. Abdul-Mutakabbir JC, Yim J, Nguyen L, Maassen PT, Stamper K, Shiekh Z, et al. In vitro synergy of colistin in combination with meropenem or tigecycline against carbapenem-resistant Acinetobacter baumannii. Antibiotics. 2021;10(7):880.

148. Jain M, Sharma A, Sen MK, Rani V, Gaind R, Suri JC. Phenotypic and molecular characterization of Acinetobacter baumannii isolates causing lower respiratory infections among ICU patients. Microb Pathog. 2019;128:75-81.

149. Karlowsky JA, Hackel MA, McLeod SM, Miller AA. In vitro activity of sulbactam-durlobactam against global isolates of Acinetobacter baumannii-calcoaceticus complex collected from 2016 to 2021. Antimicrobial Agents and Chemotherapy. 2022;66(9):e00781-22.

150. Hrbacek J, Cermak P, Zachoval R. Current antibiotic resistance patterns of rare uropathogens: survey from Central European Urology Department 2011–2019. BMC urology. 2021;21:1-8.

151. Houngsaitong J, Montakantikul P, Paiboonwong T, Chomnawang M, Khuntayaporn P, Chulavatnatol S. In vitro activity of biapenem and comparators against multidrug-resistant and carbapenem-resistant Acinetobacter baumannii isolated from tertiary care hospitals in Thailand. Pharmaceutical Sciences Asia. 2020;47(4).

152. Mancilla-Rojano J, Ochoa SA, Reyes-Grajeda JP, Flores V, Medina-Contreras O, Espinosa-Mazariego K, et al. Molecular epidemiology of acinetobacter calcoaceticus-acinetobacter baumannii complex isolated from children at the hospital Infantil de México Federico Gómez. Frontiers in Microbiology. 2020;11:576673.

153. Woon JJ, Teh CSJ, Chong CW, Abdul Jabar K, Ponnampalavanar S, Idris N. Molecular characterization of carbapenem-resistant Acinetobacter baumannii isolated from the intensive care unit in a tertiary teaching hospital in malaysia. Antibiotics. 2021;10(11):1340.

154. Lei J, Han S, Wu W, Wang X, Xu J, Han L. Extensively drug-resistant Acinetobacter baumannii outbreak cross-transmitted in an intensive care unit and respiratory intensive care unit. Am J Infect Control. 2016;44(11):1280-4.

155. Xi J, Jia P, Zhu Y, Yu W, Zhang J, Gao H, et al. Antimicrobial susceptibility to polymyxin B and other comparators against Gram-negative bacteria isolated from bloodstream infections in China: Results from CARVIS-NET program. Frontiers in Microbiology. 2022;13:1017488.

156. Choi JY, Ko EA, Kwon KT, Lee S, Kang CI, Chung DR, et al. Acinetobacter sp. isolates from emergency departments in two hospitals of South Korea. J Med Microbiol. 2014;63(Pt 10):1363-8.

157. Jones RN, Castanheira M, Hu B, Ni Y, Lin SS, Mendes RE, et al. Update of contemporary antimicrobial resistance rates across China: reference testing results for 12 medical centers (2011). Diagn Microbiol Infect Dis. 2013;77(3):258-66.

158. Li J, Yu T, Luo Y, Peng J-Y, Li Y-J, Tao X-Y, et al. Characterization of carbapenem-resistant hypervirulent Acinetobacter baumannii strains isolated from hospitalized patients in the mid-south region of China. BMC microbiology. 2020;20:1-8.

159. Qu J, Yu R, Wang Q, Feng C, Lv X. Synergistic antibacterial activity of combined antimicrobials and the clinical outcome of patients with carbapenemase-producing Acinetobacter baumannii infection. Frontiers in Microbiology. 2020;11:541423.

160. Chang KC, Lin MF, Lin NT, Wu WJ, Kuo HY, Lin TY, et al. Clonal spread of multidrug-resistant Acinetobacter baumannii in eastern Taiwan. J Microbiol Immunol Infect. 2012;45(1):37-42.

161. Ghaima KK, Saadedin SMK, Jassim KA. Prevalence of BIaOXA like Carbapenemase Genes in Multidrug Resistant Acinetobacter baumannii Isolated from burns and Wounds in Baghdad Hospitals. RESEARCH JOURNAL OF PHARMACEUTICAL BIOLOGICAL AND CHEMICAL SCIENCES. 2016;7(3):1247-54.

162. Kaliterna V, Kaliterna M, Hrenović J, Barišić Z, Tonkić M, Goic-Barisic I. Acinetobacter baumannii in Southern Croatia: clonal lineages, biofilm formation, and resistance patterns. Infectious Diseases. 2015;47(12):902-7.

163. Al Otraqchi KIB. Molecular Level Study and Characterization of Plasmid Deoxyribonucleic Acid Profile of Acinetobacter baumannii Isolated from Diabetic Foot Ulcers. INDIAN JOURNAL OF PHARMACEUTICAL SCIENCES. 2022;84:279-89.

164. Kandelaki G, Butsashvili M, Geleishvili M, Avaliani N, Macharashvili N, Topuridze M, et al. Nosocomial infections in Tbilisi, Georgia: a retrospective study of microbiological data from 4 major tertiary care hospitals. Infection control and hospital epidemiology. 2011;32(9):933-4.

165. Kansakar P, Dorji D, Chongtrakool P, Mingmongkolchai S, Mokmake B, Dubbs P. Local Dissemination of Multidrug-Resistant Acinetobacter baumannii Clones in a Thai Hospital. Microbial drug resistance (Larchmont, NY). 2010;17:109-19.

166. Mataracı Kara E, Yılmaz M, Özbek Çelik B. In vitro activities of ceftazidime/avibactam alone or in combination with antibiotics against multidrug-resistant Acinetobacter baumannii isolates. J Glob Antimicrob Resist. 2019;17:137-41.

167. Karagöz A, Baran I, Aksu N, Savas S, Durmaz R. Characterization and Determination of Antibiotic Resistance Profiles of a Single Clone Acinetobacter baumannii Strains Isolated from Blood Cultures. Mikrobiyoloji bülteni. 2014;48:566-76.

168. Karampatakis T, Geladari A, Politi L, Antachopoulos C, Iosifidis E, Tsiatsiou O, et al. Cluster-distinguishing genotypic and phenotypic diversity of carbapenem-resistant Gram-negative bacteria in solid-organ transplantation patients: a comparative study. J Med Microbiol. 2017;66(8):1158-69.

169. Hussain K, Salat MS, Ambreen G, Mughal A, Idrees S, Sohail M, et al. Intravenous vs intravenous plus aerosolized colistin for treatment of ventilator-associated pneumonia–a matched case–control study in neonates. Expert Opinion on Drug Safety. 2020;19(12):1641-9.

170. Novović K, Kuzmanović Nedeljković S, Poledica M, Nikolić G, Grujić B, Jovčić B, et al. Virulence potential of multidrug-resistant Acinetobacter baumannii isolates from COVID-19 patients on mechanical ventilation: The first report from Serbia. Frontiers in Microbiology. 2023;14:1094184.

171. Katchanov J, Asar L, Klupp EM, Both A, Rothe C, König C, et al. Carbapenem-resistant Gram-negative pathogens in a German university medical center: Prevalence, clinical implications and the role of novel β-lactam/β-lactamase inhibitor combinations. PLoS One. 2018;13(4):e0195757.

172. Thet KT, Lunha K, Srisrattakarn A, Lulitanond A, Tavichakorntrakool R, Kuwatjanakul W, et al. Colistin heteroresistance in carbapenem-resistant Acinetobacter baumannii clinical isolates from a Thai university hospital. World Journal of Microbiology and Biotechnology. 2020;36:1-7.

173. Slimene K, Ali AA, Mohamed EA, El Salabi A, Suliman FS, Elbadri AA, et al. Isolation of Carbapenem and Colistin Resistant Gram-Negative Bacteria Colonizing Immunocompromised SARS-CoV-2 Patients Admitted to Some Libyan Hospitals. Microbiology Spectrum. 2023:e02972-22.

174. Kirkgöz E, Zer Y. Clonal comparison of Acinetobacter strains isolated from intensive care patients and the intensive care unit environment. Turk J Med Sci. 2014;44(4):643-8.

175. Kishii K, Kikuchi K, Yoshida A, Okuzumi K, Uetera Y, Yasuhara H, et al. Antimicrobial susceptibility profile of Acinetobacter species isolated from blood cultures in two Japanese university hospitals. Microbiol Immunol. 2014;58(2):142-6.

176. Koca O. Antibiotic resistance profiles of endotracheal aspirates in intensive care unit patients. Journal of Clinical and Analytical Medicine. 2019;10:243-6.

177. Kołpa M, Wałaszek M, Gniadek A, Wolak Z, Dobroś W. Incidence, Microbiological Profile and Risk Factors of Healthcare-Associated Infections in Intensive Care Units: A 10 Year Observation in a Provincial Hospital in Southern Poland. Int J Environ Res Public Health. 2018;15(1).

178. Nafplioti K, Galani I, Angelidis E, Adamou P, Moraitou E, Giannopoulou P, et al. Dissemination of international clone II Acinetobacter baumannii strains coproducing OXA-23 carbapenemase and 16S rRNA methylase ArmA in Athens, Greece. Microbial Drug Resistance. 2020;26(1):9-13.

179. Mantzarlis K, Makris D, Zakynthinos E. Risk factors for the first episode of Acinetobacter baumannii resistant to colistin infection and outcome in critically ill patients. Journal of Medical Microbiology. 2020;69(1):35.

180. Kumar A, Randhawa VS, Nirupam N, Rai Y, Saili A. Risk factors for carbapenem-resistant Acinetobacter baumanii blood stream infections in a neonatal intensive care unit, Delhi, India. The Journal of Infection in Developing Countries. 2014;8(08):1049-54.

181. Kumar S, Jan RA, Fomda BA, Rasool R, Koul P, Shah S, et al. Healthcare-Associated Pneumonia and Hospital-Acquired Pneumonia: Bacterial Aetiology, Antibiotic Resistance and Treatment Outcomes: A Study From North India. Lung. 2018;196(4):469-79.

182. Peck KR, Kim MJ, Choi JY, Kim HS, Kang CI, Cho YK, et al. In vitro time-kill studies of antimicrobial agents against blood isolates of imipenem-resistant Acinetobacter baumannii, including colistin- or tigecycline-resistant isolates. J Med Microbiol. 2012;61(Pt 3):353-60.

183. Lai C-C, Chen Y-S, Lee N-Y, Tang H-J, Lee SS-J, Lin C-F, et al. Susceptibility rates of clinically important bacteria collected from intensive care units against colistin, carbapenems, and other comparative agents: results from Surveillance of Multicenter Antimicrobial Resistance in Taiwan (SMART). Infection and drug resistance. 2019;12:627-40.

184. Longjam LA, Tsering DC, Das D. A Microbiological Study of Acinetobacter calcoaceticus baumannii with Special Reference to Multidrug Resistance. Journal of Laboratory Physicians. 2021;14(02):169-74.

185. Perdigão Neto LV, Oliveira MS, Orsi TD, Prado G, Martins RCR, Leite GC, et al. Alternative drugs against multiresistant Gram-negative bacteria. J Glob Antimicrob Resist. 2020;23:33-7.

186. Al-Hassan L, Elbadawi H, Osman E, Ali S, Elhag K, Cantillon D, et al. Molecular epidemiology of carbapenem-resistant Acinetobacter baumannii from Khartoum State, Sudan. Frontiers in Microbiology. 2021;12:628736.

187. Duncan LR, Wang W, Sader HS. In vitro potency and spectrum of the novel polymyxin MRX-8 tested against clinical isolates of Gram-negative bacteria. Antimicrobial Agents and Chemotherapy. 2022;66(5):e00139-22.

188. Carrasco LDdM, Dabul ANG, Boralli CMdS, Righetto GM, Carvalho ISe, Dornelas JV, et al. Polymyxin resistance among XDR ST1 carbapenem-resistant Acinetobacter baumannii clone expanding in a teaching hospital. Frontiers in Microbiology. 2021;12:622704.

189. Hu L, Shi Y, Xu Q, Zhang L, He J, Jiang Y, et al. Capsule thickness, not biofilm formation, gives rise to mucoid Acinetobacter baumannii phenotypes that are more prevalent in long-term infections: a study of clinical isolates from a hospital in China. Infection and drug resistance. 2020:99-109.

190. Lin TC, Wu RX, Chiu CC, Yang YS, Lee Y, Lin JC, et al. The clinical and microbiological characteristics of infections in burn patients from the Formosa Fun Coast Dust Explosion. J Microbiol Immunol Infect. 2018;51(2):267-77.

191. Chen L, Yu K, Chen L, Zheng X, Huang N, Lin Y, et al. Synergistic activity and biofilm formation effect of colistin combined with PFK-158 against colistin-resistant gram-negative bacteria. Infection and Drug Resistance. 2021:2143-54.

192. Lowe M, Ehlers MM, Ismail F, Peirano G, Becker PJ, Pitout JDD, et al. Acinetobacter baumannii: Epidemiological and Beta-Lactamase Data From Two Tertiary Academic Hospitals in Tshwane, South Africa. Front Microbiol. 2018;9:1280.

193. Graña-Miraglia L, Evans BA, López-Jácome LE, Hernández-Durán M, Colín-Castro CA, Volkow-Fernández P, et al. Origin of OXA-23 variant OXA-239 from a recently emerged lineage of Acinetobacter baumannii international clone V. Msphere. 2020;5(1):10.1128/msphere. 00801-19.

194. Khan DM, Moosabba M, Rao IV. Changing antibiogram profile of Acinetobacter baumannii in diabetic and non-diabetic foot ulcer infections. Journal of Clinical and Diagnostic Research. 2018;12(5):DC12-DC6.

195. Khalil MA, Ahmed FA, Elkhateeb AF, Mahmoud EE, Ahmed MI, Ahmed RI, et al. Virulence characteristics of biofilm-forming acinetobacter baumannii in clinical isolates using a Galleria Mellonella Model. Microorganisms. 2021;9(11):2365.

196. Nikibakhsh M, Firoozeh F, Badmasti F, Kabir K, Zibaei M. Molecular study of metallo-β-lactamases and integrons in Acinetobacter baumannii isolates from burn patients. BMC Infectious Diseases. 2021;21:1-6.

197. Zafer MM, Hussein AF, Al-Agamy MH, Radwan HH, Hamed SM. Genomic characterization of extensively drug-resistant NDM-producing Acinetobacter baumannii clinical isolates with the emergence of novel blaADC-257. Frontiers in Microbiology. 2021;12:736982.

198. Urooj M, Ullah R, Ali S, Mohyuddin A, Mirza HM, Faryal R. Elucidation of molecular mechanism for colistin resistance among Gram-negative isolates from tertiary care hospitals. Journal of Infection and Chemotherapy. 2022;28(5):602-9.

199. Sengupta M, Banerjee S. Sulbactam and colistin susceptibility pattern among multidrug-resistant Acinetobacter isolates from respiratory samples. Cureus. 2022;14(2).

200. Jajoo M, Manchanda V, Chaurasia S, Sankar MJ, Gautam H, Agarwal R, et al. Alarming rates of antimicrobial resistance and fungal sepsis in outborn neonates in North India. PLoS One. 2018;13(6):e0180705.

201. Manohar P, Thamaraiselvan S, Ayyanar R, Bozdogan B, Wilson A, Tamhankar A, et al. The distribution of carbapenem- And colistin-resistance in Gram-negative bacteria from the Tamil Nadu region in India. Journal of Medical Microbiology. 2017;66.

202. Gysin M, Hon PY, Tan P, Sengduangphachanh A, Simmalavong M, Hinfonthong P, et al. Apramycin susceptibility of multidrug-resistant Gram-negative blood culture isolates in five countries in Southeast Asia. International Journal of Antimicrobial Agents. 2022;60(4):106659.

203. Jara MC, Frediani AV, Zehetmeyer FK, Bruhn FRP, Müller MR, Miller RG, et al. Multidrug-resistant hospital bacteria: epidemiological factors and susceptibility profile. Microbial drug resistance. 2021;27(3):433-40.

204. Markogiannakis H, Pachylaki N, Samara E, Kalderi M, Minettou M, Toutouza M, et al. Infections in a surgical intensive care unit of a university hospital in Greece. International Journal of Infectious Diseases. 2009;13(2):145-53.

205. Osman M, B Halimeh F, Rafei R, Mallat H, Tom JE, Raad EB, et al. Investigation of an XDR-Acinetobacter baumannii ST2 outbreak in an intensive care unit of a Lebanese tertiary care hospital. Future Microbiology. 2020;15(16):1535-42.

206. Seyyedi M, Shapouri R, Zeighami H, Shokoohizadeh L. Genetic diversity of colistin resistance Nosocomial Acinetobacter baumannii strains from Iran. Journal of Research in Medical Sciences: The Official Journal of Isfahan University of Medical Sciences. 2021;26.

207. Falagas ME, Skalidis T, Vardakas KZ, Legakis NJ, Group HCS. Activity of cefiderocol (S-649266) against carbapenem-resistant Gram-negative bacteria collected from inpatients in Greek hospitals. Journal of Antimicrobial Chemotherapy. 2017;72(6):1704-8.

208. Gavino Donadu M, Zanetti S, Nagy ÁL, Barrak IÁ, Gajdács M. Insights on carbapenem-resistant Acinetobacter baumannii: phenotypic characterization of relevant isolates. Acta Biologica Szegediensis. 2021;65(1):85-92.

209. Palmieri M, D’Andrea MM, Pelegrin AC, Perrot N, Mirande C, Blanc B, et al. Abundance of colistin-resistant, OXA-23-and ArmA-producing Acinetobacter baumannii belonging to international clone 2 in Greece. Frontiers in microbiology. 2020;11:668.

210. McCracken M, Mataseje LF, Loo V, Walkty A, Adam HJ, Hoban DJ, et al. Characterization of Acinetobacter baumannii and meropenem-resistant Pseudomonas aeruginosa in Canada: results of the CANWARD 2007-2009 study. Diagn Microbiol Infect Dis. 2011;69(3):335-41.

211. Medell M, Hart M, Duquesne A, Espinosa F, Valdés R. Nosocomial ventilator-associated pneumonia in Cuban intensive care units: bacterial species and antibiotic resistance. MEDICC Rev. 2013;15(2):26-9.

212. Heydarlou MM, Durmaz G, Ibrahi̇m BM. Evaluation of sulbactam and colistin/sulbactam efficacy against multiple resistant Acinetobacter baumannii blood isolates. Indian Journal of Medical Microbiology. 2022;40(4):567-71.

213. İpek MŞ, Özbek E. Bloodstream Infections in a Neonatal Intensive Care Unit Yenidoğan Yoğun Bakım Ünitesinde Kan Akımı Enfeksiyonları.

214. Mengeloğlu F, Çopur Çiçek A, Kocoglu E, Sandalli C, Budak E, Ozgumus O. Carriage of Class 1 and 2 Integrons in Acinetobacter baumannii and Pseudomonas aeruginosa Isolated from Clinical Specimens and a Novel Gene Cassette Array: bla(OXA-11)-cmlA7. Mikrobiyoloji bülteni. 2014;48:48-58.

215. Saleh Ahmed M, Abdulrahman ZFA, Taha ZMA. Risk Factors of Clonally Related, Multi, and Extensively Drug-Resistant Acinetobacter baumannii in Severely Ill COVID-19 Patients. Canadian Journal of Infectious Diseases and Medical Microbiology. 2023;2023.

216. Ababneh MA, Al Domi M, Rababa’h AM. Surveillance study of bloodstream infections, antimicrobial use, and resistance patterns among intensive care unit patients: A retrospective cross-sectional study. International Journal of Critical Illness and Injury Science. 2022;12(2):82.

217. Mezzatesta ML, Caio C, Gona F, Cormaci R, Salerno I, Zingali T, et al. Carbapenem and multidrug resistance in Gram-negative bacteria in a single centre in Italy: considerations on in vitro assay of active drugs. Int J Antimicrob Agents. 2014;44(2):112-6.

218. Huband MD, Mendes RE, Pfaller MA, Lindley JM, Strand GJ, Benn VJ, et al. In vitro activity of KBP-7072, a novel third-generation tetracycline, against 531 recent geographically diverse and molecularly characterized Acinetobacter baumannii species complex isolates. Antimicrobial Agents and Chemotherapy. 2020;64(5):10.1128/aac. 02375-19.

219. Lowe M, Singh-Moodley A, Ismail H, Thomas T, Chibabhai V, Nana T, et al. Molecular characterisation of Acinetobacter baumannii isolates from bloodstream infections in a tertiary-level hospital in South Africa. Frontiers in Microbiology. 2022:2938.

220. Gupta M, Naik AK, Singh SK. Bacteriological profile and antimicrobial resistance patterns of burn wound infections in a tertiary care hospital. Heliyon. 2019;5(12).

221. Al-Tamimi M, Albalawi H, Alkhawaldeh M, Alazzam A, Ramadan H, Altalalwah M, et al. Multidrug-resistant acinetobacter baumannii in Jordan. Microorganisms. 2022;10(5):849.

222. Namaei MH, Yousefi M, Askari P, Roshanravan B, Hashemi A, Rezaei Y. High prevalence of multidrug-resistant non-fermentative Gram-negative bacilli harboring blaIMP-1 and blaVIM-1 metallo-beta-lactamase genes in Birjand, south-east Iran. Iranian Journal of Microbiology. 2021;13(4):470.

223. Ghahraman MRK, Hosseini-Nave H, Azizi O, Shakibaie MR, Mollaie HR, Shakibaie S. Molecular characterization of lpxACD and pmrA/B two-component regulatory system in the colistin resistance Acinetobacter baumannii clinical isolates. Gene reports. 2020;21:100952.

224. Saleem M, Syed Khaja AS, Hossain A, Alenazi F, Said KB, Moursi SA, et al., editors. Molecular characterization and antibiogram of acinetobacter baumannii clinical isolates recovered from the patients with ventilator-associated pneumonia. Healthcare; 2022: MDPI.

225. Khurshid M, Rashid A, Husnain M, Rasool MH, Waqas U, Saeed M, et al. IN-VITRO ASSESSMENT OF THE THERAPEUTIC POTENTIAL OF POLYMYXINS AND TIGECYCLINE AGAINST MULTIDRUG-RESISTANT ACINETOBACTER ISOLATES FROM INFECTED WOUNDS. Journal of Ayub Medical College Abbottabad. 2020;32(4):459-64.

226. Khurshid M, Rasool MH, Ashfaq UA, Aslam B, Waseem M, Ali MA, et al. Acinetobacter baumannii sequence types harboring genes encoding aminoglycoside modifying enzymes and 16SrRNA methylase; a multicenter study from Pakistan. Infection and Drug Resistance. 2020:2855-62.

227. Al-Shamiri MM, Zhang S, Mi P, Liu Y, Xun M, Yang E, et al. Phenotypic and genotypic characteristics of Acinetobacter baumannii enrolled in the relationship among antibiotic resistance, biofilm formation and motility. Microbial Pathogenesis. 2021;155:104922.

228. Moon C, Kwak YG, Kim B-N, Kim B-N, Kim ES, Lee C-S. Implications of postneurosurgical meningitis caused by carbapenem-resistant Acinetobacter baumannii. Journal of Infection and Chemotherapy. 2013;19(5):916-9.

229. Ranaei MA, Shahraki-Zahedan S, Mohagheghi-Fard AH, Salimizand H, Ordoni R, Amini Y. Prevalence of the blaCTX-M and antibiotic resistance pattern among clinical isolates of Acinetobacter baumannii isolated from Zahedan, Southeast Iran. Gene Reports. 2020;19:100626.

230. Rao MR, Urs TA, Chitharagi VB, Shivappa S, Mahale RP, Gowda RS, et al. Rapid identification of carbapenemases by CarbAcineto NP test and the rate of beta-lactamases among Acinetobacter baumannii from a teaching hospital. Iranian Journal of Microbiology. 2022;14(2):174.

231. Shah MH, McAleese S, Kadam S, Parikh T, Vaidya U, Sanghavi S, et al. Emerging Antibiotic Resistance Patterns in a Neonatal Intensive Care Unit in Pune, India: A 2-Year Retrospective Study. Frontiers in pediatrics. 2022;10:864115.

232. Muthusamy D, Sudhishnaa S, Boppe A. Invitro Activities of Polymyxins and Rifampicin against Carbapenem Resistant Acinetobacter baumannii at a Tertiary Care Hospital from South India. Journal of clinical and diagnostic research : JCDR. 2016;10(9):DC15-DC8.

233. Choi M-J, Park YK, Peck KR, Ko KS. Mutant prevention concentrations of colistin used in combination with other antimicrobial agents against Acinetobacter baumannii, Klebsiella pneumoniae and Pseudomonas aeruginosa clinical isolates. International journal of antimicrobial agents. 2014;44(5):475-6.

234. Karah N, Khalid F, Wai SN, Uhlin BE, Ahmad I. Molecular epidemiology and antimicrobial resistance features of Acinetobacter baumannii clinical isolates from Pakistan. Annals of clinical microbiology and antimicrobials. 2020;19(1):1-13.

235. Benamrouche N, Lafer O, Benmahdi L, Benslimani A, Amhis W, Ammari H, et al. Phenotypic and genotypic characterization of multidrug-resistant Acinetobacter baumannii isolated in Algerian hospitals. The Journal of Infection in Developing Countries. 2020;14(12):1395-401.

236. Mohamed N, Ghazal A, Ahmed AAH, Zaki A. Prevalence and determinants of antimicrobial resistance of pathogens isolated from cancer patients in an intensive care unit in Alexandria, Egypt. Journal of the Egyptian Public Health Association. 2023;98(1):1-10.

237. Kohira N, Hackel MA, Oota M, Takemura M, Hu F, Mizuno H, et al. In vitro antibacterial activities of cefiderocol against Gram-negative clinical strains isolated from China in 2020. Journal of Global Antimicrobial Resistance. 2023;32:181-6.

238. Nazir A. Multidrug-resistant Acinetobacter septicemia in neonates: A study from a teaching hospital of Northern India. J Lab Physicians. 2019;11(1):23-8.

239. Muzahid NH, Hussain MH, Huët MAL, Dwiyanto J, Su TT, Reidpath D, et al. Molecular characterization and comparative genomic analysis of Acinetobacter baumannii isolated from the community and the hospital: an epidemiological study in Segamat, Malaysia. Microbial genomics. 2023;9(4).

240. Yousefi Nojookambari N, Sadredinamin M, Dehbanipour R, Ghalavand Z, Eslami G, Vaezjalali M, et al. Prevalence of β-lactamase-encoding genes and molecular typing of Acinetobacter baumannii isolates carrying carbapenemase OXA-24 in children. Annals of Clinical Microbiology and Antimicrobials. 2021;20(1):1-8.

241. Nemec A, Dijkshoorn L. Variations in colistin susceptibility among different species of the genus Acinetobacter. Journal of Antimicrobial Chemotherapy. 2010;65(2):367-9.

242. Saleh NM, Hesham MS, Amin MA, Samir Mohamed R. Acquisition of colistin resistance links cell membrane thickness alteration with a point mutation in the lpxD gene in Acinetobacter baumannii. Antibiotics. 2020;9(4):164.

243. Fam NS, Gamal D, Mohamed SH, Wasfy RM, Soliman MS, El-Kholy AA, et al. Molecular characterization of Carbapenem/Colistin-resistant Acinetobacter baumannii clinical isolates from Egypt by whole-genome sequencing. Infection and Drug Resistance. 2020:4487-93.

244. Fam NS, Mohamed SH, Gamal D, Wasfy RM, Soliman MS, El-Kholy AA. Reliability of phenotypic methods for detection of colistin resistance among carbapenem-resistant Acinetobacter baumannii clinical isolates from Egypt. Germs. 2020;10(4):303.

245. Nguyen P-S, Hai LTT, Nguyen M-K, Nguyen T-C, Tran H-D, Le Q-T, et al. Distribution and antibiotics resistance of emerging or reemerging pathogenic bacteria in 7A Military Hospital.

246. Nogbou N-D, Phofa DT, Nchabeleng M, Musyoki AM. Investigating multi-drug resistant Acinetobacter baumannii isolates at a tertiary hospital in Pretoria, South Africa. Indian Journal of Medical Microbiology. 2021;39(2):218-23.

247. Nordqvist H, Nilsson LE, Claesson C. Mutant prevention concentration of colistin alone and in combination with rifampicin for multidrug-resistant Acinetobacter baumannii. Eur J Clin Microbiol Infect Dis. 2016;35(11):1845-50.

248. Oleksiuk LM, Nguyen MH, Press EG, Updike CL, O'Hara JA, Doi Y, et al. In vitro responses of Acinetobacter baumannii to two- and three-drug combinations following exposure to colistin and doripenem. Antimicrob Agents Chemother. 2014;58(2):1195-9.

249. Perovic O, Duse A, Chibabhai V, Black M, Said M, Prentice E, et al. Acinetobacter baumannii complex, national laboratory-based surveillance in South Africa, 2017 to 2019. Plos one. 2022;17(8):e0271355.

250. Özbek B, Ötük G. In vitro activities of tigecycline alone and in combination with colistin sulfate or sulbactam against carbapenem-susceptible and-resistant Acinetobacter baumannii strains isolated from Intensive Care Units. International journal of antimicrobial agents. 2010;36(2):191-2.

251. Ozger HS, Cuhadar T, Yildiz SS, Demirbas Gulmez Z, Dizbay M, Guzel Tunccan O, et al. In vitro activity of eravacycline in combination with colistin against carbapenem-resistant A. baumannii isolates. J Antibiot (Tokyo). 2019;72(8):600-4.

252. Aydemir O, Aydemir Y, Şahin EÖ, Şahin F, Koroglu M, Erdem AF. Secondary bacterial infections in patients with coronavirus disease 2019-associated pneumonia. Revista da Associação Médica Brasileira. 2022;68:142-6.

253. Paluchowska P, Nowak P, Skalkowska M, Bjdak A. EVALUATION OF IN VITRO TIGECYCLINE ACTIVITY AGAINST MULTIDRUG-RESISTANT ACINETOBACTER BAUMANNII CLINICAL ISOLATES FROM POLAND. Acta Pol Pharm. 2017;74(3):793-800.

254. Di Carlo P, Serra N, Lo Sauro S, Carelli VM, Giarratana M, Signorello JC, et al. Epidemiology and pattern of resistance of gram-negative bacteria isolated from blood samples in hospitalized patients: a single center retrospective analysis from Southern Italy. Antibiotics. 2021;10(11):1402.

255. Park YK, Lee GH, Baek JY, Chung DR, Peck KR, Song JH, et al. A single clone of Acinetobacter baumannii, ST22, is responsible for high antimicrobial resistance rates of Acinetobacter spp. isolates that cause bacteremia and urinary tract infections in Korea. Microb Drug Resist. 2010;16(2):143-9.

256. Park YK, Peck KR, Cheong HS, Chung DR, Song JH, Ko KS. Extreme drug resistance in Acinetobacter baumannii infections in intensive care units, South Korea. Emerging infectious diseases. 2009;15(8):1325-7.

257. Chaturvedi P, Lamba M, Sharma D, Mamoria VP. Bloodstream infections and antibiotic sensitivity pattern in intensive care unit. Tropical Doctor. 2021;51(1):44-8.

258. Jia P, Zhu Y, Zhang H, Cheng B, Guo P, Xu Y, et al. In vitro activity of ceftaroline, ceftazidime-avibactam, and comparators against Gram-positive and-negative organisms in China: the 2018 results from the ATLAS program. BMC microbiology. 2022;22(1):1-13.

259. Ioannou P, Maraki S, Koumaki D, Manios GA, Koumaki V, Kassotakis D, et al. A Six-Year Retrospective Study of Microbiological Characteristics and Antimicrobial Resistance in Specimens from a Tertiary Hospital’s Surgical Ward. Antibiotics. 2023;12(3):490.

260. Phee LM, Kloprogge F, Morris R, Barrett J, Wareham DW, Standing JF. Pharmacokinetic-pharmacodynamic modelling to investigate in vitro synergy between colistin and fusidic acid against MDR Acinetobacter baumannii. Journal of Antimicrobial Chemotherapy. 2019;74(4):961-9.

261. Thelen P, Henriksen AS, Longshaw C, Yamano Y, Caldwell B, Hamprecht A. In vitro activity of cefiderocol against Gram-negative bacterial pathogens in Germany. J Glob Antimicrob Resist. 2022;28:12-7.

262. Khuntayaporn P, Kanathum P, Houngsaitong J, Montakantikul P, Thirapanmethee K, Chomnawang MT. Predominance of international clone 2 multidrug-resistant Acinetobacter baumannii clinical isolates in Thailand: a nationwide study. Annals of clinical microbiology and antimicrobials. 2021;20:1-11.

263. Potron A, Bour M, Triponney P, Muller J, Koebel C, R AB, et al. Sequential emergence of colistin and rifampicin resistance in an OXA-72- producing outbreak strain of Acinetobacter baumannii. Int J Antimicrob Agents. 2019;53(5):669-73.

264. Pournaras S, Dafopoulou K, Del Franco M, Zarkotou O, Dimitroulia E, Protonotariou E, et al. Predominance of international clone 2 OXA-23-producing-Acinetobacter baumannii clinical isolates in Greece, 2015: results of a nationwide study. Int J Antimicrob Agents. 2017;49(6):749-53.

265. Liu P-Y, Lee Y-L, Lu M-C, Shao P-L, Lu P-L, Chen Y-H, et al. National surveillance of antimicrobial susceptibility of bacteremic gram-negative bacteria with emphasis on community-acquired resistant isolates: report from the 2019 surveillance of multicenter antimicrobial resistance in Taiwan (SMART). Antimicrobial agents and chemotherapy. 2020;64(10):10.1128/aac. 01089-20.

266. Principe L, Capone A, Mazzarelli A, D'Arezzo S, Bordi E, Di Caro A, et al. In vitro activity of doripenem in combination with various antimicrobials against multidrug-resistant Acinetobacter baumannii: possible options for the treatment of complicated infection. Microb Drug Resist. 2013;19(5):407-14.

267. Mathur P, Malpiedi P, Walia K, Srikantiah P, Gupta S, Lohiya A, et al. Health-care-associated bloodstream and urinary tract infections in a network of hospitals in India: a multicentre, hospital-based, prospective surveillance study. The Lancet Global Health. 2022;10(9):e1317-e25.

268. Wang Q, Wang Z, Zhang F, Zhao C, Yang B, Sun Z, et al. Long-term continuous antimicrobial resistance surveillance among nosocomial gram-negative bacilli in China from 2010 to 2018 (CMSS). Infection and Drug Resistance. 2020:2617-29.

269. Karabay O, Ekşi F, Yıldırım MS. Investigation of antibiotic resistance profiles and carbapenemase resistance genes in Acinetobacter baumannii strains isolated from clinical samples. European Journal of Therapeutics. 2022;28(4):252-9.

270. Yang Q, Xu Y, Jia P, Zhu Y, Zhang J, Zhang G, et al. In vitro activity of sulbactam/durlobactam against clinical isolates of Acinetobacter baumannii collected in China. Journal of Antimicrobial Chemotherapy. 2020;75(7):1833-9.

271. Reddy BRC, Geetha R, Singh M, Rani RU, Nekkanti KN. Mcr-1 expression in progression of colistin resistance gram negative bacilli of clinical specimens derived from Intensive Care Units, wards and hospital setting of Deccan Eco Region of Southern India. Journal of Pharmaceutical Negative Results. 2022:295-305.

272. Bateman RM, Sharpe MD, Jagger JE, Ellis CG, Solé-Violán J, López-Rodríguez M, et al. 36th International Symposium on Intensive Care and Emergency Medicine : Brussels, Belgium. 15-18 March 2016. Crit Care. 2016;20(Suppl 2):94.

273. Reale M, Strazzulla A, Quirino A, Rizzo C, Marano V, Postorino MC, et al. Patterns of multi-drug resistant bacteria at first culture from patients admitted to a third level University hospital in Calabria from 2011 to 2014: implications for empirical therapy and infection control. Le Infezioni in Medicina. 2017;25(2):98-107.

274. El-Sokkary R, Uysal S, Erdem H, Kullar R, Pekok AU, Amer F, et al. Profiles of multidrug-resistant organisms among patients with bacteremia in intensive care units: An international ID-IRI survey. European Journal of Clinical Microbiology & Infectious Diseases. 2021;40(11):2323-34.

275. Han R, Ding L, Yang Y, Guo Y, Yin D, Wu S, et al. In vitro activity of KBP-7072 against 536 Acinetobacter baumannii complex isolates collected in China. Microbiology Spectrum. 2022;10(1):e01471-21.

276. Rezai MS, Rafiei A, Ahangarkani F, Bagheri-Nesami M, Nikkhah A, Shafahi K, et al. Emergence of Extensively Drug Resistant Acinetobacter baumannii-Encoding Integrons and Extended-Spectrum Beta-Lactamase Genes Isolated from Ventilator-Associated Pneumonia Patients. Jundishapur J Microbiol. 2017;10(7):e14377.

277. Hamzeh AR, Al Najjar M, Mahfoud M. Prevalence of antibiotic resistance among Acinetobacter baumannii isolates from Aleppo, Syria. American journal of infection control. 2012;40(8):776-7.

278. Rolain JM, Loucif L, Al-Maslamani M, Elmagboul E, Al-Ansari N, Taj-Aldeen S, et al. Emergence of multidrug-resistant Acinetobacter baumannii producing OXA-23 Carbapenemase in Qatar. New Microbes and New Infections. 2016;11:47-51.

279. Humphries RM, Janssen H, Hey-Hadavi JH, Hackel M, Sahm D. Multidrug-resistant Gram-negative bacilli recovered from respiratory and blood specimens from adults: the ATLAS surveillance program in European hospitals, 2018–2020. International Journal of Antimicrobial Agents. 2023;61(2):106724.

280. Rosales-Reyes R, Gayosso-Vázquez C, Fernández-Vázquez JL, Jarillo-Quijada MD, Rivera-Benítez C, Santos-Preciado JI, et al. Virulence profiles and innate immune responses against highly lethal, multidrug-resistant nosocomial isolates of Acinetobacter baumannii from a tertiary care hospital in Mexico. PLoS One. 2017;12(8):e0182899.

281. Khodashahi R, Naderi HR, Mohammadabadi M, Ataei R, Khodashahi M, Dadgarmoghaddam M, et al. Antimicrobial resistance patterns of bacterial and fungal isolates in COVID-19. Archives of Clinical Infectious Diseases. 2022;17(1).

282. Russo A, Bassetti M, Ceccarelli G, Carannante N, Losito AR, Bartoletti M, et al. Bloodstream infections caused by carbapenem-resistant Acinetobacter baumannii: Clinical features, therapy and outcome from a multicenter study. J Infect. 2019;79(2):130-8.

283. Mortazavi S, Farshadzadeh Z, Janabadi S, Musavi M, Shahi F, Moradi M, et al. Evaluating the frequency of carbapenem and aminoglycoside resistance genes among clinical isolates of Acinetobacter baumannii from Ahvaz, south-west Iran. New Microbes and New Infections. 2020;38:100779.

284. Bhagwat SS, Legakis NJ, Skalidis T, Loannidis A, Goumenopoulos C, Joshi PR, et al. In vitro activity of cefepime/zidebactam (WCK 5222) against recent Gram-negative isolates collected from high resistance settings of Greek hospitals. Diagnostic microbiology and infectious disease. 2021;100(3):115327.

285. Sader HS, Castanheira M, Mendes RE, Flamm RK. Frequency and antimicrobial susceptibility of Gram-negative bacteria isolated from patients with pneumonia hospitalized in ICUs of US medical centres (2015-17). J Antimicrob Chemother. 2018;73(11):3053-9.

286. Khoshnood S, Savari M, Abbasi Montazeri E, Farajzadeh Sheikh A. Survey on genetic diversity, biofilm formation, and detection of colistin resistance genes in clinical isolates of Acinetobacter baumannii. Infection and drug resistance. 2020:1547-58.

287. Ebrahimi S, Sisakhtpour B, Mirzaei A, Karbasizadeh V, Moghim S. E cacy of Isolated Bacteriophage Against Bio lm Embedded Colistin-Resistant Acinetobacter baumannii.

288. Meliani S, Toumi S, Djahoudi H, Deghdegh K, Amoura K, Djahoudi A. Synergistic combination of colistin with imipenem, amikacine or ciprofloxacin against Acinetobacter baumannii and Pseudomonas aeruginosa carbapenem-resistant isolated in Annaba hospital Algeria. Biocell. 2020;44(2):175.

289. Kaur S, Chaudhary J, Gupta V. A Clinico-Microbiological Study of Blood Stream Infections in a Tertiary Referral Hospital: Colistin Resistance & Challenges. Journal of Pure & Applied Microbiology. 2023;17(1).

290. Babaei S, Pourabdollah M, Aslanimehr M, Nikkhahi F, Mahmoodian S, Hasani Y, et al. Frequency of Multi-Drug Resistance and Molecular Characteristics of Resistance to Colistin in Acinetobacter baumannii Collected from Patients in Intensive Care Units with Ventilator-Associated Pneumonia. Tanaffos. 2021;20(4):345.

291. Pourajam S, Kalantari E, Talebzadeh H, Mellali H, Sami R, Soltaninejad F, et al. Secondary bacterial infection and clinical characteristics in patients with COVID-19 admitted to two intensive care units of an academic hospital in Iran during the first wave of the pandemic. Frontiers in cellular and infection microbiology. 2022;12:141.

292. Hamed SM, Hussein AF, Al-Agamy MH, Radwan HH, Zafer MM. Genetic Configuration of Genomic Resistance Islands in Acinetobacter baumannii Clinical Isolates From Egypt. Frontiers in Microbiology. 2022:2601.

293. Samonis G, Maraki S, Vouloumanou EK, Georgantzi GG, Kofteridis DP, Falagas ME. Antimicrobial susceptibility of non-fermenting Gram-negative isolates to isepamicin in a region with high antibiotic resistance. Eur J Clin Microbiol Infect Dis. 2012;31(11):3191-8.

294. Jakovac S, Goić-Barišić I, Pirija M, Kovačić A, Hrenović J, Petrović T, et al. molecular characterization and survival of carbapenem-resistant Acinetobacter baumannii isolated from hospitalized patients in Mostar, Bosnia and Herzegovina. Microbial Drug Resistance. 2021;27(3):383-90.

295. Yadav SK, Bhujel R, Mishra SK, Sharma S, Sherchand JB. Emergence of multidrug-resistant non-fermentative gram negative bacterial infection in hospitalized patients in a tertiary care center of Nepal. BMC research notes. 2020;13:1-6.

296. McLeod SM, Moussa SH, Hackel MA, Miller AA. In vitro activity of sulbactam-durlobactam against Acinetobacter baumannii-calcoaceticus complex isolates collected globally in 2016 and 2017. Antimicrobial Agents and Chemotherapy. 2020;64(4):10.1128/aac. 02534-19.

297. Seifert H, Stefanik D, Wisplinghoff H. Comparative in vitro activities of tigecycline and 11 other antimicrobial agents against 215 epidemiologically defined multidrug-resistant Acinetobacter baumannii isolates. J Antimicrob Chemother. 2006;58(5):1099-100.

298. Gorgun S, Guzel M, Gunal O, Kılıc SS. The efficiency of Colistin, Minocycline, Tigecycline, and Doxycycline against multidrug-resistant Acinetobacter strains. Ann Clin Anal Med. 2020.

299. Sevillano E, Fernandez E, Bustamante Z, Zabalaga S, Rosales I, Umaran A, et al. Emergence and clonal dissemination of carbapenem-hydrolysing OXA-58-producing Acinetobacter baumannii isolates in Bolivia. Journal of medical microbiology. 2012;61(1):80-4.

300. Abdi SN, Ghotaslou R, Asgharzadeh M, Mehramouz B, Hasani A, Baghi HB, et al. AdeB efflux pump gene knockdown by mRNA mediated peptide nucleic acid in multidrug resistance Acinetobacter baumannii. Microbial pathogenesis. 2020;139:103825.

301. Shah PG, Shah SR. Treatment and Outcome of Carbapenem-Resistant Gram-Negative Bacilli Blood-Stream Infections in a Tertiary Care Hospital. The Journal of the Association of Physicians of India. 2015;63(7):14-8.

302. Seleim SM, Mostafa MS, Ouda NH, Shash RY. The role of pmrCAB genes in colistin-resistant Acinetobacter baumannii. Scientific Reports. 2022;12(1):20951.

303. Khalid S, Ahmad N, Ali SM, Khan AU. Outbreak of efficiently transferred carbapenem-resistant bla NDM-producing gram-negative bacilli isolated from neonatal intensive care unit of an Indian hospital. Microbial Drug Resistance. 2020;26(3):284-9.

304. Mushtaq S, Sadouki Z, Vickers A, Livermore DM, Woodford N. In vitro activity of cefiderocol, a siderophore cephalosporin, against multidrug-resistant Gram-negative bacteria. Antimicrobial agents and chemotherapy. 2020;64(12):10.1128/aac. 01582-20.

305. Sheck EA, Edelstein MV, Sukhorukova MV, Ivanchik NV, Skleenova EY, Dekhnich AV, et al. Epidemiology and Genetic Diversity of Colistin Nonsusceptible Nosocomial Acinetobacter baumannii Strains from Russia for 2013-2014. Can J Infect Dis Med Microbiol. 2017;2017:1839190.

306. Jean S-S, Lee W-S, Yu K-W, Liao C-H, Hsu C-W, Chang F-Y, et al. Rates of susceptibility of carbapenems, ceftobiprole, and colistin against clinically important bacteria collected from intensive care units in 2007: results from the Surveillance of Multicenter Antimicrobial Resistance in Taiwan (SMART). Journal of Microbiology, Immunology and Infection. 2016;49(6):969-76.

307. Jean S-S, Lee Y-L, Liu P-Y, Lu M-C, Ko W-C, Hsueh P-R. Multicenter surveillance of antimicrobial susceptibilities and resistance mechanisms among Enterobacterales species and non-fermenting Gram-negative bacteria from different infection sources in Taiwan from 2016 to 2018. Journal of Microbiology, Immunology and Infection. 2022;55(3):463-73.

308. Chauhan S, Kaur N, Saini AK, Chauhan J, Kumar H. Assessment of colistin resistance in Gram negative bacteria from clinical samples in resource-limited settings. Asian pacific journal of tropical medicine. 2022;15(8):367-73.

309. Hsueh S-C, Lee Y-J, Huang Y-T, Liao C-H, Tsuji M, Hsueh P-R. In vitro activities of cefiderocol, ceftolozane/tazobactam, ceftazidime/avibactam and other comparative drugs against imipenem-resistant Pseudomonas aeruginosa and Acinetobacter baumannii, and Stenotrophomonas maltophilia, all associated with bloodstream infections in Taiwan. Journal of Antimicrobial Chemotherapy. 2019;74(2):380-6.

310. Ali S, Al-Haideri HH, Al Hishimi AM. Evaluating the Activity of Ultrasound on Biofilm Formation by Acinetobacter baumannii isolated from clinical Specimens. Baghdad Science Journal. 2022;19(6 (Suppl.)):1522-.

311. Singkham-In U, Chatsuwan T. In vitro activities of carbapenems in combination with amikacin, colistin, or fosfomycin against carbapenem-resistant Acinetobacter baumannii clinical isolates. Diagn Microbiol Infect Dis. 2018;91(2):169-74.

312. Ruekit S, Srijan A, Serichantalergs O, Margulieux KR, Mc Gann P, Mills EG, et al. Molecular characterization of multidrug-resistant ESKAPEE pathogens from clinical samples in Chonburi, Thailand (2017–2018). BMC infectious diseases. 2022;22(1):1-12.

313. Jun SH, Hwang HR, Kim N, Kwon KT, Kim YK, Lee JC. Clonal evolution and antimicrobial resistance of Acinetobacter baumannii isolates from Korean hospitals over the last decade. Infection, Genetics and Evolution. 2023;108:105404.

314. Ngoi ST, Chong CW, Ponnampalavanar SSLS, Tang SN, Idris N, Abdul Jabar K, et al. Genetic mechanisms and correlated risk factors of antimicrobial-resistant ESKAPEE pathogens isolated in a tertiary hospital in Malaysia. Antimicrobial Resistance & Infection Control. 2021;10:1-15.

315. Srinivas P, Hunt LN, Pouch SM, Thomas K, Goff DA, Pancholi P, et al. Detection of colistin heteroresistance in Acinetobacter baumannii from blood and respiratory isolates. Diagnostic Microbiology and Infectious Disease. 2018;91(2):194-8.

316. Strateva T, Sirakov I, Stoeva T, Stratev A, Dimov S, Savov E, et al. Carbapenem-resistant Acinetobacter baumannii: current status of the problem in four Bulgarian university hospitals (2014–2016). Journal of global antimicrobial resistance. 2019;16:266-73.

317. Khurana S, Singh P, Sharad N, Kiro VV, Rastogi N, Lathwal A, et al. Profile of co-infections & secondary infections in COVID-19 patients at a dedicated COVID-19 facility of a tertiary care Indian hospital: Implication on antimicrobial resistance. Indian journal of medical microbiology. 2021;39(2):147-53.

318. Chandran S, Manokaran Y, Vijayakumar S, Shankar BA, Bakthavatchalam YD, Dwarakanathan HT, et al. Enhanced bacterial killing with a combination of sulbactam/minocycline against dual carbapenemase-producing Acinetobacter baumannii. European Journal of Clinical Microbiology & Infectious Diseases. 2023;42(5):645-51.

319. Khanjani S, Sedigh Ebrahim-Saraie H, Shenagari M, Ashraf A, Mojtahedi A, Atrkar Roushan Z. In vitro activity of colistin against multidrug-resistant Acinetobacter baumannii isolates harboring blaOXA-23-like and blaOXA-24-like genes: A multicenter based study. Acta Microbiol Immunol Hung. 2020;67(3):182-6.

320. McKay SL, Vlachos N, Daniels JB, Albrecht VS, Stevens VA, Rasheed JK, et al. Molecular epidemiology of carbapenem-resistant Acinetobacter baumannii in the United States, 2013–2017. Microbial Drug Resistance. 2022;28(6):645-53.

321. Boorgula SY, Yelamanchili S, Kottapalli P, Naga MD. An Update on Secondary Bacterial and Fungal Infections and Their Antimicrobial Resistance Pattern (AMR) in COVID-19 Confirmed Patients. Journal of Laboratory Physicians. 2022;14(03):260-4.

322. Sharma S, Banerjee T, Yadav G, Kumar A. Susceptibility profile of blaOXA-23 and metallo-β-lactamases co-harbouring isolates of carbapenem resistant Acinetobacter baumannii (CRAB) against standard drugs and combinations. Frontiers in Cellular and Infection Microbiology. 2023;12:1068840.

323. Sharma S, Banerjee T, Yadav G, Palandurkar K. Mutations at novel sites in pmrA/B and lpxA/D genes and absence of reduced fitness in colistin-resistant Acinetobacter baumannii from a tertiary care hospital, India. Microbial Drug Resistance. 2021;27(5):628-36.

324. Kostyanev T, Xavier BB, García-Castillo M, Lammens C, Acosta JB-F, Rodríguez-Baño J, et al. Phenotypic and molecular characterizations of carbapenem-resistant Acinetobacter baumannii isolates collected within the EURECA study. International Journal of Antimicrobial Agents. 2021;57(6):106345.

325. Tada T, Miyoshi-Akiyama T, Shimada K, Shimojima M, Kirikae T. Dissemination of 16S rRNA methylase ArmA-producing acinetobacter baumannii and emergence of OXA-72 carbapenemase coproducers in Japan. Antimicrob Agents Chemother. 2014;58(5):2916-20.

326. Tada T, Miyoshi-Akiyama T, Shimada K, Nga TT, Thu le TA, Son NT, et al. Dissemination of clonal complex 2 Acinetobacter baumannii strains co-producing carbapenemases and 16S rRNA methylase ArmA in Vietnam. BMC Infect Dis. 2015;15:433.

327. Tada T, Miyoshi-Akiyama T, Kato Y, Ohmagari N, Takeshita N, Hung NV, et al. Emergence of 16S rRNA methylase-producing Acinetobacter baumannii and Pseudomonas aeruginosa isolates in hospitals in Vietnam. BMC Infect Dis. 2013;13:251.

328. Hafiz TA, Alghamdi SS, Mubaraki MA, Alghamdi SS, Alothaybi A, Aldawood E, et al. A two-year retrospective study of multidrug-resistant Acinetobacter baumannii respiratory infections in critically Ill patients: Clinical and microbiological findings. Journal of Infection and Public Health. 2023;16(3):313-9.

329. Tamayo M, Santiso R, Otero F, Bou G, Lepe JA, McConnell MJ, et al. Rapid determination of colistin resistance in clinical strains of Acinetobacter baumannii by use of the micromax assay. J Clin Microbiol. 2013;51(11):3675-82.

330. Tamayo-Legorreta E, Turrubiartes-Martínez E, Garza-Ramos U, Niño-Moreno P, Barrios H, Sánchez-Pérez A, et al. Outbreak Caused by blaOXA-72-Producing Acinetobacter baumannii ST417 Detected in Clinical and Environmental Isolates. Microb Drug Resist. 2016;22(2):129-33.

331. Paiboonvong T, Rodjun V, Houngsaitong J, Chomnawang M, Montakantikul P, Chulavatnatol S. Comparative in vitro activity of sitafloxacin against multidrug-resistant and carbapenem-resistant Acinetobacter baumannii clinical isolates in Thailand. Sci Asia. 2020;47:37-42.

332. Tada T, Uchida H, Hishinuma T, Watanabe S, Tohya M, Kuwahara-Arai K, et al. Molecular epidemiology of multidrug-resistant Acinetobacter baumannii isolates from hospitals in Myanmar. Journal of global antimicrobial resistance. 2020;22:122-5.

333. Tekin R, Dal T, Pirinccioglu H, Oygucu SE. A 4-year surveillance of device-associated nosocomial infections in a neonatal intensive care unit. Pediatr Neonatol. 2013;54(5):303-8.

334. Barcelos Valiatti T, Silva Carvalho T, Fernandes Santos F, Silva Nodari C, Cayô R, da Silva JTP, et al. Spread of multidrug-resistant Acinetobacter baumannii isolates belonging to IC1 and IC5 major clones in Rondônia state. Brazilian Journal of Microbiology. 2022;53(2):795-9.

335. Tripodi MF, Durante-Mangoni E, Fortunato R, Utili R, Zarrilli R. Comparative activities of colistin, rifampicin, imipenem and sulbactam/ampicillin alone or in combination against epidemic multidrug-resistant Acinetobacter baumannii isolates producing OXA-58 carbapenemases. Int J Antimicrob Agents. 2007;30(6):537-40.

336. Tsitsopoulos PP, Iosifidis E, Antachopoulos C, Anestis DM, Karantani E, Karyoti A, et al. Nosocomial bloodstream infections in neurosurgery: a 10-year analysis in a center with high antimicrobial drug-resistance prevalence. Acta neurochirurgica. 2016;158(9):1647-54.

337. Atik TK, Atik B, Kilinç O, Bektöre B, Duran H, Selek BM, et al. Epidemiological evaluation of an Acinetobacter baumannii outbreak observed at an intensive care unit. Saudi Medical Journal. 2018;39(8):767.

338. Ahsan U, Mushtaq F, Saleem S, Malik A, Sarfaraz H, Shahzad M, et al. Emergence of high colistin resistance in carbapenem resistant Acinetobacter baumannii in Pakistan and its potential management through immunomodulatory effect of an extract from Saussurea lappa. Frontiers in pharmacology. 2022;13:986802.

339. van Belkum A, Halimi D, Bonetti EJ, Renzi G, Cherkaoui A, Sauvonnet V, et al. Meropenem/colistin synergy testing for multidrug-resistant Acinetobacter baumannii strains by a two-dimensional gradient technique applicable in routine microbiology. J Antimicrob Chemother. 2015;70(1):167-72.

340. Goic-Barisic I, Kovacic A, Medic D, Jakovac S, Petrovic T, Tonkic M, et al. Endemicity of OXA-23 and OXA-72 in clinical isolates of Acinetobacter baumannii from three neighbouring countries in Southeast Europe. Journal of Applied Genetics. 2021;62:353-9.

341. Villalón P, Valdezate S, Medina M, Rubio V, Vindel A, Sáez-Nieto J-A. Clonal Diversity of Nosocomial Epidemic Acinetobacter baumannii Strains Isolated in Spain. Journal of clinical microbiology. 2010;49:875-82.

342. Trebosc V, Schellhorn B, Schill J, Lucchini V, Bühler J, Bourotte M, et al. In vitro activity of rifabutin against 293 contemporary carbapenem-resistant Acinetobacter baumannii clinical isolates and characterization of rifabutin mode of action and resistance mechanisms. Journal of Antimicrobial Chemotherapy. 2020;75(12):3552-62.

343. El-Kazzaz W, Metwally L, Yahia R, Al-Harbi N, El-Taher A, Hetta HF. Antibiogram, prevalence of OXA carbapenemase encoding genes, and RAPD-genotyping of multidrug-resistant Acinetobacter baumannii incriminated in hidden community-acquired infections. Antibiotics. 2020;9(9):603.

344. Wang TH, Leu YS, Wang NY, Liu CP, Yan TR. Prevalence of different carbapenemase genes among carbapenem-resistant Acinetobacter baumannii blood isolates in Taiwan. Antimicrob Resist Infect Control. 2018;7:123.

345. Shah MW, Yasir M, Farman M, Jiman-Fatani AA, Almasaudi SB, Alawi M, et al. Antimicrobial susceptibility and molecular characterization of clinical strains of Acinetobacter baumannii in Western Saudi Arabia. Microbial Drug Resistance. 2019;25(9):1297-305.

346. Saelim W, Changpradub D, Thunyaharn S, Juntanawiwat P, Nulsopapon P, Santimaleeworagun W. Colistin plus sulbactam or fosfomycin against carbapenem-resistant Acinetobacter baumannii: improved efficacy or decreased risk of nephrotoxicity? Infection & Chemotherapy. 2021;53(1):128.

347. Liang W, Yuan-Run Z, Min Y. Clinical Presentations and Outcomes of Post-Operative Central Nervous System Infection Caused by Multi-Drug–Resistant/Extensively Drug-Resistant Acinetobacter baumannii: A Retrospective Study. Surgical Infections. 2019;20(6):460-4.

348. Yau W, Owen RJ, Poudyal A, Bell JM, Turnidge JD, Heidi HY, et al. Colistin hetero-resistance in multidrug-resistant Acinetobacter baumannii clinical isolates from the Western Pacific region in the SENTRY antimicrobial surveillance programme. Journal of Infection. 2009;58(2):138-44.

349. Wisplinghoff H, Paulus T, Lugenheim M, Stefanik D, Higgins PG, Edmond MB, et al. Nosocomial bloodstream infections due to Acinetobacter baumannii, Acinetobacter pittii and Acinetobacter nosocomialis in the United States. J Infect. 2012;64(3):282-90.

350. Liu X, Zhao M, Chen Y, Bian X, Li Y, Shi J, et al. Synergistic killing by meropenem and colistin combination of carbapenem-resistant Acinetobacter baumannii isolates from Chinese patients in an in vitro pharmacokinetic/pharmacodynamic model. Int J Antimicrob Agents. 2016;48(5):559-63.

351. Dong X, Chen F, Zhang Y, Liu H, Liu Y, Ma L. In vitro activities of sitafloxacin tested alone and in combination with rifampin, colistin, sulbactam, and tigecycline against extensively drug-resistant Acinetobacter baumannii. International journal of clinical and experimental medicine. 2015;8(5):8135-40.

352. Dong X, Chen F, Zhang Y, Liu H, Liu Y, Ma L. In vitro activities of rifampin, colistin, sulbactam and tigecycline tested alone and in combination against extensively drug-resistant Acinetobacter baumannii. J Antibiot (Tokyo). 2014;67(9):677-80.

353. Wang X, Du Z, Huang W, Zhang X, Zhou Y. Outbreak of multidrug-resistant Acinetobacter baumannii ST208 producing OXA-23-like carbapenemase in a children's hospital in Shanghai, China. Microbial Drug Resistance. 2021;27(6):816-22.

354. Bian X, Liu X, Zhang X, Li X, Zhang J, Zheng H, et al. Epidemiological and genomic characteristics of Acinetobacter baumannii from different infection sites using comparative genomics. BMC genomics. 2021;22(1):1-13.

355. Bian X, Liu X, Chen Y, Chen D, Li J, Zhang J. Dose Optimization of Colistin Combinations against Carbapenem-Resistant Acinetobacter baumannii from Patients with Hospital-Acquired Pneumonia in China by Using an In Vitro Pharmacokinetic/Pharmacodynamic Model. Antimicrob Agents Chemother. 2019;63(4).

356. Kim YJ, Yoon JH, Kim SI, Hong KW, Kim JI, Choi JY, et al. High mortality associated with Acinetobacter species infection in liver transplant patients. Transplant Proc. 2011;43(6):2397-9.

357. Bai Y, Liu B, Wang T, Cai Y, Liang B, Wang R, et al. In Vitro activities of combinations of rifampin with other antimicrobials against multidrug-resistant Acinetobacter baumannii. Antimicrob Agents Chemother. 2015;59(3):1466-71.

358. Bahçe YG, Acer Ö, Özüdoğru O. Evaluation of bacterial agents isolated from endotracheal aspirate cultures of Covid-19 general intensive care patients and their antibiotic resistance profiles compared to pre-pandemic conditions. Microbial Pathogenesis. 2022;164:105409.

359. Anane YA, Apalata T, Vasaikar S, Okuthe GE, Songca S. Molecular detection of carbapenemase-encoding genes in multidrug-resistant Acinetobacter baumannii clinical isolates in South Africa. International journal of microbiology. 2020;2020.

360. Zhang Y, Zhao C, Wang Q, Wang X, Chen H, Li H, et al. Evaluation of the in vitro activity of new polymyxin B analogue SPR206 against clinical MDR, colistin-resistant and tigecycline-resistant Gram-negative bacilli. Journal of Antimicrobial Chemotherapy. 2020;75(9):2609-15.

361. Yazdansetad S, Najari E, Ghaemi EA, Javid N, Hashemi A, Ardebili A. Carbapenem-resistant Acinetobacter baumannii isolates carrying bla(OXA) genes with upstream ISAba1: First report of a novel OXA subclass from Iran. J Glob Antimicrob Resist. 2019;18:95-9.

362. Gu Y, Zhang W, Lei J, Zhang L, Hou X, Tao J, et al. Molecular epidemiology and carbapenem resistance characteristics of Acinetobacter baumannii causing bloodstream infection from 2009 to 2018 in northwest China. Frontiers in Microbiology. 2022;13:983963.

363. Hu Y-F, Hou CJ-Y, Kuo C-F, Wang N-Y, Wu AY-J, Leung C-H, et al. Emergence of carbapenem-resistant Acinetobacter baumannii ST787 in clinical isolates from blood in a tertiary teaching hospital in Northern Taiwan. Journal of microbiology, immunology and infection. 2017;50(5):640-5.

364. Lee Y-T, Sun J-R, Wang Y-C, Chiu C-H, Kuo S-C, Chen T-L, et al. Multicentre study of risk factors for mortality in patients with Acinetobacter bacteraemia receiving colistin treatment. International journal of antimicrobial agents. 2020;55(6):105956.

365. Kim Y-H. Conditional probability analysis of multidrug resistance in Gram-negative bacilli isolated from tertiary medical institutions in South Korea during 1999–2009. Journal of Microbiology. 2016;54:50-6.

366. Lee Y-L, Lu M-C, Shao P-L, Lu P-L, Chen Y-H, Cheng S-H, et al. Nationwide surveillance of antimicrobial resistance among clinically important Gram-negative bacteria, with an emphasis on carbapenems and colistin: Results from the Surveillance of Multicenter Antimicrobial Resistance in Taiwan (SMART) in 2018. International Journal of Antimicrobial Agents. 2019;54(3):318-28.

367. Duman Y, Ersoy Y, Tanriverdi ES, Otlu B, Toplu SA, Bağ HGG, et al. Oral colonization of Acinetobacter baumannii in intensive care units: Risk factors, incidence, molecular epidemiology, association with the occur of pneumonia and sepsis, and infection control measures. Iranian Journal of Basic Medical Sciences. 2022;25(2):239.

368. Meshkat Z, Salimizand H, Amini Y, Mansury D, Zomorodi AR, Avestan Z, et al. Detection of efflux pump genes in multiresistant Acinetobacter baumannii ST2 in Iran. Acta microbiologica et immunologica Hungarica. 2021;68(2):113-20.

369. Zapor MJ, Barber M, Summers A, Miller GH, Feeney LA, Eberly LE, et al. In vitro activity of the aminoglycoside antibiotic arbekacin against Acinetobacter baumannii-calcoaceticus isolated from war-wounded patients at Walter Reed Army Medical Center. Antimicrob Agents Chemother. 2010;54(7):3015-7.

370. Zarrilli R, Di Popolo A, Bagattini M, Giannouli M, Martino D, Barchitta M, et al. Clonal spread and patient risk factors for acquisition of extensively drug-resistant Acinetobacter baumannii in a neonatal intensive care unit in Italy. Journal of Hospital Infection. 2012;82(4):260-5.

371. Zhang F, Li Y, Lv Y, Zheng B, Xue F. Bacterial susceptibility in bloodstream infections: Results from China Antimicrobial Resistance Surveillance Trial (CARST) Program, 2015-2016. J Glob Antimicrob Resist. 2019;17:276-82.

372. Wei Z, Zhou S, Zhang Y, Zheng L, Zhao L, Cui Y, et al. Microbiological characteristics and risk factors on prognosis associated with Acinetobacter baumannii bacteremia in general hospital: A single-center retrospective study. Frontiers in Microbiology. 2022;13:1051364.

373. Zhu W, Wang Y, Cao W, Cao S, Zhang J. In vitro evaluation of antimicrobial combinations against imipenem-resistant Acinetobacter baumannii of different MICs. J Infect Public Health. 2018;11(6):856-60.

374. Liang W, Liu XF, Huang J, Zhu DM, Li J, Zhang J. Activities of colistin- and minocycline-based combinations against extensive drug resistant Acinetobacter baumannii isolates from intensive care unit patients. BMC Infect Dis. 2011;11:109.

375. Hu Q, Hu Z, Li J, Tian B, Xu H, Li J. Detection of OXA-type carbapenemases and integrons among carbapenem-resistant Acinetobactor baumannii in a teaching hospital in China. J Basic Microbiol. 2011;51(5):467-72.

376. Franolić-Kukina I, Bedenić B, Budimir A, Herljević Z, Vranes J, Higgins P. Clonal spread of carbapenem-resistant OXA-72-positive Acinetobacter baumannii in a Croatian university hospital. International journal of infectious diseases : IJID : official publication of the International Society for Infectious Diseases. 2011;15:e706-9.

377. Mammina C, Bonura C, Aleo A, Calà C, Caputo G, Cataldo MC, et al. Characterization of Acinetobacter baumannii from intensive care units and home care patients in Palermo, Italy. Clin Microbiol Infect. 2011;17(11):E12-5.

378. Cai Y, Li R, Liang B, Bai N, Liu Y, Wang R. In vitro antimicrobial activity and mutant prevention concentration of colistin against Acinetobacter baumannii. Antimicrobial agents and chemotherapy. 2010;54(9):3998-9.

379. Giannouli M, Cuccurullo S, Crivaro V, Di Popolo A, Bernardo M, Tomasone F, et al. Molecular epidemiology of multidrug-resistant Acinetobacter baumannii in a tertiary care hospital in Naples, Italy, shows the emergence of a novel epidemic clone. J Clin Microbiol. 2010;48(4):1223-30.

380. Routsi C, Pratikaki M, Platsouka E, Sotiropoulou C, Nanas S, Markaki V, et al. Carbapenem-resistant versus carbapenem-susceptible Acinetobacter baumannii bacteremia in a Greek intensive care unit: risk factors, clinical features and outcomes. Infection. 2010;38(3):173-80.

381. Jean SS, Hsueh PR, Lee WS, Chang HT, Chou MY, Chen IS, et al. Nationwide surveillance of antimicrobial resistance among non-fermentative Gram-negative bacteria in Intensive Care Units in Taiwan: SMART programme data 2005. Int J Antimicrob Agents. 2009;33(3):266-71.

382. McCracken M, DeCorby M, Fuller J, Loo V, Hoban DJ, Zhanel GG, et al. Identification of multidrug- and carbapenem-resistant Acinetobacter baumannii in Canada: results from CANWARD 2007. J Antimicrob Chemother. 2009;64(3):552-5.

383. Principe L, D'Arezzo S, Capone A, Petrosillo N, Visca P. In vitro activity of tigecycline in combination with various antimicrobials against multidrug resistant Acinetobacter baumannii. Ann Clin Microbiol Antimicrob. 2009;8:18.

384. Zarrilli R, Vitale D, Di Popolo A, Bagattini M, Daoud Z, Khan AU, et al. A plasmid-borne blaOXA-58 gene confers imipenem resistance to Acinetobacter baumannii isolates from a Lebanese hospital. Antimicrob Agents Chemother. 2008;52(11):4115-20.

385. Mezzatesta ML, Trovato G, Gona F, Nicolosi VM, Nicolosi D, Carattoli A, et al. In vitro activity of tigecycline and comparators against carbapenem-susceptible and resistant Acinetobacter baumannii clinical isolates in Italy. Annals of clinical microbiology and antimicrobials. 2008;7:4-.

386. Ko KS, Suh JY, Kwon KT, Jung SI, Park KH, Kang CI, et al. High rates of resistance to colistin and polymyxin B in subgroups of Acinetobacter baumannii isolates from Korea. J Antimicrob Chemother. 2007;60(5):1163-7.

387. Hawley JS, Murray CK, Griffith ME, McElmeel ML, Fulcher LC, Hospenthal DR, et al. Susceptibility of acinetobacter strains isolated from deployed U.S. military personnel. Antimicrob Agents Chemother. 2007;51(1):376-8.

388. Nawfal Dagher T, Al-Bayssari C, Chabou S, Antar N, Diene SM, Azar E, et al. Investigation of multidrug-resistant ST2 Acinetobacter baumannii isolated from Saint George hospital in Lebanon. BMC Microbiology. 2019;19(1):29.

389. Qamar MU, Walsh TR, Toleman MA, Tyrrell JM, Saleem S, Aboklaish A, et al. Dissemination of genetically diverse NDM-1, -5, -7 producing-Gram-negative pathogens isolated from pediatric patients in Pakistan. Future Microbiology. 2019;14(8):691-704.

390. Brasiliense D, Cayô R, Streling A, Nodari C, Barata R, Lemos P, et al. Diversity of metallo-β-lactamase-encoding genes found in distinct species of Acinetobacter isolated from the Brazilian Amazon Region. Memórias do Instituto Oswaldo Cruz. 2019;114.

391. Ramadan RA, Gebriel MG, Kadry HM, Mosallem A. Carbapenem-resistant Acinetobacter baumannii and Pseudomonas aeruginosa: characterization of carbapenemase genes and E-test evaluation of colistin-based combinations. Infect Drug Resist. 2018;11:1261-9.

392. Kaskatepe B, Yildiz SS, Mumcuoglu I, Ozet G, Altuntas EG. RS sample: Can be guide for empirical treatment of haematological malignancy patients? Microb Pathog. 2018;125:164-7.

393. Khan FY, Abu-Khattab M, Almaslamani EA, Hassan AA, Mohamed SF, Elbuzdi AA, et al. Acute bacterial meningitis in Qatar: a hospital-based study from 2009 to 2013. BioMed research international. 2017;2017.

394. Shah PG, Shah SR, Kamat S, Kamat DV. Colistin-carbapenem combination therapy against carbapenem resistant gram negative bacilli infections: Clinical and an in vitro synergy study. Int J Pharm Pharmaceutical Sci. 2014;6(10):497-500.

395. Principe L, Piazza A, Giani T, Bracco S, Caltagirone MS, Arena F, et al. Epidemic diffusion of OXA-23-producing Acinetobacter baumannii isolates in Italy: results of the first cross-sectional countrywide survey. J Clin Microbiol. 2014;52(8):3004-10.

396. Sieniawski K, Kaczka K, Rucińska M, Gagis L, Pomorski L. Acinetobacter baumannii nosocomial infections. Pol Przegl Chir. 2013;85(9):483-90.

397. Bahador A, Taheri M, Pourakbari B, Hashemizadeh Z, Rostami H, Mansoori N, et al. Emergence of rifampicin, tigecycline, and colistin-resistant Acinetobacter baumannii in Iran; spreading of MDR strains of novel International Clone variants. Microb Drug Resist. 2013;19(5):397-406.

398. Alsultan AA, Aboulmagd E, Evans BA, Amyes SG. Clonal diversity of Acinetobacter baumannii from diabetic patients in Saudi Arabian hospitals. Journal of medical microbiology. 2014;63(11):1460-6.

399. Kumar A, VinodKumar C. Detection of carbapenem resistance encoding genes among gram negative bacteria from urinary tract infection in patients with type 2 diabetes mellitus. Journal of Pure and Applied Microbiology. 2017;11(2):1061-6.

400. Al-Agamy MH, Khalaf NG, Tawfick MM, Shibl AM, Kholy AE. Molecular characterization of carbapenem-insensitive Acinetobacter baumannii in Egypt. International Journal of Infectious Diseases. 2014;22:49-54.

401. Alsultan AA, Evans BA, Elsayed EA, Al-Thawadi SI, Al-Taher AY, Amyes SGB, et al. High frequency of carbapenem-resistant Acinetobacter baumannii in patients with diabetes mellitus in Saudi Arabia. J Med Microbiol. 2013;62(Pt 6):885-8.

402. Al-Sultan AA, Evans BA, Aboulmagd E, Al-Qahtani AA, Bohol MF, Al-Ahdal MN, et al. Dissemination of multiple carbapenem-resistant clones of Acinetobacter baumannii in the Eastern District of Saudi Arabia. Front Microbiol. 2015;6:634.

403. Ying C, Li Y, Wang Y, Zheng B, Yang C. Investigation of the molecular epidemiology of Acinetobacter baumannii isolated from patients and environmental contamination. The Journal of antibiotics. 2015;68(9):562-7.

404. Ogbolu DO, Alli OAT, Oluremi AS, Ogunjimi YT, Ojebode DI, Dada V, et al. Contribution of NDM and OXA-type carbapenemases to carbapenem resistance in clinical Acinetobacter baumannii from Nigeria. Infectious Diseases. 2020;52(9):644-50.

405. Dizbay M, Tozlu DK, Cirak MY, Isik Y, Ozdemir K, Arman D. In vitro synergistic activity of tigecycline and colistin against XDR-Acinetobacter baumannii. The Journal of Antibiotics. 2010;63(2):51-3.

406. Mazraeh FN, Hasani A, Sadeghi J, Kafil HS, Barhaghi MHS, Sefidan FY, et al. High frequency of blaPER-1 gene in clinical strains of Acinetobacter baumannii and its association with quorum sensing and virulence factors. Gene Reports. 2021;24:101232.

407. Al-Hashem G, Rotimi VO, Albert MJ. Antimicrobial resistance of serial isolates of Acinetobacter baumannii colonizing the rectum of adult intensive care unit patients in a teaching hospital in Kuwait. Microbial Drug Resistance. 2021;27(1):64-72.

408. Gurung M, Rho JS, Lee YC, Kim HS, Moon SY, Yu BH, et al. Emergence and spread of carbapenem-resistant Acinetobacter baumannii sequence type 191 in a Korean hospital. Infect Genet Evol. 2013;19:219-22.

409. Rhim H, Trad RB, Haddad O, Kadri Y, Mastouri M. Comparative study of multidrug-resistant bacterial infections in hospitals and community settings in the region of Monastir–Tunisia. La Tunisie Medicale. 2022;100(5):390.

410. Wang H, Guo P, Sun H, Wang H, Yang Q, Chen M, et al. Molecular epidemiology of clinical isolates of carbapenem-resistant Acinetobacter spp. from Chinese hospitals. Antimicrob Agents Chemother. 2007;51(11):4022-8.

411. Lee H, Roh KH, Hong SG, Shin HB, Jeong SH, Song W, et al. In Vitro Synergistic Effects of Antimicrobial Combinations on Extensively Drug-Resistant Pseudomonas aeruginosa and Acinetobacter baumannii Isolates. Ann Lab Med. 2016;36(2):138-44.

412. Jeon H, Kim S, Kim MH, Kim SY, Nam D, Park SC, et al. Molecular epidemiology of carbapenem-resistant Acinetobacter baumannii isolates from a Korean hospital that carry bla(OXA-23). Infect Genet Evol. 2018;58:232-6.

413. Li J, Fu Y, Zhang J, Zhao Y, Fan X, Yu L, et al. The efficacy of colistin monotherapy versus combination therapy with other antimicrobials against carbapenem-resistant Acinetobacter baumannii ST2 isolates. Journal of Chemotherapy. 2020;32(7):359-67.

414. Lee JH, Choi CH, Kang HY, Lee JY, Kim J, Lee YC, et al. Differences in phenotypic and genotypic traits against antimicrobial agents between Acinetobacter baumannii and Acinetobacter genomic species 13TU. Journal of Antimicrobial Chemotherapy. 2007;59(4):633-9.

415. AlQumaizi KI, Kumar S, Anwer R, Mustafa S. Differential gene expression of efflux pumps and porins in clinical isolates of MDR Acinetobacter baumannii. Life. 2022;12(3):419.

416. Lee YT, Kuo SC, Yang SP, Lin YT, Chiang DH, Tseng FC, et al. Bacteremic nosocomial pneumonia caused by Acinetobacter baumannii and Acinetobacter nosocomialis: a single or two distinct clinical entities? Clin Microbiol Infect. 2013;19(7):640-5.

417. Gao L, Lyu Y, Li Y. Trends in Drug Resistance of Acinetobacter baumannii over a 10-year Period: Nationwide Data from the China Surveillance of Antimicrobial Resistance Program. Chin Med J (Engl). 2017;130(6):659-64.

418. Lee M-H, Chen T-L, Lee Y-T, Huang L, Kuo S-C, Yu K-W, et al. Dissemination of multidrug-resistant Acinetobacter baumannii carrying BlaOxA-23 from hospitals in central Taiwan. Journal of Microbiology, Immunology and Infection. 2013;46(6):419-24.

419. Nageeb W, Kamel M, Elazab S, Metwally L. Phenotypic characterization of Acinetobacter baumannii isolates from intensive care units at a tertiary-care hospital in Egypt. Eastern Mediterranean health journal = La revue de santé de la Méditerranée orientale = al-Majallah al-ṣiḥḥīyah li-sharq al-mutawassiṭ. 2018;20:203-11.

420. Karmostaji A, Javadpour S, Davoodian P, Moradi N. In vitro activity of tigecycline and colistin against clinical isolates of Acinetobacter baumannii in hospitals in Tehran and Bandar-Abbas, Iran. Electronic physician. 2014;6(3):919.

421. Namiganda V, Mina Y, Meklat A, Touati D, Bouras N, Barakate M, et al. Antibiotic Resistance Pattern of Acinetobacter baumannii Strains Isolated from Different Clinical Specimens and Their Sensibility Against Bioactive Molecules Produced by Actinobacteria. Arabian Journal for Science and Engineering. 2019;44(7):6267-75.

422. Ahmed N, Khan M, Saleem W, Karobari MI, Mohamed RN, Heboyan A, et al. Evaluation of bi-lateral co-infections and antibiotic resistance rates among COVID-19 patients. Antibiotics. 2022;11(2):276.

423. Rodríguez CH, Nastro M, Vay C, Famiglietti A. In vitro activity of minocycline alone or in combination in multidrug-resistant Acinetobacter baumannii isolates. J Med Microbiol. 2015;64(10):1196-200.

424. Chen T, Fu Y, Hua X, Xu Q, Lan P, Jiang Y, et al. Acinetobacter baumannii strains isolated from cerebrospinal fluid (CSF) and bloodstream analysed by cgMLST: the dominance of clonal complex CC92 in CSF infections. International Journal of Antimicrobial Agents. 2021;58(4):106404.

425. Vasconcellos FM, Casas MRT, Tavares LCB, Garcia DO, Camargo CH. In vitro activity of antimicrobial agents against multidrug- and extensively drug-resistant Acinetobacter baumannii. J Med Microbiol. 2017;66(1):98-102.

426. Wei WJ, Yang HF. Synergy against extensively drug-resistant Acinetobacter baumannii in vitro by two old antibiotics: colistin and chloramphenicol. Int J Antimicrob Agents. 2017;49(3):321-6.

427. Hsieh WS, Wang NY, Feng JA, Weng LC, Wu HH. Types and prevalence of carbapenem-resistant Acinetobacter calcoaceticus-Acinetobacter baumannii complex in Northern Taiwan. Antimicrob Agents Chemother. 2014;58(1):201-4.

428. Ni W, Cui J, Liang B, Cai Y, Bai N, Cai X, et al. In vitro effects of tigecycline in combination with colistin (polymyxin E) and sulbactam against multidrug-resistant Acinetobacter baumannii. The Journal of antibiotics. 2013;66(12):705-8.

429. Ku WW, Kung CH, Lee CH, Tseng CP, Wu PF, Kuo SC, et al. Evolution of carbapenem resistance in Acinetobacter baumannii: an 18-year longitudinal study from a medical center in northern Taiwan. J Microbiol Immunol Infect. 2015;48(1):57-64.

430. Yang YS, Lee YT, Tsai WC, Kuo SC, Sun JR, Yang CH, et al. Comparison between bacteremia caused by carbapenem resistant Acinetobacter baumannii and Acinetobacter nosocomialis. BMC Infect Dis. 2013;13:311.

431. Zhao Y, Zhu Y, Zhang H, Zhang L, Li J, Ye Y. Molecular tracking of carbapenem-resistant Acinetobacter baumannii clinical isolates: a multicentre study over a 4-year period across eastern China. Journal of Medical Microbiology. 2023;72(2):001655.

432. Li Y, Guo Q, Wang P, Zhu D, Ye X, Wu S, et al. Clonal dissemination of extensively drug-resistant Acinetobacter baumannii producing an OXA-23 β-lactamase at a teaching hospital in Shanghai, China. Journal of Microbiology, Immunology and Infection. 2015;48(1):101-8.

433. Lee Y-T, Huang L-Y, Chiang D-H, Chen C-P, Chen T-L, Wang F-D, et al. Differences in phenotypic and genotypic characteristics among imipenem-non-susceptible Acinetobacter isolates belonging to different genomic species in Taiwan. International Journal of Antimicrobial Agents. 2009;34(6):580-4.

434. Chuang YC, Sheng WH, Lauderdale TL, Li SY, Wang JT, Chen YC, et al. Molecular epidemiology, antimicrobial susceptibility and carbapenemase resistance determinants among Acinetobacter baumannii clinical isolates in Taiwan. J Microbiol Immunol Infect. 2014;47(4):324-32.

435. Huang Y-S, Wang J-T, Sheng W-H, Chuang Y-C, Chang S-C. Comparative in vitro activity of sitafloxacin against bacteremic isolates of carbapenem resistant Acinetobacter baumannii complex. Journal of Microbiology, Immunology and Infection. 2015;48(5):545-51.

436. Dargahi Z, Hamad AA, Sheikh AF, Ahmad Khosravi N, Samei Fard S, Motahar M, et al. The biofilm formation and antibiotic resistance of bacterial profile from endotracheal tube of patients admitted to intensive care unit in southwest of Iran. PLoS One. 2022;17(11):e0277329.

437. Moehario LH, Esterita T, Shirleen V, Robertus T, Angelina Y. Association of Acinetobacter Baumannii with invasive procedures in hospitalized patients in Jakarta. The Journal of Infection in Developing Countries. 2020;14(12):1455-60.

438. ABHISHEKMEHTA MKD. CHARACTERIZATION AND ANTIMICROBIAL SUSCEPTIBILITY PROFILE OF NON LACTOSE

FERMENTING GRAM-NEGATIVE BACTERIAL ISOLATES IN A TERTIARY CARE TEACHING

HOSPITAL OF CENTRAL INDIA. Asian J Pharm Clin Res,. 2021;14(10):41-7.

439. Zahlane K, Ouafi AT, Barakate M. The clinical and epidemiological risk factors of infections due to multi-drug resistant bacteria in an adult intensive care unit of University Hospital Center in Marrakesh-Morocco. Journal of Infection and Public Health. 2020;13(4):637-43.

440. Ahdi Khosroshahi S, Farajnia S, Azhari F, Hosseini MK, Khanipour F, Farajnia H, et al. Antimicrobial Susceptibility Pattern and Prevalence of Extended-Spectrum β-Lactamase Genotypes among Clinical Isolates of Acinetobacter baumanii in Tabriz, North-West of Iran. Jundishapur J Microbiol. 2017;10(6):e13368.

441. Mahmoud AB, Mohamed SA, Eldahshan MM. FimH and CsgA Adhesion genes Among Acinetobacter spp. Isolates and their Relation to Biofilm formation and Antimicrobial Resistance Pattern. Egyptian Journal of Medical Microbiology. 2020;29(1):77-86.

442. Akrami F, Shahandashti EF, Yahyapour Y, Sadeghi M, Khafri S, Pournajaf A, et al. Integron types, gene cassettes and antimicrobial resistance profile of Acinetobacter baumannii isolated from BAL samples in Babol, north of Iran. Microb Pathog. 2017;109:35-8.

443. Al Atrouni A, Hamze M, Jisr T, Lemarié C, Eveillard M, Joly-Guillou ML, et al. Wide spread of OXA-23-producing carbapenem-resistant Acinetobacter baumannii belonging to clonal complex II in different hospitals in Lebanon. Int J Infect Dis. 2016;52:29-36.

444. Matalka Aa, Al-Husban N, Alkuran O, Almuhaisen L, Basha A, Eid M, et al. Spectrum of uropathogens and their susceptibility to antimicrobials in pregnant women: A retrospective analysis of 5-year hospital data. Journal of International Medical Research. 2021;49(5):03000605211006540.

445. Alfadli M, El-sehsah E, Ramadan M. Risk factors and distribution of MDROs among patients with healthcare associated burn wound infection. Germs. 2018;8:199-206.

446. Alharbi A, Alshami I. In vitro Effects of Tigecycline in Combination with Other Antimicrobials against Multidrug-Resistant Acinetobacter baumannii Isolates. Journal of Pure and Applied Microbiology. 2015;9:497-502.

447. Aliakbarzade K, Farajnia S, Karimi Nik A, Zarei F, Tanomand A. Prevalence of Aminoglycoside Resistance Genes in Acinetobacter baumannii Isolates. Jundishapur journal of microbiology. 2014;7(10):e11924-e.

448. Almomani B, McCullough A, Gharaibeh R, Samrah S, Mahasneh F. Incidence and predictors of 14-day mortality in multidrug-resistant Acinetobacter baumannii in ventilator-associated pneumonia. Journal of infection in developing countries. 2015;9:1323-30.

449. Alp E, Eren E, Elay G, Cevahir F, Esmaoğlu A, Rello J. Efficacy of loading dose of colistin in Acinetobacter baumannii ventilator-associated pneumonia. Infez Med. 2017;25(4):311-9.

450. Fatima A, Fasih F, Naseem S, Sajjad M, Gohar H, Bukhari U. Bacteriologic Profile and Antibiotic Susceptibility in Patients with UTIs in Tertiary Care Hospital. Journal of Liaquat University of Medical & Health Sciences. 2022;21(04):252-7.

451. Emami A, Pirbonyeh N, Keshavarzi A, Bazargani A, Hassanpour S, Javanmardi F. Evaluating the saliva of burn ICU patients for resistant infections harbor metallo-β-lactamase genes. Journal of Burn Care & Research. 2020;41(3):647-51.

452. Adaimé A, Hajj A, Hallit S, Sarkis DK. Lebanese observatory of pathogenic agents (LOPA-study) a 2 year-surveillance prospective study. LMJ-Lebanese Medical Journal. 2020;68(3):126-33.

453. Andriamanantena TS, Ratsima E, Rakotonirina HC, Randrianirina F, Ramparany L, Carod J-F, et al. Dissemination of multidrug resistant Acinetobacter baumannii in various hospitals of Antananarivo Madagascar. Annals of clinical microbiology and antimicrobials. 2010;9:17-.

454. Chaurasia A, Shinde A, Baveja S. Bacteriological profile and antimicrobial susceptibility pattern of cerebrospinal fluid shunt infections in infants and children. Journal of Pediatric Neurosciences. 2021;16(2):143.

455. Ansari H, Doosti A, Kargar M, Bijanzadeh M, Jafarinya M. Antimicrobial resistant determination and prokaryotic expression of smpA gene of Acinetobacter baumannii isolated from admitted patients. Jundishapur Journal of Microbiology. 2017;10(11).

456. Armin S, Fallah F, Azimi L, Kafil H, Karimi A, Ghazvini K. Warning: spread of NDM-1 in two border towns of Iran. Cellular and molecular biology (Noisy-le-Grand, France). 2018;64:125-9.

457. NAZLI ZEKA A, ARDA B, SİPAHİ O, UYAR M. Factors Related to Mortality in Carbapenem Resistant Acinetobacter baumannii Infections in Intensive Care Units: A Prospective Observational Study. FLORA INFEKSIYON HASTALIKLARI VE KLINIK MIKROBIYOLOJI DERGISI. 2020;25(3).

458. Shah AA, Ali Y, Maqbool A, Abbasi SA. Phenotypic detection of extended-spectrum beta-lactamase in multidrug-resistant acinetobacter baumannii isolated in Fauji Foundation Hospital Rawalpindi. JPMA. 2021;71(1144).

459. Ejaz A, Khawaja A, Arshad F, Tauseef A, Ullah R, Ahmad I. Etiological profile and antimicrobial patterns in blood culture specimens in a tertiary care setting. Cureus. 2020;12(10).

460. Khodier AA, Saafan A, Bakeer W, Khairalla AS. Molecular Characterization of Multiple Antibiotic-Resistant Acinetobacter baumannii Isolated from Egyptian Patients. J Pure Appl Microbiol. 2020;14:2399-405.

461. Azizi O, Shakibaie MR, Modarresi F, Shahcheraghi F. Molecular Detection of Class-D OXA Carbapenemase Genes in Biofilm and Non-Biofilm Forming Clinical Isolates of Acinetobacter baumannii. Jundishapur journal of microbiology. 2015;8(1):e21042-e.

462. Babaei AH, Pouladfar G, Pourabbas B, Jafarpour Z, Ektesabi S, Abbasi P. Seven-Year Trend of Antimicrobial Resistance of Acinetobacter and Pseudomonas spp. Causing Bloodstream Infections: A Retrospective Study from Shiraz, Southern Iran. Jundishapur J Microbiol. 2019;12(4):e85819.

463. Bagheri Josheghani S, Moniri R, Firoozeh F, Sehat M, Dasteh Goli Y. Susceptibility Pattern and Distribution of Oxacillinases and bla PER-1 Genes among Multidrug Resistant Acinetobacter baumannii in a Teaching Hospital in Iran. J Pathog. 2015;2015:957259.

464. Bouharkat B, Tir Touil A, Mullié C, Chelli N, Meddah B. Bacterial ecology and antibiotic resistance mechanisms of isolated resistant strains from diabetic foot infections in the north west of Algeria. Journal of Diabetes & Metabolic Disorders. 2020;19:1261-71.

465. Balkhair A, Al-Muharrmi Z, Al'Adawi B, Al Busaidi I, Taher HB, Al-Siyabi T, et al. Prevalence and 30-day all-cause mortality of carbapenem-and colistin-resistant bacteraemia caused by Acinetobacter baumannii, Pseudomonas aeruginosa, and Klebsiella pneumoniae: Description of a decade-long trend. Int J Infect Dis. 2019;85:10-5.

466. Ballouz T, Aridi J, Afif C, Irani J, Lakis C, Nasreddine R, et al. Risk Factors, Clinical Presentation, and Outcome of Acinetobacter baumannii Bacteremia. Front Cell Infect Microbiol. 2017;7:156.

467. Hashemi B, Afkhami H, Khaledi M, Kiani M, Bialvaei AZ, Fathi J, et al. Frequency of Metalo beta Lactamase genes, bla IMP1, INT 1 in Acinetobacter baumanii isolated from burn patients North of Iran. Gene Reports. 2020;21:100800.

468. Balázs B, Tóth Z, Nagy F, Kovács R, Tóth H, Nagy JB, et al. The Role of Uniform Meropenem Usage in Acinetobacter baumannii Clone Replacement. Antibiotics. 2021;10(2):127.

469. Brahmi N, Beji O, Abidi N, Kouraichi N, Blel Y, Ghord HE, et al. Epidemiology and risk factors for colonization and infection by Acinetobacter baumannii in an ICU in Tunisia, where this pathogen is endemic. Journal of Infection and Chemotherapy. 2007;13(6):400-4.

470. Chaudhary BR, Malla KK, Poudel S, Jha BK. Study of antibiotic susceptibility among bacterial isolates in neonatal intensive care unit of a tertiary care hospital: a descriptive cross-sectional study. JNMA: Journal of the Nepal Medical Association. 2020;58(231):893.

471. Cetin ES, Durmaz R, Tetik T, Otlu B, Kaya S, Calişkan A. Epidemiologic characterization of nosocomial Acinetobacter baumannii infections in a Turkish university hospital by pulsed-field gel electrophoresis. Am J Infect Control. 2009;37(1):56-64.

472. Behera IC, Swain SK, Sahu MC. COLONIZATION AND ANTIBIOTIC RESISTANCE DYNAMICS OF PATIENTS AT INTENSIVE CARE UNIT (ICU)-OUR EXPERIENCE. Asian Journal of Pharmaceutical and Clinical Research. 2017;10(4):417-21.

473. Prasad C, Bindra A, Singh P, Singh GP, Singh PK, Mathur P. Healthcare-associated Infections in Pediatric Patients in Neurotrauma Intensive Care Unit: A Retrospective Analysis. Indian Journal of Critical Care Medicine: Peer-reviewed, Official Publication of Indian Society of Critical Care Medicine. 2021;25(11):1308.

474. Chaudhary M, Payasi A. Incidence, prevalence and control of multidrug resistant (MDR) carbapenemase producing Acinetobacter baumanii in Indian intensive care units. Journal of Pharmacy Research. 2013;7(2):175-80.

475. Chittawatanarat K, Jaipakdee W, Chotirosniramit N, Chandacham K, Jirapongcharoenlap T. Microbiology, resistance patterns, and risk factors of mortality in ventilator-associated bacterial pneumonia in a Northern Thai tertiary-care university based general surgical intensive care unit. Infect Drug Resist. 2014;7:203-10.

476. Cicek AC, Karagoz A, Koksal E, Erturk A, Ozgumus OB, Koksal ZS, et al. A single clone Acinetobacter baumannii outbreak in a state hospital in Turkey. Jpn J Infect Dis. 2013;66(3):245-8.

477. Kalin G, Alp E, Akin A, Coskun R, Doganay M. Comparison of colistin and colistin/sulbactam for the treatment of multidrug resistant Acinetobacter baumannii ventilator-associated pneumonia. Infection. 2014;42(1):37-42.

478. Umut Safiye Say C, Emel C, Asegul Copur C, Halbay T, Cemal S. β-lactamase genes in carbapenem resistance Acinetobacter baumannii isolates from a Turkish university hospital. The Journal of Infection in Developing Countries. 2019;13(01).

479. Das R, Bhattacharyya I. MICROBIOLOGICAL FLORA AND THEIR ANTIBIOTIC SUSCEPTIBILITY- A STUDY UNDERTAKEN WITH ENDOTRACHEAL TUBE TIPS AND ENDOTRACHEAL ASPIRATES IN A TERTIARY CARE HOSPITAL IN KOLKATA. Journal of Evolution of Medical and Dental Sciences. 2018;7:2931-3.

480. Darvishnia D, Najafi N, Davoudi A, Jafari A, Mirzakhani M. Clinical, Laboratory and Microbiological Characteristics of Patients with Nosocomial Infections Admitted to Sari Fatemeh Zahra Heart Hospital, 2018-2019. Journal of Mazandaran University of Medical Sciences. 2022;32(210):108-15.

481. De Francesco MA, Ravizzola G, Peroni L, Bonfanti C, Manca N. Prevalence of multidrug-resistant Acinetobacter baumannii and Pseudomonas aeruginosa in an Italian hospital. J Infect Public Health. 2013;6(3):179-85.

482. Dedeić-Ljubović A, Granov Đ, Hukić M. Emergence of extensive drug-resistant (XDR) Acinetobacter baumanniiin the Clinical Center University of Sarajevo, Bosnia and Herzegovina. Med Glas (Zenica). 2015;12(2):169-76.

483. Direkel Ş, Çopur Çiçek A, Karagöz A, Ejder N, Oktay E, DelİAlİOĞLu N, et al. Antimicrobial Susceptibility and Molecular Characterization of Multidrug-Resistant Acinetobacter baumannii Isolated in an University Hospital. Mikrobiyoloji bülteni. 2016;50:522-34.

484. Ergönül Ö, Aydin M, Azap A, Başaran S, Tekin S, Kaya Ş, et al. Healthcare-associated Gram-negative bloodstream infections: antibiotic resistance and predictors of mortality. J Hosp Infect. 2016;94(4):381-5.

485. Eslami M, Shafiei M, Mirforughi SA, Rajabi A. Multiple carbapenemase gene production by Acinetobacter baumannii isolates from burn patients in Iran. Reviews in Medical Microbiology. 2019;30(2):90-4.

486. Negm EM, Mowafy SM, Mohammed AA, Amer MG, Tawfik AE, Ibrahim AE, et al. Antibiograms of intensive care units at an Egyptian tertiary care hospital. The Egyptian Journal of Bronchology. 2021;15:1-15.

487. Fallah A, Ahangarzadeh Rezaee M, Hasani A, Soroush Barhaghi MH, Samadi Kafil H. Frequency of bap and cpaA virulence genes in drug resistant clinical isolates of Acinetobacter baumannii and their role in biofilm formation. Iranian Journal of Basic Medical Sciences. 2017;20(8):849-55.

488. Farajnia S, Azhari F, Alikhani M, Hosseini M, Peymani A, Sohrabi N. Prevalence of PER and VEB Type Extended Spectrum Betalactamases among Multidrug Resistant Acinetobacter baumannii Isolates in North-West of Iran. Iranian journal of basic medical sciences. 2013;16:751-5.

489. Farsiani H, Mosavat A, Soleimanpour S, Nasab MN, Salimizand H, Jamehdar SA, et al. Limited genetic diversity and extensive antimicrobial resistance in clinical isolates of Acinetobacter baumannii in north-east Iran. J Med Microbiol. 2015;64(7):767-73.

490. Farzana R, Shamsuzzaman S, Mamun K. Isolation and molecular characterization of New Delhi metallo-beta-lactamase-1 producing superbug in Bangladesh. Journal of infection in developing countries. 2013;7:161-8.

491. Fernández Cuenca F, Sánchez Mdel C, Caballero-Moyano FJ, Vila J, Martínez-Martínez L, Bou G, et al. Prevalence and analysis of microbiological factors associated with phenotypic heterogeneous resistance to carbapenems in Acinetobacter baumannii. Int J Antimicrob Agents. 2012;39(6):472-7.

492. Khan F, Afsar HH, Afsar M, Jabeen K, Rasheed F, editors. Antibiotic Susceptibility Pattern of Acinetobacter Species Isolated from Critically ill Patients of a Tertiary Care Hospital. Med Forum; 2018.

493. Frikh M, Abdelhay L, Jalal K, Imad Y, Yassine B, Bouchra B, et al. Profile and Antibiotic Susceptibility of Bacteria Isolates in Burn Patients Hospitalized in a Moroccan Hospital: A Cross-sectional Study. Wounds. 2018;30(4):102-7.

494. Mboowa G, Aruhomukama D, Sserwadda I, Kitutu FE, Davtyan H, Owiti P, et al. Increasing antimicrobial resistance in surgical wards at mulago national referral hospital, Uganda, from 2014 to 2018—Cause for Concern? Tropical Medicine and Infectious Disease. 2021;6(2):82.

495. Gholami M, Hashemi A, Hakemi-Vala M, Goudarzi H, Hallajzadeh M. Efflux Pump Inhibitor Phenylalanine-Arginine Β-Naphthylamide Effect on the Minimum Inhibitory Concentration of Imipenem in Acinetobacter baumannii Strains Isolated From Hospitalized Patients in Shahid Motahari Burn Hospital, Tehran, Iran. Jundishapur J Microbiol. 2015;8(10):e19048.

496. Gupta R, Malik A, Rizvi M, Ahmed M. Presence of metallo-beta-lactamases (MBL), extended-spectrum beta-lactamase (ESBL) & AmpC positive non-fermenting Gram-negative bacilli among Intensive Care Unit patients with special reference to molecular detection of bla(CTX-M) & bla(AmpC) genes. Indian J Med Res. 2016;144(2):271-5.

497. Hasan MJ, Shamsuzzaman SM. Distribution of adeB and NDM-1 genes in multidrug resistant Acinetobacter baumannii isolated from infected wound of patients admitted in a tertiary care hospital in Bangladesh. Malays J Pathol. 2017;39(3):277-83.

498. Mostofa HA, Shamsuzzaman S, Hasan MM. Colistin susceptibility pattern in gram negative bacilli isolated from patients of dhaka medical college hospital with distribution of antibiotic resistance genes among them. bioRxiv. 2020:2020.04. 16.045906.

499. Heidary M, Salimi Chirani A, Khoshnood S, Eslami G, Atyabi SM, Nazem H, et al. Molecular detection of aminoglycoside-modifying enzyme genes in Acinetobacter baumannii clinical isolates. Acta Microbiol Immunol Hung. 2017;64(2):143-50.

500. Ben Lakhal H, M’Rad A, Naas T, Brahmi N. Antimicrobial susceptibility among pathogens Isolated in early-versus late-onset ventilator-associated pneumonia. Infectious Disease Reports. 2021;13(2):401-10.

501. Ara H. Prevalence of ESBL Encoding Genes in Acinetobacter baumannii Strains Isolated from Various Samples of a Tertiary Care Hospital in Mymensingh. Clin Case Rep Int 2021; 5. 2021;1251.

502. Tawfeeq HR, Rasheed MN, Hassan RH, Musleh MH, Nader MI. Molecular detection of blaoxa genes in Acinetobacter baumannii collected from patients with various infections. Biochem Cell Arch. 2020;20(1):1233-9.

503. Ali IA, Alsaadi LAS, Abbas SA. MOLECULAR DETECTION OF BIOFILM CODING GENES IN EXTENSIVELY DRUG-RESISTANT ACINETOBACTER BAUMANNII ISOLATED FROM IRAQI PATIENTS IN DIYALA. Biochemical & Cellular Archives. 2021;21.

504. Izadi B, Souzani R, Farahani A, Mehrabian S, Mohajeri P. Frequency of class 2 integrons in multidrug-resistant Acinetobacter baumannii isolated from patients in West of Iran. Annals of Tropical Medicine and Public Health. 2017;10(1):104.

505. Jalali Y, Sturdik I, Jalali M, Payer J. Isolated carbapenem resistant bacteria, their multidrug resistant profile, percentage of healthcare associated infection and associated mortality, in hospitalized patients in a University Hospital in Bratislava. CLINICAL STUDY. 2021;379:385.

506. Japoni-Nejad A, Sofian M, Belkum Av, Ghaznavi-Rad E. Nosocomial outbreak of extensively and pan drug-resistant Acinetobacter baumannii in tertiary hospital in central part of Iran. Jundishapur Journal of microbiology. 2013;6(8).

507. Jasemi S, Douraghi M, Adibhesami H, Zeraati H, Rahbar M, Boroumand MA, et al. Trend of extensively drug-resistant Acinetobacter baumannii and the remaining therapeutic options: a multicenter study in Tehran, Iran over a 3-year period. Lett Appl Microbiol. 2016;63(6):466-72.

508. Banihashemi K, Amirmozafari N, Mehregan I, Bakhtiari R, Sobouti B. Antibacterial effect of carbon nanotube containing chemical compounds on drug-resistant isolates of Acinetobacter baumannii. Iranian Journal of Microbiology. 2021;13(1):112.

509. Kapoor K, Jajoo M, Dublish S, Dabas V, Gupta S, Manchanda V. Intravenous colistin for multidrug-resistant gram-negative infections in critically ill pediatric patients. Pediatr Crit Care Med. 2013;14(6):e268-72.

510. Kapoor K, Jain S, Jajoo M, Dublish S, Dabas V, Manchanda V. Risk Factors and Predictors of Mortality in Critically ill Children with Extensively-Drug Resistant Acinetobacter baumannii Infection in a Pediatric Intensive Care Unit. Iranian journal of pediatrics. 2014;24(5):569-74.

511. Kara İ, Yildirim F, Bilaloglu B, Karamanlioglu D, Kayacan E, Dizbay M, et al. Comparison of the efficacy of colistin monotherapy and colistin combination therapies in the treatment of nosocomial pneumonia and ventilator-associated pneumonia caused by Acinetobacter baumannii. Southern African Journal of Critical Care. 2015;31(2):51-8.

512. Karmostaji A, Peerayeh S, Salmanian A. Distribution of OXA-Type Class D β-Lactamase Genes Among Nosocomial Multi Drug Resistant Acinetobacter baumannii Isolated in Tehran Hospitals. Jundishapur Journal of Microbiology. 2013;6.

513. Katsaragakis S, Markogiannakis H, Toutouzas KG, Drimousis P, Larentzakis A, Theodoraki EM, et al. Acinetobacter baumannii infections in a surgical intensive care unit: predictors of multi-drug resistance. World J Surg. 2008;32(6):1194-202.

514. Kaur A, Singh S. Prevalence of Extended Spectrum Betalactamase (ESBL) and Metallobetalactamase (MBL) Producing<i> Pseudomonas aeruginosa</i> and<i> Acinetobacter baumannii</i> Isolated from Various Clinical Samples. Journal of Pathogens. 2018;2018:6845985.

515. Salama K, Gad A, El Tatawy S. Sepsis profile and outcome of preterm neonates admitted to neonatal intensive care unit of Cairo University Hospital. Egyptian Pediatric Association Gazette. 2021;69:1-9.

516. Khatun MN, Farzana R, Lopes BS, Shamsuzzaman SM. Molecular characterization and resistance profile of nosocomial Acinetobacter baumannii intensive care unit of tertiary care hospital in Bangladesh. Bangladesh Med Res Counc Bull. 2015;41(2):101-7.

517. Khorsi K, Messai Y, Hamidi M, Ammari H, Bakour R. High prevalence of multidrug-resistance in Acinetobacter baumannii and dissemination of carbapenemase-encoding genes blaOXA-23-like, blaOXA-24-like and blaNDM-1 in Algiers hospitals. Asian Pac J Trop Med. 2015;8(6):438-46.

518. Khoshnood S, Eslami G, Hashemi A, Bahramian A, Heidary M, Yousefi N, et al. Distribution of Aminoglycoside Resistance Genes Among Acinetobacter baumannii Strains Isolated From Burn Patients in Tehran, Iran. Arch Pediatr Infect Dis. 2017;5(3):e57263.

519. Kooti S, Motamedifar M, Sarvari J. Antibiotic Resistance Profile and Distribution of Oxacillinase Genes Among Clinical Isolates of Acinetobacter baumannii in Shiraz Teaching Hospitals, 2012 - 2013. Jundishapur J Microbiol. 2015;8(8):e20215.

520. Kovacevic P, Zlojutro B, Kovacevic T, Baric G, Dragic S, Momcicevic D. Microorganisms Profile and Antibiotics Sensitivity Patterns in the Only Medical Intensive Care Unit in Bosnia and Herzegovina. Microb Drug Resist. 2019;25(8):1176-81.

521. Thirapanmethee K, Srisiri-A-Nun T, Houngsaitong J, Montakantikul P, Khuntayaporn P, Chomnawang MT. Prevalence of OXA-type β-lactamase genes among carbapenem-resistant Acinetobacter baumannii clinical isolates in Thailand. Antibiotics. 2020;9(12):864.

522. Lachhab Z, Frikh M, Maleb A, Kasouati J, Doghmi N, Ben Lahlou Y, et al. Bacteraemia in Intensive Care Unit: Clinical, Bacteriological, and Prognostic Prospective Study. Canadian Journal of Infectious Diseases and Medical Microbiology. 2017;2017:4082938.

523. Kakhandki L, Takpere AY, Bagali S, Wavare S, Karigoudar R, Shahapur PR. Antibiogram of Gram Negative Bacteria Isolated from the Skin and Soft Tissue Infections a Guide for Empirical Therapy to the Clinicians. J Pure Appl Microbiol. 2020;14(2):1353-8.

524. Leungtongkam U, Thummeepak R, Wongprachan S, Thongsuk P, Kitti T, Ketwong K, et al. Dissemination of blaOXA-23, blaOXA-24, blaOXA-58, and blaNDM-1 Genes of Acinetobacter baumannii Isolates from Four Tertiary Hospitals in Thailand. Microbial Drug Resistance. 2017;24(1):55-62.

525. Levy-Blitchtein S, Roca I, Plasencia-Rebata S, Vicente-Taboada W, Velásquez-Pomar J, Muñoz L, et al. Emergence and spread of carbapenem-resistant Acinetobacter baumannii international clones II and III in Lima, Peru. Emerg Microbes Infect. 2018;7(1):119.

526. Mahto M, Chaudhary M, Shah A, Show K, Moses F, Stewart A. High antibiotic resistance and mortality with Acinetobacter species in a tertiary hospital, Nepal. Public Health Action. 2021;11(1):13-7.

527. Maamar E, Alonso CA, Ferjani S, Jendoubi A, Hamzaoui Z, Jebri A, et al. NDM-1- and OXA-23-producing Acinetobacter baumannii isolated from intensive care unit patients in Tunisia. Int J Antimicrob Agents. 2018;52(6):910-5.

528. Mahdi AA, Abdullah RM. Molecular study of Carbapenime resistant genes in Acinetobacter baumannii that isolated from different clinical cases. Biochemical and Cellular Archives. 2020;20(1):1605-10.

529. Marie MA, Krishnappa LG, Alzahrani AJ, Mubaraki MA, Alyousef AA. A prospective evaluation of synergistic effect of sulbactam and tazobactam combination with meropenem or colistin against multidrug resistant Acinetobacter baumannii. Bosn J Basic Med Sci. 2015;15(4):24-9.

530. Abesamis GMM, Cruz JJV. Bacteriologic Profile of Burn Wounds at a Tertiary Government Hospital in the Philippines-UP-PGH ATR Burn Center. J Burn Care Res. 2019;40(5):658-68.

531. Mathlouthi N, Areig Z, Al Bayssari C, Bakour S, Ali El Salabi A, Ben Gwierif S, et al. Emergence of Carbapenem-Resistant Pseudomonas aeruginosa and Acinetobacter baumannii Clinical Isolates Collected from Some Libyan Hospitals. Microb Drug Resist. 2015;21(3):335-41.

532. Metan G, Demiraslan H, Kaynar LG, Zararsız G, Alp E, Eser B. Factors influencing the early mortality in haematological malignancy patients with nosocomial Gram negative bacilli bacteraemia: a retrospective analysis of 154 cases. The Brazilian Journal of Infectious Diseases. 2013;17(2):143-9.

533. Metan G, Pala Ç, Kaynar L, Cevahir F, Alp E. A nightmare for haematology clinics: extensively drug-resistant (XDR) Acinetobacter baumannnii. Infez Med. 2014;22(4):277-82.

534. Aghamali M, Gholizadeh P, Moaddab SR, Yousefi M, Asgharzadeh M, Ganbarov K, et al. Multi-Drug Resistant (MDR) and carbapenemase co-producing Gram-negative bacilli in northwest of Iran. Gene Reports. 2021;23:101181.

535. Talpur MTH, Shabir KU, Shabir KU, Katbar MT, Yaqoob U, Kashif S. Antibiotic susceptibility pattern in an intensive care unit of a tertiary care hospital of Pakistan. Rawal Medical Journal. 2020;45(1):17-.

536. KAR M, DUBEY A, SINGH R, SAHU C, PATEL SS, FATIMA N. Acinetobacter Meningitis: A Retrospective Study on its Incidence and Mortality Rates in Postoperative Patients at a Tertiary Care Centre in Northern India. Journal of Clinical & Diagnostic Research. 2023;17(1).

537. Modarresi F, Azizi O, Shakibaie MR, Motamedifar M, Valibeigi B, Mansouri S. Effect of iron on expression of efflux pump (adeABC) and quorum sensing (luxI, luxR) genes in clinical isolates of Acinetobacter baumannii. Apmis. 2015;123(11):959-68.

538. Mohajeri P, Esmailzadeh T, Torkaman S, Farahani A. Molecular Typing and Antimicrobial Susceptibility of Acinetobacter baumannii Isolates in Kermanshah City with Pulse Field Gel Electrophoresis (PFGE). Journal of Pharmaceutical Research International. 2018;20:1-8.

539. Mohajeri P, Farahani A, Feizabadi MM, Norozi B. Clonal evolution multi-drug resistant Acinetobacter baumannii by pulsed-field gel electrophoresis. Indian J Med Microbiol. 2015;33(1):87-91.

540. Mohajeri P, Farahani A, Feizabadi MM, Ketabi H, Abiri R, Najafi F. Antimicrobial susceptibility profiling and genomic diversity of Acinetobacter baumannii isolates: A study in western Iran. Iran J Microbiol. 2013;5(3):195-202.

541. Mohajeri P, Farahani A, Mehrabzadeh RS. Molecular characterization of multidrug resistant strains of Acinetobacter baumannii isolated from intensive care units in west of Iran. Journal of clinical and diagnostic research: JCDR. 2017;11(2):DC20.

542. Meybodi MME, Foroushani AR, Zolfaghari M, Abdollahi A, Alipour A, Mohammadnejad E, et al. Antimicrobial resistance pattern in healthcare-associated infections: investigation of in-hospital risk factors. Iranian Journal of Microbiology. 2021;13(2):178.

543. Mohd Rani F, NI AR, Ismail S, Abdullah FH, Othman N, Alattraqchi AG, et al. Prevalence and antimicrobial susceptibilities of Acinetobacter baumannii and non-baumannii Acinetobacters from Terengganu, Malaysia and their carriage of carbapenemase genes. J Med Microbiol. 2018;67(11):1538-43.

544. Khurshid M, Rasool MH, Ashfaq UA, Aslam B, Waseem M, Xu Q, et al. Dissemination of blaOXA-23-harbouring carbapenem-resistant Acinetobacter baumannii clones in Pakistan. Journal of global antimicrobial resistance. 2020;21:357-62.

545. Moisoiu A, Ionită M, Sârbu L, Stoica C, Grigoriu L. [Antibiotic resistance of Acinetobacter baumannii strains isolated from clinical specimens in the "Marius Nasta" Pneumology Institute, Bucharest]. Pneumologia. 2014;63(2):109-11.

546. Anvarinejad M, Khalifeh M, Mardaneh J, Pouladfar G, Dehyadegari MA. Bloodstream Infections in the South of Iran: Microbiological Profile and Antibiotic-Resistance Patterns of Isolated Bacteria. Crescent Journal of Medical & Biological Sciences. 2021;8(4).

547. Moosavian M, Ahmadi K, Shoja S, Mardaneh J, Shahi F, Afzali M. Antimicrobial resistance patterns and their encoding genes among clinical isolates of Acinetobacter baumannii in Ahvaz, Southwest Iran. MethodsX. 2020;7:101031.

548. Mahmoudi Monfared A, Rezaei A, Poursina F, Faghri J. Detection of Genes Involved in Biofilm Formation in MDR and XDR Acinetobacter baumannii Isolated from Human Clinical Specimens in Isfahan, Iran. Arch Clin Infect Dis. 2019;14(2):e85766.

549. Moosavian M, Sirous M, Shams N. Phenotypic and Genotypic Detection of Extended Spectrum β-lactamase and Carbapenemases Production Including bla TEM, bla PER and bla NDM-1 Genes Among Acinetobacter baumannii Clinical Isolates. Jundishapur J Microbiol. 2017;10(12):e58206.

550. Mohammed MA, Ahmed MT, Anwer BE, Aboshanab KM, Aboulwafa MM. Propranolol, chlorpromazine and diclofenac restore susceptibility of extensively drug-resistant (XDR)-Acinetobacter baumannii to fluoroquinolones. PloS one. 2020;15(8):e0238195.

551. Alavi-Moghaddam M, Dolati M, Javadi A, Saki BG, Karami-Zarandi M, Khoshnood S. Molecular detection of oxacillinase genes and typing of clinical isolates of Acinetobacter baumannii in Tehran, Iran. Journal of Acute Disease. 2020;9(1):33-9.

552. Mózes J, Ebrahimi F, Gorácz O, Miszti C, Kardos G. Effect of carbapenem consumption patterns on the molecular epidemiology and carbapenem resistance of Acinetobacter baumannii. J Med Microbiol. 2014;63(Pt 12):1654-62.

553. SHASHIKALA N, SHANKAR M, MADHURA N, MYTHRI K, KOWSALYA R. Clinical and Microbiological Profile of Tracheal Aspirates in Chronic Kidney Disease Patients. Journal of Clinical & Diagnostic Research. 2021;15(2).

554. El Hamzaoui N, Barguigua A, Larouz S, Maouloua M. Epidemiology of burn wound bacterial infections at a Meknes hospital, Morocco. New Microbes and New Infections. 2020;38:100764.

555. Azim N, Al-Harbi M, Al-Zaban M, Nofal M, Somily A. Prevalence and Antibiotic Susceptibility among Gram Negative Bacteria Isolated from Intensive Care Units at a Tertiary Care Hospital in Riyadh, Saudi Arabia. Journal of Pure and Applied Microbiology. 2019;13:201-8.

556. Najar Peerayeh S, Karmostaji A. Molecular Identification of Resistance Determinants, Integrons and Genetic Relatedness of Extensively Drug Resistant Acinetobacter baumannii Isolated From Hospitals in Tehran, Iran. Jundishapur journal of microbiology. 2015;8(7):e27021-e.

557. Attia NM, Elbaradei A. Fluoroquinolone resistance conferred by gyrA, parC mutations, and AbaQ efflux pump among Acinetobacter baumannii clinical isolates causing ventilator-associated pneumonia. Acta microbiològica et immunologica Hungarica. 2020;67(4):234-8.

558. Moradi N, Kazemi N, Ghaemi M, Mirzaei B. Frequency and antimicrobial resistance pattern of bacterial isolates from patients with COVID-19 in two hospitals of Zanjan. Iranian Journal of Microbiology. 2021;13(6):769.

559. Tuan Anh N, Nga TVT, Tuan HM, Tuan NS, Y DM, Vinh Chau NV, et al. Molecular epidemiology and antimicrobial resistance phenotypes of Acinetobacter baumannii isolated from patients in three hospitals in southern Vietnam. J Med Microbiol. 2017;66(1):46-53.

560. Nikasa P, Abdi-Ali A, Rahmani-Badi A, Al-Hamad A. In vitro evaluation of proton motive force-dependent efflux pumps among multidrug resistant Acinetobacter baumannii isolated from patients at Tehran hospitals. Jundishapur journal of microbiology. 2013;6(7):1G.

561. Niranjan DK, Singh NP, Manchanda V, Rai S, Kaur IR. Multiple carbapenem hydrolyzing genes in clinical isolates of Acinetobacter baumannii. Indian J Med Microbiol. 2013;31(3):237-41.

562. Fahim NAE. Prevalence and antimicrobial susceptibility profile of multidrug-resistant bacteria among intensive care units patients at Ain Shams University Hospitals in Egypt—a retrospective study. Journal of the Egyptian Public Health Association. 2021;96(1):1-10.

563. Noori M, Mohsenzadeh B, Bahramian A, Shahi F, Mirzaei H, Khoshnood S. Characterization and frequency of antibiotic resistance related to membrane porin and efflux pump genes among <i>Acinetobacter baumannii</i> strains obtained from burn patients in Tehran, Iran. Journal of Acute Disease. 2019;8(2):63-6.

564. Zahra N, Zeshan B, Qadri MMA, Ishaq M, Afzal M, Ahmed N. Phenotypic and genotypic evaluation of antibiotic resistance of Acinetobacter baumannii bacteria isolated from surgical intensive care unit patients in Pakistan. Jundishapur Journal of Microbiology. 2021;14(4).

565. Odewale G, Adefioye OJ, Ojo J, Adewumi FA, Olowe OA. Multidrug Resistance of Acinetobacter Baumannii in Ladoke Akintola University Teaching Hospital, Osogbo, Nigeria. European journal of microbiology & immunology. 2016;6(3):238-43.

566. Idowu OJ, Onipede AO, Orimolade AE, Akinyoola LA, Babalola GO. Extended-spectrum beta-lactamase orthopedic wound infections in Nigeria. Journal of global infectious diseases. 2011;3(3):211.

567. Oncul O, Oksuz S, Acar A, Ulkur E, Turhan V, Uygur F, et al. Nosocomial infection characteristics in a burn intensive care unit: analysis of an eleven-year active surveillance. Burns. 2014;40(5):835-41.

568. Perween N, Sehgal S, Prakash SK. Geographical patterns in antimicrobial resistance of acinetobacter in clinical isolates. J Clin Diagn Res. 2014;8(4):Dc10-2.

569. Ghimire R, Gupte H, Shrestha S, Thekkur P, Kharel S, Kattel H, et al. High drug resistance among Gram-negative bacteria in sputum samples from an intensive care unit in Nepal. Public Health Action. 2021;11(1):64-9.

570. Makharita RR, El-Kholy I, Hetta HF, Abdelaziz MH, Hagagy FI, Ahmed AA, et al. Antibiogram and genetic characterization of carbapenem-resistant gram-negative pathogens incriminated in healthcare-associated infections. Infection and drug resistance. 2020:3991-4002.

571. Rafei R, Dabboussi F, Hamze M, Eveillard M, Lemarié C, Mallat H, et al. First report of blaNDM-1-producing Acinetobacter baumannii isolated in Lebanon from civilians wounded during the Syrian war. International Journal of Infectious Diseases. 2014;21:21-3.

572. Amiri R, Fozouni L. Antibacterial Effects of Peganum harmala Seed Extracts on Drug-resistant Clinical Isolates of Acinetobacter baumannii in North of Iran. Jundishapur Journal of Natural Pharmaceutical Products. 2020;15(2).

573. Ruiz J, Núñez ML, Pérez J, Simarro E, Martínez-Campos L, Gómez J. Evolution of resistance among clinical isolates of Acinetobacter over a 6-year period. Eur J Clin Microbiol Infect Dis. 1999;18(4):292-5.

574. Rynga D, Shariff M, Deb M. Phenotypic and molecular characterization of clinical isolates of Acinetobacter baumannii isolated from Delhi, India. Annals of clinical microbiology and antimicrobials. 2015;14(1):1-8.

575. Ozyurt S, Kostakoglu U, Yildiz I, Erturk A, Sonmez E, Sahin U, et al. Investigation of the clonal associations in Acinetobacter Baumannii strains isolated from the respiratory samples of patients in a tertiary research hospital. Nigerian journal of clinical practice. 2020;23(8):1155-62.

576. Jayashree S, Rajeshwari K, Wadekar MD. Drug Resistance Mechanism among Acinetobacter Species. Journal of Pure and Applied Microbiology. 2021.

577. Said HS, Benmahmod AB, Ibrahim RH. Co-production of AmpC and extended spectrum beta-lactamases in cephalosporin-resistant Acinetobacter baumannii in Egypt. World J Microbiol Biotechnol. 2018;34(12):189.

578. Salehi B, Ghalavand Z, Mohammadzadeh M, Maleki DT, Kodori M, Kadkhoda H. Clonal relatedness and resistance characteristics of OXA-24 and -58 producing carbapenem-resistant Acinetobacter baumannii isolates in Tehran, Iran. Journal of Applied Microbiology. 2019;127(5):1421-9.

579. Salimizand H, Menbari S, Ramazanzadeh R, Khonsha M, Vahedi MS. DNA fingerprinting and antimicrobial susceptibility pattern of clinical and environmental Acinetobacter baumannii isolates: a multicentre study. J Chemother. 2014:1973947814y0000000227.

580. Salimizand H, Noori N, Meshkat Z, Ghazvini K, Amel SJ. Prevalence of Acinetobacter baumannii harboring ISAba1/blaOXA-23-like family in a burn center. Burns. 2015;41(5):1100-6.

581. TIPPARTHI SK, AKULA S, RAJKUMAR H, REDDY AR, MANDERWAD GP. Co-expression of 16S rRNA Methyltransferase and Carbapenemase in Multidrug Resistant Gram Negative Bacteria. Journal of Clinical & Diagnostic Research. 2020;14(11).

582. Rajkumari S, Pradhan S, Sharma D, Jha B. Prevalence and Antibiogram of Acinetobacter Species Isolated from Various Clinical Samples in a Tertiary Care Hospital. Journal of College of Medical Sciences-Nepal. 2020;16(1):26-32.

583. Gunasekaran S, Mahadevaiah S. Healthcare-associated infection in intensive care units: overall analysis of patient criticality by acute physiology and chronic health evaluation IV scoring and pathogenic characteristics. Indian Journal of Critical Care Medicine: Peer-reviewed, Official Publication of Indian Society of Critical Care Medicine. 2020;24(4):252.

584. Yadav SK, Bhujel R, Hamal P, Mishra SK, Sharma S, Sherchand JB. Burden of multidrug-resistant Acinetobacter baumannii infection in hospitalized patients in a tertiary care hospital of Nepal. Infection and drug resistance. 2020:725-32.

585. Sarikhani Z, Nazari R, Nateghi Rostami M. First report of OXA-143-lactamase producing Acinetobacter baumannii in Qom, Iran. Iran J Basic Med Sci. 2017;20(11):1282-6.

586. Sepahvand S, Madani M, Davarpanah MA, Ghandehari F. Evaluation antibiotic resistance and presence of bla OXA-51, bla OXA-58 and bla OXA-23 genes in Acinetobacter baumannii strains via multiplex PCR. Pakistan Journal of Pharmaceutical Sciences. 2021;34(5).

587. Sepahvand S, Madani M, Davarpanah MA, Ghandehari F. Molecular Study of Colistin Resistance Genes in Acinetobacter baumannii Isolated from Patients in the Intensive Care Unit (ICU). Ethiopian Journal of Health Development. 2022;36(1).

588. Shoja S, Moosavian M, Peymani A, Tabatabaiefar MA, Rostami S, Ebrahimi N. Genotyping of carbapenem resistant Acinetobacter baumannii isolated from tracheal tube discharge of hospitalized patients in intensive care units, Ahvaz, Iran. Iran J Microbiol. 2013;5(4):315-22.

589. Shrestha S, Tada T, Miyoshi-Akiyama T, Ohara H, Shimada K, Satou K, et al. Molecular epidemiology of multidrug-resistant Acinetobacter baumannii isolates in a university hospital in Nepal reveals the emergence of a novel epidemic clonal lineage. Int J Antimicrob Agents. 2015;46(5):526-31.

590. Sileem AE, Said AM, Meleha MS. Acinetobacter baumannii in ICU patients: A prospective study highlighting their incidence, antibiotic sensitivity pattern and impact on ICU stay and mortality. Egyptian Journal of Chest Diseases and Tuberculosis. 2017;66(4):693-8.

591. Singla P, Sikka R, Deep A, Chaudhary U. Phenotypic Detection And Prevalence Of Metallo Β-Lactamases (Mbls) In Carbapenem Resistant Isolates Of Acinetobacter Species At A Tertiary Care Hospital In North India. Int J Pharm Med Bio Sci. 2013;2:85-91.

592. Sohrabi N, Farajnia S, Akhi MT, Nahaei MR, Naghili B, Peymani A, et al. Prevalence of OXA-type β-lactamases among Acinetobacter baumannii isolates from Northwest of Iran. Microb Drug Resist. 2012;18(4):385-9.

593. Souli M, Kontopidou FV, Koratzanis E, Antoniadou A, Giannitsioti E, Evangelopoulou P, et al. In vitro activity of tigecycline against multiple-drug-resistant, including pan-resistant, gram-negative and gram-positive clinical isolates from Greek hospitals. Antimicrob Agents Chemother. 2006;50(9):3166-9.

594. Mahich S, Angurana SK, Sundaram V, Gautam V. Epidemiology, microbiological profile, and outcome of culture positive sepsis among outborn neonates at a tertiary hospital in Northern India. The Journal of Maternal-Fetal & Neonatal Medicine. 2022;35(25):7948-56.

595. Tahmasebi Birgani M, Bijanzadeh M, Ansari H. Antibiotic Characterization of Acinetobacter baumannii Isolated from Clinical Samples and Production of Recombinant OmpA from Resistant Strains. Jundishapur J Microbiol. 2018;11(12):e78773.

596. Tarashi S, Goudarzi H, Erfanimanesh S, Pormohammad A, Hashemi A. Phenotypic and Molecular Detection of Metallo-Beta-Lactamase Genes Among Imipenem Resistant Pseudomonas aeruginosa and Acinetobacter baumannii Strains Isolated From Patients with Burn Injuries. Archives of Clinical Infectious Diseases. 2016;inpress.

597. Tayebi Z, Doust RH, Rahimi MK, Siadat SD, Goudarzi M. Distribution of different carbapenemase genes in carbapenem-resistant Acinetobacter baumannii strains isolated from intensive care: A two year multi-center study in Tehran, Iran. Gene Reports. 2019;15:100382.

598. Denysko TV, Nazarchuk OA, Gruzevskyi O, Bahniuk NÀ, Dmytriiev DV, Chornopyschuk RM, et al. In vitro evaluation of the antimicrobial activity of antiseptics against clinical Acinetobacter baumannii strains isolated from combat wounds. Frontiers in Microbiology. 2022;13:932467.

599. Tewari R, Chopra D, Wazahat R, Dhingra S, Dudeja M. Antimicrobial Susceptibility Patterns of an Emerging Multidrug Resistant Nosocomial Pathogen: Acinetobacter baumannii. Malays J Med Sci. 2018;25(3):129-34.

600. Nguyen TT, Nguyen KT, Pham ST, Pham XD, Nguyen T. Hospital-acquired pneumonia in an intensive care unit in Vietnam: clinical characteristics and pathogenic bacteria. Pharmaceutical Sciences Asia. 2020;47(4).

601. Reenu T, Jeannette W, Sharona S, Sithembiso V. Prevalence, antimicrobial susceptibility profiles and case fatality rates of Acinetobacter Baumannii sepsis in a neonatal unit. The Journal of Infection in Developing Countries. 2018;12(04).

602. Tohamy ST, Aboshanab KM, El-Mahallawy HA, El-Ansary MR, Afifi SS. Prevalence of multidrug-resistant Gram-negative pathogens isolated from febrile neutropenic cancer patients with bloodstream infections in Egypt and new synergistic antibiotic combinations. Infect Drug Resist. 2018;11:791-803.

603. Ngoc Van TT, Quang-Thinh T, Cù T, Nhac Vu HT. INVESTIGATION OF THE ANTIBIOTIC RESISTANCE: THE CASE OF BUU DIEN GENERAL HOSPITAL IN HO CHI MINH CITY. International journal of pharmacy and pharmaceutical sciences, vol 9(11), pages 116-119. International Journal of Pharmacy and Pharmaceutical Sciences. 2019:116-9.

604. Trehan A, Totadri S, Gautam V, Bansal D, Ray P. Invasive bacterial infections in a pediatric oncology unit in a tertiary care center. Indian Journal of Cancer. 2014;51(4):428-31.

605. Tuğba ARSLAN GÜLEN Aİ, İlker ÖDEMİŞ2, Üner KAYABAŞ. Acinetobacter baumannii infections and antibiotic resistance in hospitalized patients

in an education and research hospital: a six-year analysis. FLORA. 2020;4(25):563-71.

606. Leungtongkam U, Thummeepak R, Kitti T, Tasanapak K, Wongwigkarn J, Styles KM, et al. Genomic analysis reveals high virulence and antibiotic resistance amongst phage susceptible Acinetobacter baumannii. Scientific Reports. 2020;10(1):16154.

607. Kondratiuk V, Jones B, Kovalchuk V, Kovalenko I, Ganiuk V, Kondratiuk O, et al. Phenotypic and genotypic characterization of antibiotic resistance in military hospital-associated bacteria from war injuries in the Eastern Ukraine conflict between 2014 and 2020. Journal of Hospital Infection. 2021;112:69-76.

608. Nag V, Kombade S, Sharma A, Tak V, Priyadarshi K. The multidrug resistant profile of Acinetobacter spp. isolated from clinical samples in a tertiary care hospital from western Rajasthan, India. International Journal of Infectious Diseases. 2020;101:79.

609. Vaidya JB, Charde VN, Arun B. Prevalence of Multi drug resistance Acinetobacter baumannii in Central India (Nagpur). J Pharm Res. 2015;9:517-21.

610. Roy S, Viswanathan R, Singh A, Das P, Basu S. Gut colonization by multidrug-resistant and carbapenem-resistant Acinetobacter baumannii in neonates. Eur J Clin Microbiol Infect Dis. 2010;29(12):1495-500.

611. Wang X, Zhang L, Sun A, Yang X, Sang W, Jiang Y, et al. Acinetobacter baumannii bacteraemia in patients with haematological malignancy: a multicentre retrospective study from the Infection Working Party of Jiangsu Society of Hematology. Eur J Clin Microbiol Infect Dis. 2017;36(7):1073-81.

612. Zaha DC, Bungau S, Aleya S, Tit DM, Vesa CM, Popa AR, et al. What antibiotics for what pathogens? The sensitivity spectrum of isolated strains in an intensive care unit. Sci Total Environ. 2019;687:118-27.

613. Moulana Z, Babazadeh A, Eslamdost Z, Shokri M, Ebrahimpour S. Phenotypic and genotypic detection of metallo-beta-lactamases in Carbapenem resistant Acinetobacter baumannii. Caspian journal of internal medicine. 2020;11(2):171.

614. Karamya ZA, Youssef A, Adra A, Karah N, Kanj SS, Elamin W, et al. High rates of antimicrobial resistance among clinical isolates from microbiology laboratories in Syria. Journal of Infection. 2021;82(2):e8-e10.

615. Zarifi E, Eslami G, Khaledi A, Vakili M, Vazini H, Zandi H. Prevalence of ESBLs in Acinetobacter baumannii isolated from intensive care unit (ICU) of Ghaem hospital, Mashhad, Iran. Journal of Pure and Applied Microbiology. 2017;11(2):811-9.

616. Zilberberg MD, Kollef MH, Shorr AF. Secular trends in Acinetobacter baumannii resistance in respiratory and blood stream specimens in the United States, 2003 to 2012: a survey study. Journal of hospital medicine. 2016;11(1):21-6.

617. Al-Agamy MH, Jeannot K, El-Mahdy TS, Shibl AM, Kattan W, Plésiat P, et al. First Detection of GES-5 Carbapenemase-Producing Acinetobacter baumannii Isolate. Microb Drug Resist. 2017;23(5):556-62.

618. Japoni-Nejad A, Mood EH, Ehsani P, Sardari S, Heravi FS, Bouzari S, et al. Identification and characterization of the type II toxin-antitoxin systems in the carbapenem-resistant Acinetobacter baumannii. Microbial Pathogenesis. 2021;158:105052.

619. Al-Obeid S, Jabri L, Al-Agamy M, Al-Omari A, Shibl A. Epidemiology of extensive drug resistant Acinetobacter baumannii (XDRAB) at Security Forces Hospital (SFH) in Kingdom of Saudi Arabia (KSA). J Chemother. 2015;27(3):156-62.

620. Al-Sweih NA, Al-Hubail M, Rotimi VO. Three distinct clones of carbapenem-resistant Acinetobacter baumannii with high diversity of carbapenemases isolated from patients in two hospitals in Kuwait. Journal of Infection and Public Health. 2012;5(1):102-8.

621. Altun Ş, Koçak Tufan Z, Altun B, Önde U, Kınıklı S, Demiröz AP. Growing OXA-23 type strains among carbapenem-resistant Acinetobacter baumannii and tigecycline as an alternate combination therapy. Turk J Med Sci. 2016;46(6):1894-9.

622. Grisold AJ, Luxner J, Bedenić B, Diab-Elschahawi M, Berktold M, Wechsler-Fördös A, et al. Diversity of Oxacillinases and sequence types in Carbapenem-resistant Acinetobacter baumannii from Austria. International Journal of Environmental Research and Public Health. 2021;18(4):2171.

623. Armin S, Karimi A, Fallah F, Rafiei Tabatabaii S, Hoseini Alfatemi SM, Khiabanirad P, et al. Antimicrobial Resistance Patterns of Acinetobacter baumannii, Pseudomonas aeruginosa and Staphylococcus aureus Isolated From Patients With Nosocomial Infections Admitted to Tehran Hospitals. Arch Pediatr Infect Dis. 2015;3(4):e32554.

624. Asadollahi P, Akbari M, Soroush S, Taherikalani M, Asadollahi K, Sayehmiri K, et al. Antimicrobial resistance patterns and their encoding genes among Acinetobacter baumannii strains isolated from burned patients. Burns. 2012;38(8):1198-203.

625. Paudel A, Devkota SP, Shrestha A, Shah AK. Prevalence of Colistin-resistant Gram-negative Isolates Carrying the mcr-1 Gene among Patients Visiting a Tertiary Care Center. JNMA: Journal of the Nepal Medical Association. 2020;58(232):983.

626. Baadani A, Thawadi S, Elkhizzi N, Omrani A. Prevalence of colistin and tigecycline resistance in Acinetobacter baumannii clinical isolates from 2 hospitals in Riyadh Region over a 2-year period. Saudi medical journal. 2013;34:248-53.

627. Bahador A, Raoofian R, Taheri M, Pourakbari B, Hashemizadeh Z, Hashemi FB. Multidrug resistance among Acinetobacter baumannii isolates from Iran: changes in antimicrobial susceptibility patterns and genotypic profile. Microb Drug Resist. 2014;20(6):632-40.

628. Bakour S, Touati A, Sahli F, Ameur AA, Haouchine D, Rolain JM. Antibiotic resistance determinants of multidrug-resistant Acinetobacter baumannii clinical isolates in Algeria. Diagn Microbiol Infect Dis. 2013;76(4):529-31.

629. Castilho SRA, Godoy CSM, Guilarde AO, Cardoso JL, André MCP, Junqueira-Kipnis AP, et al. Acinetobacter baumannii strains isolated from patients in intensive care units in Goiânia, Brazil: Molecular and drug susceptibility profiles. PLoS One. 2017;12(5):e0176790.

630. Liang-Yu C, Kuo SC, Liu CY, Luo BS, Huang LJ, Lee YT, et al. Difference in imipenem, meropenem, sulbactam, and colistin nonsusceptibility trends among three phenotypically undifferentiated Acinetobacter baumannii complex in a medical center in Taiwan, 1997-2007. J Microbiol Immunol Infect. 2011;44(5):358-63.

631. Cherkaoui A, Emonet S, Renzi G, Schrenzel J. Characteristics of multidrug-resistant Acinetobacter baumannii strains isolated in Geneva during colonization or infection. Ann Clin Microbiol Antimicrob. 2015;14:42.

632. Chmielarczyk A, Pobiega M, Ziółkowski G, Pomorska-Wesołowska M, Romaniszyn D, Krawczyk L, et al. Severe infections caused by multidrug-resistant non-fermentative bacilli in southern Poland. Adv Clin Exp Med. 2018;27(3):401-7.

633. Chmielarczyk A, Pilarczyk-Żurek M, Kamińska W, Pobiega M, Romaniszyn D, Ziółkowski G, et al. Molecular Epidemiology and Drug Resistance of Acinetobacter baumannii Isolated from Hospitals in Southern Poland: ICU as a Risk Factor for XDR Strains. Microb Drug Resist. 2016;22(4):328-35.

634. Cicek AC, Saral A, Iraz M, Ceylan A, Duzgun AO, Peleg AY, et al. OXA- and GES-type β-lactamases predominate in extensively drug-resistant Acinetobacter baumannii isolates from a Turkish University Hospital. Clin Microbiol Infect. 2014;20(5):410-5.

635. Cikman A, Gulhan B, Aydin M, Ceylan MR, Parlak M, Karakecili F, et al. In vitro Activity of Colistin in Combination with Tigecycline against Carbapenem-Resistant Acinetobacter baumannii Strains Isolated from Patients with Ventilator-Associated Pneumonia. Int J Med Sci. 2015;12(9):695-700.

636. Decousser JW, Jansen C, Nordmann P, Emirian A, Bonnin RA, Anais L, et al. Outbreak of NDM-1-producing Acinetobacter baumannii in France, January to May 2013. Euro Surveill. 2013;18(31).

637. Defaee S, Farasatinasab M, Makani M, Rahimipour H, Alinia P, Defaee S. Prevalence of Gram Negative Infections by Acinetobacter and Pseudomonas Severely Resistant to Antibiotic Susceptibility Based on Minimum Growth Inhibitor Concentration. Journal of Pharmaceutical Research International. 2019:1-6.

638. Deylam Salehi M, Ferdosi-Shahandashti E, Yahyapour Y, Khafri S, Pournajaf A, Rajabnia R. Integron-Mediated Antibiotic Resistance in Acinetobacter baumannii Isolated from Intensive Care Unit Patients, Babol, North of Iran. Biomed Res Int. 2017;2017:7157923.

639. Direkel Ş, Uzunoğlu E, Keleş S, Yapar K. Antibiotic Resistance Rates of Acinetobacter Baumannii Strains Isolated from Various Clinical Samples in Giresun Prof. Dr. Atilla ilhan Ozdemir State Hospital. Gazi Medical Journal. 2015.

640. Ece G, Samlioglu P, Atalay S, Kose S. Evaluation of the in vitro colistin susceptibility of Pseudomonas aeruginosa and Acinetobacter baumannii strains at a tertiary care centre in Western Turkey. Infez Med. 2014;22(1):36-40.

641. Elabd FM, Al-Ayed MS, Asaad AM, Alsareii SA, Qureshi MA, Musa HA. Molecular characterization of oxacillinases among carbapenem-resistant Acinetobacter baumannii nosocomial isolates in a Saudi hospital. J Infect Public Health. 2015;8(3):242-7.

642. Ergin A, Hascelik G, Eser OK. Molecular characterization of oxacillinases and genotyping of invasive Acinetobacter baumannii isolates using repetitive extragenic palindromic sequence-based polymerase chain reaction in Ankara between 2004 and 2010. Scand J Infect Dis. 2013;45(1):26-31.

643. Farshadzadeh Z, Hashemi FB, Rahimi S, Pourakbari B, Esmaeili D, Haghighi MA, et al. Wide distribution of carbapenem resistant Acinetobacter baumannii in burns patients in Iran. Frontiers in Microbiology. 2015;6(1146).

644. Ahmadi Khatiri F, Fahimzad SA, Fallah F, Armin S, Azimi L. Frequency of MDR Acinetobacter baumannii and the Most Common OXA-type Genes in Multiple Drug-Resistant Strains Isolated from Patients in Tabriz Imam Reza Hospital. Journal of Mazandaran University of Medical Sciences. 2020;30(187):117-26.

645. Fonseca EL, Scheidegger E, Freitas FS, Cipriano R, Vicente AC. Carbapenem-resistant Acinetobacter baumannii from Brazil: role of carO alleles expression and blaOXA-23 gene. BMC Microbiol. 2013;13:245.

646. Galani I, Orlandou K, Moraitou H, Petrikkos G, Souli M. Colistin/daptomycin: an unconventional antimicrobial combination synergistic in vitro against multidrug-resistant Acinetobacter baumannii. Int J Antimicrob Agents. 2014;43(4):370-4.

647. Galani I, Kontopidou F, Souli M, Rekatsina PD, Koratzanis E, Deliolanis J, et al. Colistin susceptibility testing by Etest and disk diffusion methods. Int J Antimicrob Agents. 2008;31(5):434-9.

648. Goudarzi M, Azimi H. Dissemination of Classes 1, 2, and 3 Integrons in Acinetobacter baumannii Strains Recovered from Intensive Care Units Using Polymerase Chain Reaction-Restriction Fragment Length Polymorphism. Jundishapur J Microbiol. 2017;10(5):e13100.

649. SANTHI G, KUMAR PK, SRIDHARAN KS. Effectiveness of Minocycline in Multidrug Resistance Gram Negative Bacilli: A Cross-sectional Study. Journal of Clinical & Diagnostic Research. 2023;17(3).

650. Güven T, Yilmaz G, Güner HR, Kaya Kalem A, Eser F, Taşyaran MA. Increasing resistance of nosocomial Acinetobacter baumannii: are we going to be defeated? Turk J Med Sci. 2014;44(1):73-8.

651. Haddad FA, Van Horn K, Carbonaro C, Aguero-Rosenfeld M, Wormser GP. Evaluation of antibiotic combinations against multidrug-resistant Acinetobacter baumannii using the E-test. Eur J Clin Microbiol Infect Dis. 2005;24(8):577-9.

652. Handal R, Qunibi L, Sahouri I, Juhari M, Dawodi R, Marzouqa H, et al. Characterization of Carbapenem-Resistant Acinetobacter baumannii Strains Isolated from Hospitalized Patients in Palestine. Int J Microbiol. 2017;2017:8012104.

653. Hasanin A, Mukhtar A, El-adawy A, Elazizi H, Lotfy A, Nassar H, et al. Ventilator associated pneumonia caused by extensive-drug resistant Acinetobacter species: Colistin is the remaining choice. Egyptian Journal of Anaesthesia. 2016;32(3):409-13.

654. Héritier C, Dubouix A, Poirel L, Marty N, Nordmann P. A nosocomial outbreak of Acinetobacter baumannii isolates expressing the carbapenem-hydrolysing oxacillinase OXA-58. J Antimicrob Chemother. 2005;55(1):115-8.

655. Gajic I, Jovicevic M, Milic M, Kekic D, Opavski N, Zrnic Z, et al. Clinical and molecular characteristics of OXA-72-producing Acinetobacter baumannii ST636 outbreak at a neonatal intensive care unit in Serbia. Journal of Hospital Infection. 2021;112:54-60.

656. Izdebski R, Fiett J, Hryniewicz W, Gniadkowski M. Molecular analysis of Acinetobacter baumannii isolates from invasive infections in 2009 in Poland. Journal of clinical microbiology. 2012;50(11):3813-5.

657. Jankowski CA, Balada-Llasat J-M, Raczkowski M, Pancholi P, Goff DA. A stewardship approach to combating multidrug-resistant Acinetobacter baumannii infections with minocycline. Infectious Diseases in Clinical Practice. 2012;20(3):184-7.

658. Jiménez-Guerra G, Heras-Cañas V, Gutiérrez-Soto M, Del Pilar Aznarte-Padial M, Expósito-Ruiz M, Navarro-Marí JM, et al. Urinary tract infection by Acinetobacter baumannii and Pseudomonas aeruginosa: evolution of antimicrobial resistance and therapeutic alternatives. J Med Microbiol. 2018;67(6):790-7.

659. Lim JS, Cho HH, Kim S, Kim J, Kwon K, Park JW, et al. The Genetic Characteristics of Multidrug-resistant Acinetobacter baumannii Coproducing 16S rRNA Methylase armA and Carbapenemase OXA-23. Journal of Bacteriology and Virology. 2013;43:27-36.

660. Kabic J, Novovic K, Kekic D, Trudic A, Opavski N, Dimkic I, et al. Comparative genomics and molecular epidemiology of colistin-resistant Acinetobacter baumannii. Computational and Structural Biotechnology Journal. 2023;21:574-85.

661. Karah N, Dwibedi CK, Sjöström K, Edquist P, Johansson A, Wai SN, et al. Novel aminoglycoside resistance transposons and transposon-derived circular forms detected in carbapenem-resistant Acinetobacter baumannii clinical isolates. Antimicrobial agents and chemotherapy. 2016;60(3):1801-18.

662. Karaoglan I, Zer Y, Bosnak VK, Mete AO, Namiduru M. In vitro synergistic activity of colistin with tigecycline or β-lactam antibiotic/β-lactamase inhibitor combinations against carbapenem-resistant Acinetobacter baumannii. J Int Med Res. 2013;41(6):1830-7.

663. Kheshti R, Pourabbas B, Mosayebi M, Vazin A. In vitro activity of colistin in combination with various antimicrobials against Acinetobacter baumannii species, a report from South Iran. Infection and drug resistance. 2018;12:129-35.

664. Kusradze I, Diene SM, Goderdzishvili M, Rolain JM. Molecular detection of OXA carbapenemase genes in multidrug-resistant Acinetobacter baumannii isolates from Iraq and Georgia. Int J Antimicrob Agents. 2011;38(2):164-8.

665. Lee H-Y, Hsu S-Y, Hsu J-F, Chen C-L, Wang Y-H, Chiu C-H. Risk factors and molecular epidemiology of Acinetobacter baumannii bacteremia in neonates. Journal of Microbiology, Immunology and Infection. 2018;51(3):367-76.

666. Leelasupasri S, Santimaleeworagun W, Jitwasinkul T. Antimicrobial Susceptibility among Colistin, Sulbactam, and Fosfomycin and a Synergism Study of Colistin in Combination with Sulbactam or Fosfomycin against Clinical Isolates of Carbapenem-Resistant Acinetobacter baumannii. J Pathog. 2018;2018:3893492.

667. Mahdian S, Sadeghifard N, Pakzad I, Ghanbari F, Soroush S, Azimi L, et al. Acinetobacter baumannii clonal lineages I and II harboring different carbapenem-hydrolyzing-β-lactamase genes are widespread among hospitalized burn patients in Tehran. J Infect Public Health. 2015;8(6):533-42.

668. Malekzadegan Y, Abdi A, Heidari H, Moradi M, Rastegar E, Sedigh Ebrahim-Saraie H. In vitro activities of colistin, imipenem and ceftazidime against drug-resistant Pseudomonas aeruginosa and Acinetobacter baumannii isolates in the south of Iran. BMC Res Notes. 2019;12(1):301.

669. Manageiro V, Jones-Dias D, Ferreira E, Louro D, Caniça M. Genetic diversity and clonal evolution of carbapenem-resistant Acinetobacter baumannii isolates from Portugal and the dissemination of ST118. Int J Antimicrob Agents. 2012;40(5):398-403.

670. Maraki S, Mavros MN, Kofteridis DP, Samonis G, Falagas ME. Epidemiology and antimicrobial sensitivities of 536 multi-drug-resistant gram-negative bacilli isolated from patients treated on surgical wards. Surg Infect (Larchmt). 2012;13(5):326-31.

671. Shabban M, Fahim NAE, Montasser K, El Magd NMA. Resistance to colistin mediated by mcr-1 among multidrug resistant Gram negative pathogens at a tertiary care hospital, Egypt. J Pure Appl Microbiol. 2020;14(2):1125-32.

672. Mathlouthi N, Ben Lamine Y, Somai R, Bouhalila-Besbes S, Bakour S, Rolain JM, et al. Incidence of OXA-23 and OXA-58 Carbapenemases Coexpressed in Clinical Isolates of Acinetobacter baumannii in Tunisia. Microb Drug Resist. 2018;24(2):136-41.

673. Mavroidi A, Likousi S, Palla E, Katsiari M, Roussou Z, Maguina A, et al. Molecular identification of tigecycline- and colistin-resistant carbapenemase-producing Acinetobacter baumannii from a Greek hospital from 2011 to 2013. J Med Microbiol. 2015;64(9):993-7.

674. Jiang M, Chen X, Liu S, Zhang Z, Li N, Dong C, et al. Epidemiological analysis of multidrug-resistant Acinetobacter baumannii isolates in a tertiary hospital over a 12-year period in China. Frontiers in Public Health. 2021;9:707435.

675. Miyasaki Y, Morgan MA, Chan RC, Nichols WS, Hujer KM, Bonomo RA, et al. In vitro activity of antibiotic combinations against multidrug-resistant strains of Acinetobacter baumannii and the effects of their antibiotic resistance determinants. FEMS Microbiol Lett. 2012;328(1):26-31.

676. Mohammadi M, Soroush S, Delfani S, Pakzad I, Abbaszadeh A, Bahmani M, et al. Distribution of Class D Carbapenemase and Extended-Spectrum β-Lactamase Genes among Acinetobacter Baumannii Isolated from Burn Wound and Ventilator Associated Pneumonia Infections. J Clin Diagn Res. 2017;11(7):Dc19-dc23.

677. Sannathimmappa MB, Nambiar V, Aravindakshan R. Antibiotic resistance pattern of Acinetobacter baumannii strains: A retrospective study from Oman. Saudi Journal of Medicine & Medical Sciences. 2021;9(3):254.

678. Mohanty S, Maurya V, Gaind R, Deb M. Phenotypic characterization and colistin susceptibilities of carbapenem-resistant of Pseudomonas aeruginosa and Acinetobacter spp. J Infect Dev Ctries. 2013;7(11):880-7.

679. Varshochi M, Hasani A, Shahverdi PP, Ghavghani FR, Matin S. Risk Factors for the Antibiotic Resistant Gram-Negative Bacilli Associated Infections in Burn Patients and the In-Vitro Susceptibility of Colistin. Archives of Clinical Infectious Diseases. 2020;15(3).

680. Kamali M, Manshouri S, Bagheri Y, Rostami M, Mahmoudi MK, Moradnejad P, et al. Prevalence and antibiotic resistance of Acinetobacter baumannii among patients in postcardiac surgery intensive care units of Rajaei Hospital, Tehran. Medical journal of the Islamic Republic of Iran. 2020;34:4.

681. Mosavat A, Soleimanpour S, Farsiani H, Salimizand H, Kebriaei A, Amel Jamehdar S, et al. Moderate Genetic Diversity with Extensive Antimicrobial Resistance Among Multidrug-Resistant Acinetobacter baumannii in a Referral Hospital in Northeast Iran. Jundishapur J Microbiol. 2018;11(7):e14412.

682. Nakwan N, Wannaro J, Thongmak T, Pornladnum P, Saksawad R, Nakwan N, et al. Safety in treatment of ventilator-associated pneumonia due to extensive drug-resistant Acinetobacter baumannii with aerosolized colistin in neonates: a preliminary report. Pediatr Pulmonol. 2011;46(1):60-6.

683. Banoub NG, Saleh SE, Helal HS, Aboshanab KM. Antibiotics combinations and chitosan nanoparticles for combating multidrug resistance Acinetobacter baumannii. Infection and Drug Resistance. 2021:3327-39.

684. Obeidat N, Jawdat F, Al-Bakri AG, Shehabi AA. Major biologic characteristics of Acinetobacter baumannii isolates from hospital environmental and patients' respiratory tract sources. Am J Infect Control. 2014;42(4):401-4.

685. Oikonomou O, Sarrou S, Papagiannitsis CC, Georgiadou S, Mantzarlis K, Zakynthinos E, et al. Rapid dissemination of colistin and carbapenem resistant Acinetobacter baumannii in Central Greece: mechanisms of resistance, molecular identification and epidemiological data. BMC Infect Dis. 2015;15:559.

686. Oliva A, Garzoli S, De Angelis M, Marzuillo C, Vullo V, Mastroianni CM, et al. In-Vitro Evaluation of Different Antimicrobial Combinations with and without Colistin Against Carbapenem-Resistant Acinetobacter Baumannii. Molecules. 2019;24(5).

687. Oteo J, García-Estébanez C, Migueláñez S, Campos J, Martí S, Vila J, et al. Genotypic diversity of imipenem resistant isolates of Acinetobacter baumannii in Spain. Journal of Infection. 2007;55(3):260-6.

688. Pankuch GA, Seifert H, Appelbaum PC. Activity of doripenem with and without levofloxacin, amikacin, and colistin against Pseudomonas aeruginosa and Acinetobacter baumannii. Diagn Microbiol Infect Dis. 2010;67(2):191-7.

689. Papadimitriou-Olivgeris M, Fligou F, Spiliopoulou A, Koutsileou K, Kolonitsiou F, Spyropoulou A, et al. Risk factors and predictors of carbapenem-resistant Pseudomonas aeruginosa and Acinetobacter baumannii mortality in critically ill bacteraemic patients over a 6-year period (2010-15): antibiotics do matter. J Med Microbiol. 2017;66(8):1092-101.

690. Pasanen T, Koskela S, Mero S, Tarkka E, Tissari P, Vaara M, et al. Rapid molecular characterization of Acinetobacter baumannii clones with rep-PCR and evaluation of carbapenemase genes by new multiplex PCR in Hospital District of Helsinki and Uusimaa. PLoS One. 2014;9(1):e85854.

691. Bogaerts P, Naas T, Wybo I, Bauraing C, Soetens O, Piérard D, et al. Outbreak of infection by carbapenem-resistant Acinetobacter baumannii producing the carbapenemase OXA-58 in Belgium. Journal of clinical microbiology. 2006;44(11):4189-92.

692. Pongpech P, Amornnopparattanakul S, Panapakdee S, Fungwithaya S, Nannha P, Dhiraputra C, et al. Antibacterial activity of carbapenem-based combinations againts multidrug-resistant Acinetobacter baumannii. J Med Assoc Thai. 2010;93(2):161-71.

693. Queenan AM, Pillar CM, Deane J, Sahm DF, Lynch AS, Flamm RK, et al. Multidrug resistance among Acinetobacter spp. in the USA and activity profile of key agents: results from CAPITAL Surveillance 2010. Diagn Microbiol Infect Dis. 2012;73(3):267-70.

694. Karki R, Lamichhane S, Basnet BB, Dahal A, Awal BK, Mishra SK. In vitro antimicrobial synergy testing of extensively drug-resistant clinical isolates at an organ transplant center in Nepal. Infection and Drug Resistance. 2021:1669-77.

695. Reddy T, Chopra T, Marchaim D, Pogue JM, Alangaden G, Salimnia H, et al. Trends in antimicrobial resistance of Acinetobacter baumannii isolates from a metropolitan Detroit health system. Antimicrob Agents Chemother. 2010;54(5):2235-8.

696. Kara Ali R, Surme S, Balkan II, Salihoglu A, Sahin Ozdemir M, Ozdemir Y, et al. An eleven-year cohort of bloodstream infections in 552 febrile neutropenic patients: resistance profiles of Gram-negative bacteria as a predictor of mortality. Annals of hematology. 2020;99:1925-32.

697. Khoshnood S, Sadeghifard N, Mahdian N, Heidary M, Mahdian S, Mohammadi M, et al. Antimicrobial resistance and biofilm formation capacity among Acinetobacter baumannii strains isolated from patients with burns and ventilator‐associated pneumonia. Journal of Clinical Laboratory Analysis. 2023;37(1):e24814.

698. Salehi B, Goudarzi H, Nikmanesh B, Houri H, Alavi-Moghaddam M, Ghalavand Z. Emergence and characterization of nosocomial multidrug-resistant and extensively drug-resistant Acinetobacter baumannii isolates in Tehran, Iran. J Infect Chemother. 2018;24(7):515-23.

699. Sanal L, Yilmaz N, Sen S, Cesur S. DETECTION OF SYNERGISTIC ANTIMICROBIAL ACTIVITIES BETWEEN DORIPENEM, TIGECYCLINE AND COLISTIN AGAINST MULTI-DRUG-RESISTANT ACINETOBACTER BAUMANNII STRAINS OBTAINED FROM PATIENTS IN INTENSIVE CARE UNITS. Acta Medica Mediterranea. 2017;33(4):691-7.

700. Savari M, Ekrami A, Shoja S, Bahador A. Plasmid borne Carbapenem-Hydrolyzing Class D β-Lactamases (CHDLs) and AdeABC efflux pump conferring carbapenem-tigecycline resistance among Acinetobacter baumannii isolates harboring TnAbaRs. Microb Pathog. 2017;104:310-7.

701. Mostafavi SN, Rostami S, Nokhodian Z, Ataei B, Cheraghi A, Ataabadi P, et al. Antibacterial resistance patterns of Acinetobacter baumannii complex: The results of Isfahan antimicrobial resistance surveillance-1 program. Asian Pacific Journal of Tropical Medicine. 2021;14(7):316-22.

702. Seifert H, Stefanik D, Sutcliffe JA, Higgins PG. In-vitro activity of the novel fluorocycline eravacycline against carbapenem non-susceptible Acinetobacter baumannii. Int J Antimicrob Agents. 2018;51(1):62-4.

703. Senok A, Garaween G, Raji A, Khubnani H, Kim Sing G, Shibl A. Genetic relatedness of clinical and environmental Acinetobacter baumanii isolates from an intensive care unit outbreak. Journal of infection in developing countries. 2015;9:665-9.

704. Sepahvand S, Davarpanah MA, Roudgari A, Bahador A, Karbasizade V, Kargar Jahromi Z. Molecular evaluation of colistin-resistant gene expression changes in Acinetobacter baumannii with real-time polymerase chain reaction. Infection and drug resistance. 2017;10:455-62.

705. Sepahvand S, Doudi M, Davarpanah M, Bahador A, Ahmadi M. Analyzing pmrA and pmrB genes in Acinetobacter baumannii resistant to colistin in Shahid Rajai Shiraz, Iran Hospital by PCR: First report in Iran. Pakistan journal of pharmaceutical sciences. 2016;29:1401-6.

706. Sepahvand S, Darvishi M, Mokhtari M, Ali Davarpanah M. Evaluation of genetic diversity of colistin-resistant Acinetobacter baumannii by BOX-PCR and ERIC-PCR: the first report. Future Microbiology. 2022;17(12):917-30.

707. Dong SX, Wang JT, Chang SC. Activities of doripenem against nosocomial bacteremic drug-resistant Gram-negative bacteria in a medical center in Taiwan. J Microbiol Immunol Infect. 2012;45(6):459-64.

708. Mostafa SH, Saleh SE, Hamed SM, Aboshanab KM. Febrile illness of bacterial etiology in a public fever hospital in Egypt: High burden of multidrug resistance and WHO priority Gram negative pathogens. Germs. 2022;12(1):75.

709. Afhami S, Borumand MA, Bazzaz NE, Saffar H, Hadadi A, Nezhad MJ, et al. Antimicrobial resistance pattern of Acinetobacter; a multicenter study, comparing European Committee on Antimicrobial Susceptibility Testing (EUCAST) and the Clinical and Laboratory Standards Institute (CLSI); evaluation of susceptibility testing methods for polymyxin. Immunopathologia Persa. 2020;7(1):e04-e.

710. Shoja S, Moosavian M, Rostami S, Abbasi F, Tabatabaiefar MA, Peymani A. Characterization of Oxacillinase and Metallo-β-Lactamas Genes and Molecular Typing of Clinical Isolates of Acinetobacter baumannii in Ahvaz, South-West of Iran. Jundishapur journal of microbiology. 2016;9(5):e32388-e.

711. Shoja S, Moosavian M, Rostami S, Farahani A, Peymani A, Ahmadi K, et al. Dissemination of carbapenem-resistant Acinetobacter baumannii in patients with burn injuries. Journal of the Chinese Medical Association. 2017;80(4):245-52.

712. Shokri D, Rabbani Khorasgani M, Fatemi SM, Soleimani-Delfan A. Resistotyping, phenotyping and genotyping of New Delhi metallo-β-lactamase (NDM) among Gram-negative bacilli from Iranian patients. J Med Microbiol. 2017;66(4):402-11.

713. Soubirou JF, Gault N, Alfaiate T, Lolom I, Tubach F, Andremont A, et al. Ventilator-associated pneumonia due to carbapenem-resistant Gram-negative bacilli in an intensive care unit without carbapenemase-producing Enterobacteriaceae or epidemic Acinetobacter baumannii. Scand J Infect Dis. 2014;46(3):215-20.

714. Spence RP, Towner KJ, Henwood CJ, James D, Woodford N, Livermore DM. Population structure and antibiotic resistance of Acinetobacter DNA group 2 and 13TU isolates from hospitals in the UK. J Med Microbiol. 2002;51(12):1107-12.

715. Spiliopoulou A, Jelastopulu E, Vamvakopoulou S, Bartzavali C, Kolonitsiou F, Anastassiou ED, et al. In vitro activity of tigecycline and colistin against A. baumannii clinical bloodstream isolates during an 8-year period. J Chemother. 2015;27(5):266-70.

716. Srivastava R, Agarwal J, Srivastava S, Kumar M, Singh M. Multidrug resistant Gram-negative bacilli from neonatal septicaemia at a tertiary care centre in North India: a phenotypic and genotypic study. Indian J Med Microbiol. 2014;32(1):97-8.

717. Singh S, Sahu C, Patel SS, Singh A, Yaduvanshi N. A comparative in vitro sensitivity study of “Ceftriaxone–Sulbactam–EDTA” and various antibiotics against Gram-negative bacterial isolates from intensive care unit. Indian Journal of Critical Care Medicine: Peer-reviewed, Official Publication of Indian Society of Critical Care Medicine. 2020;24(12):1213.

718. Tan TY, Ng LS, Tan E, Huang G. In vitro effect of minocycline and colistin combinations on imipenem-resistant Acinetobacter baumannii clinical isolates. J Antimicrob Chemother. 2007;60(2):421-3.

719. Strateva TV, Sirakov I, Stoeva TJ, Stratev A, Peykov S. Phenotypic and Molecular Characteristics of Carbapenem-Resistant Acinetobacter baumannii Isolates from Bulgarian Intensive Care Unit Patients. Microorganisms. 2023;11(4):875.

720. Tsioutis C, Karageorgos SA, Stratakou S, Soundoulounaki S, Karabetsos DA, Kouyentakis G, et al. Clinical characteristics, microbiology and outcomes of external ventricular drainage-associated infections: The importance of active treatment. J Clin Neurosci. 2017;42:54-8.

721. Tunyapanit W, Pruekprasert P, Laoprasopwattana K, Chelae S. Antimicrobial susceptibility of Acinetobacter baumannii isolated from hospital patients. Sci Asia. 2014;40(1):28.

722. Uzunoglu E, Direkel S, Kocbiyik M, Uludag SK, Cicek AC. Co-existance of isaba1/blaoxa-51/23 is increasing in carbapenem rersistant Acinetobacter baumannii isolates in Turkey. Acta Medica Mediterr. 2017;33(6):1001.

723. Vakili B, Fazeli H, Shoaei P, Yaran M, Ataei B, Khorvash F, et al. Detection of colistin sensitivity in clinical isolates of Acinetobacter baumannii in Iran. J Res Med Sci. 2014;19(Suppl 1):S67-70.

724. Le Minh V, Thi Khanh Nhu N, Vinh Phat V, Thompson C, Huong Lan NP, Thieu Nga TV, et al. In vitro activity of colistin in antimicrobial combination against carbapenem-resistant Acinetobacter baumannii isolated from patients with ventilator-associated pneumonia in Vietnam. J Med Microbiol. 2015;64(10):1162-9.

725. Sheng W-H, Wang J-T, Li S-Y, Lin Y-C, Cheng A, Chen Y-C, et al. Comparative in vitro antimicrobial susceptibilities and synergistic activities of antimicrobial combinations against carbapenem-resistant Acinetobacter species: Acinetobacter baumannii versus Acinetobacter genospecies 3 and 13TU. Diagnostic microbiology and infectious disease. 2011;70(3):380-6.

726. Wattal C, Raveendran R, Goel N, Oberoi JK, Rao BK. Ecology of blood stream infection and antibiotic resistance in intensive care unit at a tertiary care hospital in North India. Braz J Infect Dis. 2014;18(3):245-51.

727. Yavaş S, Yetkin MA, Kayaaslan B, Baştuğ A, Aslaner H, But A, et al. Investigating the in vitro synergistic activities of several antibiotic combinationsagainst carbapenem-resistant Acinetobacter baumannii isolates. Turk J Med Sci. 2016;46(3):892-6.

728. Zeka AN, Poirel L, Sipahi OR, Bonnin RA, Arda B, Ozinel M, et al. GES-type and OXA-23 carbapenemase-producing Acinetobacter baumannii in Turkey. J Antimicrob Chemother. 2014;69(4):1145-6.

729. Zhou H, Pi BR, Yang Q, Yu YS, Chen YG, Li LJ, et al. Dissemination of imipenem-resistant Acinetobacter baumannii strains carrying the ISAba1 blaOXA-23 genes in a Chinese hospital. J Med Microbiol. 2007;56(Pt 8):1076-80.

730. Ziółkowski G, Pawłowska I, Krawczyk L, Wojkowska-Mach J. Antibiotic consumption versus the prevalence of multidrug-resistant Acinetobacter baumannii and Clostridium difficile infections at an ICU from 2014-2015. J Infect Public Health. 2018;11(5):626-30.

731. Ghalavand Z, Eslami G, Hashemi A, Sadredinamin M, Yousefi N, Dehbanipour R. Characterization of sequence types and mechanisms of resistance to tigecycline among acinetobacter baumannii isolated from children. Current Microbiology. 2022;79(9):285.

732. Mavroidi A, Katsiari M, Palla E, Likousi S, Roussou Z, Nikolaou C, et al. Investigation of Extensively Drug-Resistant blaOXA-23-Producing Acinetobacter baumannii Spread in a Greek Hospital. Microb Drug Resist. 2017;23(4):488-93.

733. Porwal R, Ghafur A, Vidyalakshmi P, Kannaian P, Arivazhaghan P. Neurosurgical Meningitis: Clinico-Microbiological Profile and Treatment Outcome from a Tertiary Care Center in India. Indian Journal of Neurosurgery. 2015;4(01):002-7.

734. Mahmoudi S, Mahzari M, Banar M, Pourakbari B, Haghi Ashtiani MT, Mohammadi M, et al. Antimicrobial resistance patterns of Gram-negative bacteria isolated from bloodstream infections in an Iranian referral paediatric hospital: A 5.5-year study. J Glob Antimicrob Resist. 2017;11:17-22.
