# Supplementary material for: Prevalence of colistin resistance in clinical isolates of Acinetobacter baumannii: a systematic review and meta-analysis
Source: Antimicrob Resist Infect Control. 2024 Feb 28;13:24. doi: 10.1186/s13756-024-01376-7 (PMC10902961; doi:10.1186/s13756-024-01376-7)
Supplement: Supplementary file 2 — Additional file 2: Table S2. Subgroup meta-analysis [file 13756_2024_1376_MOESM2_ESM.docx]

Table 1. Proportion of Colistin resistance

| **Subgroups** | | **No. of**  **study** | **Poled resistance rate (95% CI)** | **% weight** | **Heterogeneity test,**  **I^2^ (%)** | **Heterogeneity test, *P***  **value** | **Heterogeneity between groups (P-Value)** |
| --- | --- | --- | --- | --- | --- | --- | --- |
|  | **Overall effects** |  | 0.04 (0.03-0.05) | 100 | 95.50 | 0.00 |  |
| **Continent** | **Asia** | 234 | 0.04 (0.03-0.05) | 61.37 | 93.92 | 0.00 | 0.008 |
|  | **Africa** | 27 | 0.03 (0.01-0.07) | 6.81 | 89.18 | 0.00 |  |
|  | **South America** | 16 | 0.06 (0.01-0.15) | 3.77 | 88.93 | 0.00 |  |
|  | **Eastern Europe** | 61 | 0.01 (0.00-0.02) | 7.66 | 89.39 | 0.00 |  |
|  | **Western Europe** | 29 | 0.07 (0.04-0.11) | 15.64 | 98.17 | 0.00 |  |
|  | **North America** | 18 | 0.04 (0.01-0.08) | 4.76 | 89.66 | 0.00 |  |
| **Year of sample collection** | **2001_2011** | 61 | 0.02 (0.01-0.04) | 19.10 | 91.47 | 0.00 | 0.00 |
|  | **2012_2022** | 260 | 0.05 (0.03-0.06) | 80.90 | 95.85 | 0.00 |  |
| **Guideline** | **CLSI** | 324 | 0.04 (0.03-0.05) | 81.69 | 94.88 | 0.00 | 0.195 |
|  | **EUCAT** | 74 | 0.05 (0.03-0.07) | 18.31 | 96.02 | 0.00 |  |
| **AST Method** | **Agar dilution** | 38 | 0.01 (0.00-0.02) | 5.30 | 91.81 | 0.00 | 0.00 |
|  | **Broth microdilution** | 398 | 0.04 (0.03-0.05) | 54.05 | 95.31 | 0.00 |  |
|  | **Disk diffusion** | 187 | 0.02 (0.02-0.03) | 25.28 | 92.50 | 0.00 |  |
|  | **E-test** | 111 | 0.02 (0.01-0.03) | 15.07 | 90.75 | 0.00 |  |
| **Quality score** | **Low** | 8 | 0.09 (0.00-0.25) | 1.33 | 71.02 | 0.00 | 0.00 |
|  | **Moderate** | 138 | 0.05 (0.03-0.06) | 33.24 | 95.74 | 0.00 |  |
|  | **High** | 252 | 0.04 (0.03-0.05) | 65.43 | 95.06 | 0.00 |  |
| **Hospital ward** | **Burn** | 6 | 0.02 (0.00-0.05) | 6.25 | 71.18 | 0.00 | 0.00 |
|  | **ICU** | 80 | 0.04 (0.02- 0.06) | 82.42 | 91.74 | 0.00 |  |
|  | **NICU** | 9 | 0.00 (0.00-0.00) | 8.11 | 0.00 | 0.95 |  |
|  | **PICU** | 3 | 0.04 (0.00-0.09) | 3.22 | 0.00 | 0.00 |  |
| **Sample** | **Non respiratory** | 57 | 0.02 (0.01-0.04) | 73.55 | 85.81 | 0.00 | 0.16 |
|  | **respiratory** | 18 | 0.01 (0.00-0.03) | 26.45 | 87.21 | 0.00 |  |
| **Source of acquired infection** | **HAI** | 345 | 0.04 (0.03-0.05) | 96.46 | 95.15 | 0.00 | 0.691 |
|  | **CAI** | 13 | 0.05 (0.00-0.14) | 3.54 | 95.21 | 0.00 |  |
